# A First-Time-In-Human Phase I Clinical Trial of Bispecific Antibody-Targeted, Paclitaxel-Packaged Bacterial Minicells

**DOI:** 10.1371/journal.pone.0144559

**Published:** 2015-12-11

**Authors:** Benjamin J. Solomon, Jayesh Desai, Mark Rosenthal, Grant A. McArthur, Scott T. Pattison, Stacey L. Pattison, Jennifer MacDiarmid, Himanshu Brahmbhatt, Andrew M. Scott

**Affiliations:** 1 Department of Hematology and Medical Oncology, Peter MacCallum Cancer Centre, Melbourne, Victoria, Australia; 2 Medical Oncology, Royal Melbourne Hospital, Melbourne, Victoria, Australia; 3 Cancer Therapeutics, EnGeneIC Ltd, Sydney, New South Wales, Australia; 4 Olivia Newton-John Cancer and Wellness Centre, Austin Hospital, Heidelberg, Victoria, Australia; 5 Ludwig Institute for Cancer Research, Austin Hospital, Heidelberg, Victoria, Australia; Catalan Institute of Oncology, SPAIN

## Abstract

**Background:**

We have harnessed a novel biological system, the bacterial minicell, to deliver cancer therapeutics to cancer cells. Preclinical studies showed that epidermal growth factor receptor (EGFR)-targeted, paclitaxel-loaded minicells (^EGFR^minicells_Pac_) have antitumor effects in xenograft models. To examine the safety of the minicell delivery system, we initiated a first-time-in-human, open-label, phase I clinical study of ^EGFR^minicells_Pac_ in patients with advanced solid tumors.

**Methodology:**

Patients received 5 weekly infusions followed by a treatment free week. Seven dose levels (1x10^8^, 1x10^9^, 3x10^9^, 1x10^10^, 1.5x10^10^, 2x10^10^, 5x10^10^) were evaluated using a 3+3 dose-escalation design. Primary objectives were safety, tolerability and determination of the maximum tolerated dose. Secondary objectives were assessment of immune/inflammatory responses and antitumor activity.

**Principal Findings:**

Twenty eight patients were enrolled, 22 patients completed at least one cycle of ^EGFR^minicells_Pac_; 6 patients did not complete a cycle due to rapidly progressive disease. A total of 236 doses was delivered over 42 cycles, with a maximum of 45 doses administered to a single patient. Most common treatment-related adverse events were rigors and pyrexia. No deaths resulted from treatment-related adverse events and the maximum tolerated dose was defined as 1x10^10 EGFR^minicells_Pac_. Surprisingly, only a mild self-limiting elevation in the inflammatory cytokines IL-6, IL-8 and TNFα and anti-inflammatory IL-10 was observed. Anti-LPS antibody titers peaked by dose 3 and were maintained at that level despite repeat dosing with the bacterially derived minicells. Ten patients (45%; n = 22) achieved stable disease as their best response.

**Conclusions/Significance:**

This is the first study in humans of a novel biological system that can provide targeted delivery of a range of chemotherapeutic drugs to solid tumor cells. Bispecific antibody-targeted minicells, packaged with the chemotherapeutic paclitaxel, were shown to be safe in patients with advanced solid tumors with modest clinical efficacy observed. Further study in Phase II trials is planned.

**Trial Registration:**

Australian New Zealand Clinical Trials Registry ACTRN12609000672257

## Introduction

Conventional systemic therapy for cancer requires large concentrations of drug or antibody to achieve a therapeutic benefit. This is due to the indiscriminate bio-distribution of the drug which results in significant toxicity to normal tissues. Since most drugs do not specifically target tumor cells, this limits the therapeutic benefit that can be achieved. Targeted delivery of cancer therapies has potential therefore to increase anti-tumor efficacy and to reduce treatment toxicities.

Previously we had reported that minicells, being 400 ± 20 nanometer (nm) anucleate nanoparticles produced by the inactivation of the genes that control normal bacterial cell division at an equatorial septation site, can be packaged with therapeutically significant concentrations of a range of chemotherapeutics [1], siRNAs or shRNAs [2]. Further, these drug or siRNA-packaged minicells can be targeted to tumor cell surface receptors via attachment of bispecific antibodies (BsAb) to the minicell surface (S1 Fig). One arm of the BsAb has specificity to the O-polysaccharide component of the lipopolysaccharide (LPS) of the minicell and the other arm can be directed to a tumor cell-surface receptor [1].

Following intravenous (IV) administration, minicells preferentially extravasate into the tumor microenvironment (passive targeting) due to the leaky vasculature associated with most solid tumors [3], thereby avoiding normal tissue. In addition, dysfunctional lymphatic drainage results in retention of nanoparticles in the tumor microenvironment. This phenomenon is the enhanced permeability and retention (EPR) effect [4, 5]. The minicells then selectively target cancer cells via BsAbs where, following receptor engagement, they are endocytosed and degraded in intracellular lysosomes (active targeting). The cytotoxic drug, packaged within the minicell, is then released internally and allows the cancer cell to ‘commit suicide’ when the payload (in this study paclitaxel) is delivered. Hence, the drug loaded targeted minicells exert their main effect via intracellular delivery of the cytotoxic payload and not by blockade of the tumor cell-surface receptor with which the BsAb engages. Preclinical studies of minicells packaged with cytotoxic drugs in murine xenograft models resulted in tumor stabilization or regression [1]. Similarly, in canine endogenous tumor studies with doxorubicin-packaged minicells, marked tumor regression was observed in two dogs with advanced non-Hodgkin’s lymphoma [1].

Expression of the epidermal growth factor receptor (EGFR) in a large percentage of solid tumor types is associated with aggressive disease and poor clinical prognosis. In normal and malignant cells, activation of EGFR cascades has multiple consequences, such as cell growth, differentiation, and proliferation. The EGFR signaling pathway may also promote malignant transformation, angiogenesis, and metastatic dissemination [6]. To block activation of this receptor, targeted therapies such as monoclonal antibodies have been developed and approved including cetuximab and panitumumab in the treatment of metastatic colorectal cancer [7]. EGFR was chosen as a target, in this study, as it is frequently expressed at high levels in many epithelial malignancies. Although expression is identified in some normal tissues, tumor expression is typically higher than that seen in normal tissue. Despite the presence of EGFR on normal tissue, healthy vasculature prevents the minicells from leaving the circulation to reach EGFR on normal tissue. In contrast, in tumor tissue with leaky vasculature the minicells are able to leave blood vessels to enter tumor tissue and are retained by poor lymphatic drainage (enhanced permeability and retention effect). This provides the basis for selective delivery to tumor tissue.

Paclitaxel is a taxane drug used to treat a number of solid tumor types including breast, bladder, pancreatic, prostate and lung. Its mechanism of action is to bind to tubulin, thereby stabilizing microtubules. The resulting microtubule/paclitaxel complex does not have the ability to disassemble. This adversely affects cell function because the shortening and lengthening of microtubules (termed dynamic instability) is necessary for their function as a mechanism to transport other cellular components such as chromosomes during their replication [8]. Paclitaxel was the chosen chemotherapeutic to be packaged into the minicells for this first-time-in-human study as it has broad activity across many epithelial malignancies including cancers such as lung cancer and head and neck cancer that have high levels of EGFR expression.

In this first-time-in-human study, we aimed to assess the safety and tolerability of EGFR-targeted, paclitaxel-packaged minicells (designated ^EGFR^minicells_Pac_) at escalating doses in patients with refractory solid malignancies. We also aimed to determine the immune and cytokine response to ^EGFR^minicells_Pac_ following repeat dosing. This is the first report of a human clinical trial using minicells for targeted delivery of the cytotoxic compound paclitaxel to solid tumors.

## Results

### Patient Characteristics

**Fig 1:**
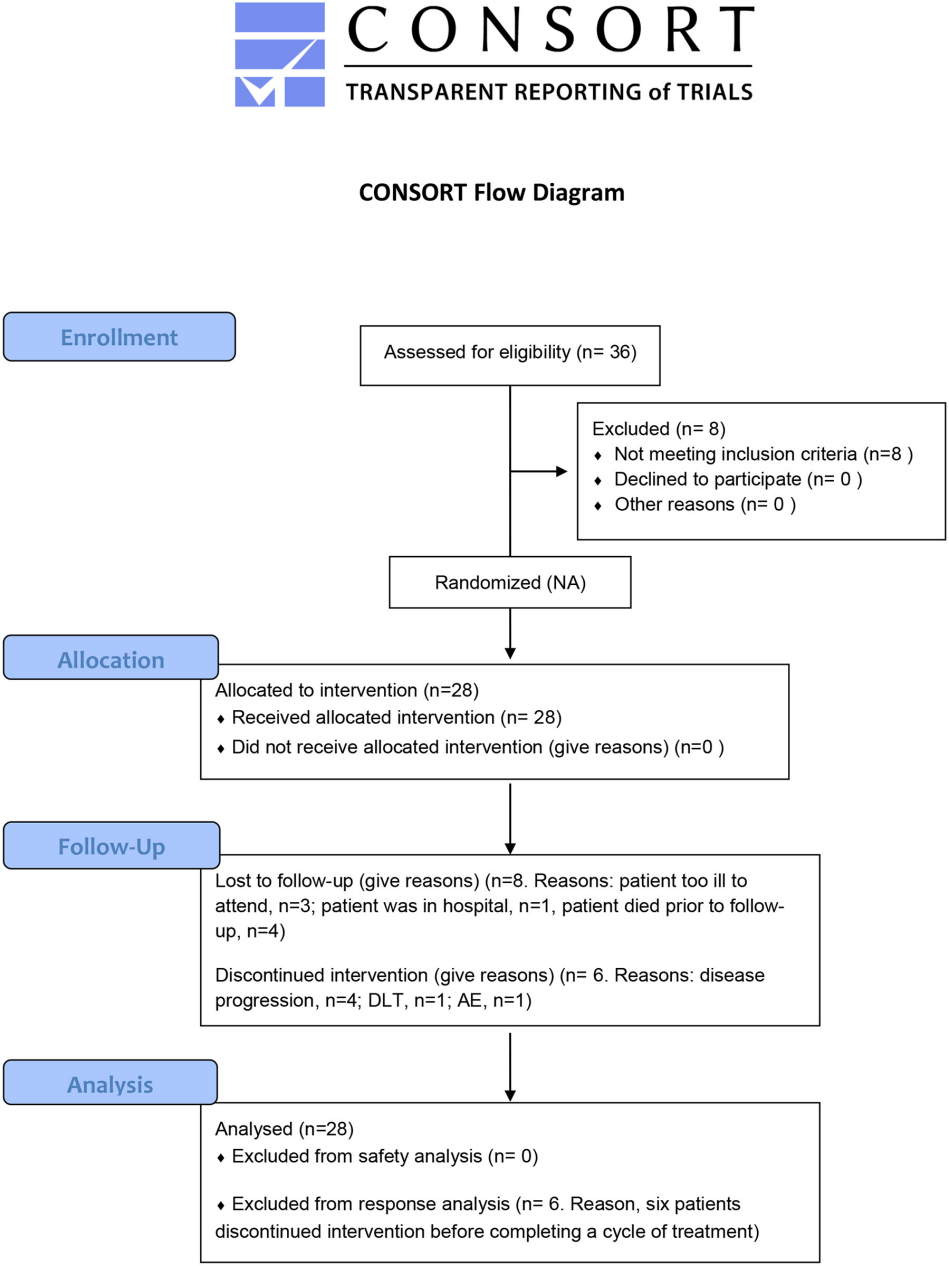
CONSORT Flow diagram. Flow diagram of participants through each stage of the study.

Twenty eight patients with advanced solid tumors were enrolled between August 2009 and September 2011. A flow diagram of the progress through the phases of enrollment, allocation, follow-up, and data analysis of the clinical study is shown in Fig 1. Baseline characteristics of the patients are listed in Table 1. Patients were treated with one of the seven ^EGFR^minicells_Pac_ dose levels as indicated in Table 2. Twenty two patients (79%) completed at least one cycle (five doses; once weekly) of ^EGFR^minicells_Pac_ treatment. The remaining 6 patients (21%) ceased treatment due to rapidly progressive disease in the first cycle (Table 2).

**Table 1:** Baseline Patient Demographic and Clinical Characteristics. Abbreviations: ECOG, Eastern Cooperative Oncology Group.

	Patient Cohort (N = 28)	
	Number	%
Sex		
Male	21	75
Female	7	25
Age, years		
Median	61	
Range	43	
ECOG performance score		
0	7	25
1	21	75
Primary tumor type		
Adenocarcinoma		
Colorectal	7	25
Stomach	4	14
Gastro-Esophageal	2	7
Pancreas	2	7
Bladder	1	4
Left Adrenal	1	4
Nasopharyngeal	1	4
Parotid Gland	1	4
Cutaneous Melanoma	1	4
Neuroendocrine tumor of pancreas	1	4
Non-Small Cell Lung Cancer	1	4
Squamous Cell Carcinoma		
Anus	1	4
Left Aryepiglottic Fold	1	4
Skin	1	4
Soft Palate	1	4
Transitional Cell Carcinoma of the Bladder	2	7
Previous lines of systemic treatment		
0	1	4
1	6	22
2	6	22
≥3	15	54

**Table 2:** ^EGFR^minicells_Pac_ first-time-in-human, phase I trial: dosing and response summary (safety population; N = 28). Abbreviations: ALT, alanine transaminase; AST, aspartate aminotransferase; NA, patient did not complete Cycle 1 of treatment, therefore disease response at the completion of Cycle 1 (5 doses) is not available; SD, stable disease; PD, progressive disease.

Dose Level Cohort	Patient	Dose Level Administered (^EGFR^minicells_Pac_)	No. Doses Received	Dose Limiting Toxicity	Status^a^	Response^b^
1	0101	1x10^8^	2		Withdrawn	NA
	0102	1x10^8^→5x10^7^	5	Hypophosphatemia	Completed	PD
	0103	1x10^8^→5x10^7^	45	Elevated ALT and AST	Completed	SD
	0104	1x10^8^	10		Completed	SD
	0105	1x10^8^	5		Completed	PD
	0106	1x10^8^	15		Completed	SD
2	0201	1x10^9^	5		Completed	PD
	0202	1x10^9^	2	Reactive Arthritis	Withdrawn	NA
	0203	1x10^9^	5		Completed	PD
	0204	1x10^9^	5		Completed	PD
	0205	1x10^9^	9		Completed	SD
	0206	1x10^9^	5		Completed	PD
3	0301	3x10^9^	10		Completed	SD
	0302	3x10^9^	1		Withdrawn	NA
	0303	3x10^9^	5		Completed	PD
	0304	3x10^9^	4		Withdrawn	NA
4	0401	1x10^10^	5		Completed	PD
	0402	1x10^10^	15		Completed	SD
	0403	1x10^10^	25		Completed	SD
	0404	1x10^10^	1		Withdrawn	NA
	0405	1x10^10^	4		Completed	PD
	0406	1x10^10^	5		Completed	PD
5	0501	5x10^10^→1x10^10^	10		Completed	SD
	0502	5x10^10^→1x10^10^	5		Completed	PD
6	0601	2x10^10^→1x10^10^	19		Completed	SD
7	0701	1.5x10^10^	1		Withdrawn	NA
	0702	1.5x10^10^→5x10^9^	8	Hypotension	Completed	SD
	0703	1.5x10^10^→1x10^10^	5		Completed	PD

^a^Status: withdrawn = patient withdrew prior to the end of cycle; completed = patient withdrew at completion of cycle.

^b^Disease Response in patients at the end of Cycle 1 for individuals that completed Cycle 1 of treatment.

### Dose Limiting Toxicities

This study was an open-label, multi-center, phase I clinical study. The dose escalation component used a 3 + 3 design in which patients were enrolled sequentially on 1 of 7 dose levels. Initially, 6 dose levels were planned, with 3–6 subjects per dose level. Due to safety committee recommendations, the study drug doses were revised and a 7^th^ dose level was added (Table 2).

Two patients in cohort 1 (1x10^8^) experienced dose limiting toxicities (DLT) (Grade 3 hypophosphatemia, n = 1; Grade 3 elevation in alanine transaminase (ALT) and aspartate aminotransferase (AST), n = 1). The ongoing patients’ doses were reduced to 5x10^7 EGFR^minicells_Pac_, with one patient receiving a total of 45 doses over the study duration. A further 3 patients received 1x10^8 EGFR^minicells_Pac_, with no Grade 3 drug-related adverse events (AE).

The dose was escalated to dose cohort 2 (1x10^9^); one patient experienced severe joint pain accompanied by a significant rise in IFNα, and was subsequently diagnosed with a seronegative reactive arthritis (Table 2). This event was considered a DLT and the cohort was also extended to 6 patients. No further DLTs were reported at this dose level and no other patients on the trial experienced similar events. No patient in dose cohort 3 (3x10^9^) or cohort 4 (1x10^10^) experienced DLTs. Safety data supported escalating the dose.

The 2 patients in cohort 5 (5x10^10^) experienced a Grade 3–4 increase in ALT and AST. These changes were transient and as such did not meet the protocol’s amended definition of a DLT. However, as the patients experienced fever, rigors, and nausea, the decision was made to de-escalate to a lower intermediate dose level. Dose cohort 6 (2x10^10^) was deemed unsafe after a single patient was treated and similarly experienced a Grade 3–4 transient elevation in ALT and AST. Three patients were recruited to cohort 7 (1·5x10^10^) which was found to be unsafe due to a DLT (Grade 3 hypotension, n = 1) and an AE (Grade 3 AST increase, n = 1).

The dose of 1x10^10^ (cohort 4) was explored further, with an additional 3 patients recruited to this dose level. One patient was withdrawn from the study due to a disease-related serious AE (supraclavicular mass pressing on the brachiocephalic vein). The remaining 2 patients completed the first cycle of treatment.

Significant toxicity was observed at dose levels administered in excess of 1 x 10^10 EGFR^minicells_Pac_ (1.5 x 10^10^, 2 x 10^10^, and 5 x 10^10^), in particular prolonged fever and transient elevations in liver function tests. The dose of 1x10^10^ was considered deliverable and this was therefore considered to be the maximum tolerated dose (MTD).

### Adverse Events

The most common treatment-related AEs were transient chills (n = 16, 57%) and pyrexia (n = 13, 46%). Seven patients (25.0%) experienced an increase in AST, 4 (14%) experienced an increase in ALT, 3 (11%) experienced nausea, and 3 (11%) patients experienced hypophosphatemia. All AEs reported where a relationship to the study medication was considered possible, probable or definite have been listed in S1 Table. The majority of treatment-related AEs according to the Common Toxicity Criteria for AEs (CTCAE) were moderate (Table 3), with 4 individuals experiencing life threatening (Grade 4) treatment-related AEs (Table 3). For 2 of the Grade 4 events (lymphopenia, n = 2) no action was required and both patients recovered within 24 hours (h). The remaining Grade 4 events included elevated AST (n = 1) and elevated ALT (n = 1), for both of these events the patients’ dose level was reduced, both patients recovered and continued with treatment. Treatment-related serious AEs (SAE) were reported in 5 patients; 3 probably related (reactive arthritis n = 1, post infusion reaction n = 1, fevers n = 1) and 2 definitely related (elevated liver function tests n = 1, hypotension n = 1). No deaths resulted from treatment-related AEs.

**Table 3:** Adverse events by Grade, with a possible, probable or definite relationship to ^EGFR^minicells_Pac_ (N = 28). Abbreviation: AE, Adverse Event.

**Patient Based Summary**	**Dose Level Cohort**							
	**Level 1 1x10** ^**8**^ **N = 6**	**Level 2 1x10** ^**9**^ **N = 6**	**Level 3 3x10** ^**9**^ **N = 4**	**Level 4 1x10** ^**10**^ **N = 6**	**Level 5 5x10** ^**10**^ **N = 2**	**Level 6 2x10** ^**10**^ **N = 1**	**Level 7 1.5 x 10** ^**10**^ **N = 3m**	**Overall N = 28**
**Toxicity**	**n (%)**	**n (%)**	**n (%)**	**n (%)**	**n (%)**	**n (%)**	**n (%)**	**n (%)**
Number of patients reporting treatment-related AEs	3(50)	6(100)	3(75)	6(100)	2(100)	1(100)	3(100)	24(86)
**Number of patients reporting treatment-related AEs by severity** ^a^:								
Grade 1	1(17)	4(67)	1(25)	5(83)	2(100)	1(100)	3(100)	17(61)
Grade 2	2(33)	5(83)	3(75)	4(67)	2(100)	1(100)	3(100)	20(71)
Grade 3	2(33)	1(17)	0	2(33)	2(100)	1(100)	2(67)	10(36)
Grade 4	0	1(17)	1(25)	0	1(50)	1(100)	0	4(14)
Resulting in death	0	0	0	0	0	0	0	0
**Event Based Summary**	**Dose Level Cohort**							
**Toxicity**	**Level 11x10** ^**8**^ **N = 6**	**Level 2 1x10** ^**9**^ **N = 6**	**Level 3 3x10** ^**9**^ **N = 4**	**Level 4 1x10** ^**10**^ **N = 6**	**Level 5 5x10** ^**10**^ **N = 2**	**Level 6 2x10** ^**10**^ **N = 1**	**Level 7 1.5 x 10** ^**10**^ **N = 3**	**Overall N = 28**
Number of treatment-related AEs	10	21	8	34	11	53	36	173
**Number of treatment-related AEs by severity** ^a^:								
Grade 1	1	12	2	16	4	30	14	79
Grade 2	4	7	5	14	3	15	19	67
Grade 3	5	1	0	4	3	7	3	23
Grade 4	0	1	1	0	1	1	0	4
Resulting in death	0	0	0	0	0	0	0	0

^a^Severity is according to the Common Terminology Criteria for Adverse Events (CTCAE). Grade 1 = Mild, Grade 2 = Moderate, Grade 3 = Severe, Grade 4 = Life-threatening, Grade 5 = Resulting Death.

### Clinical Laboratory Evaluations

**Fig 2:**
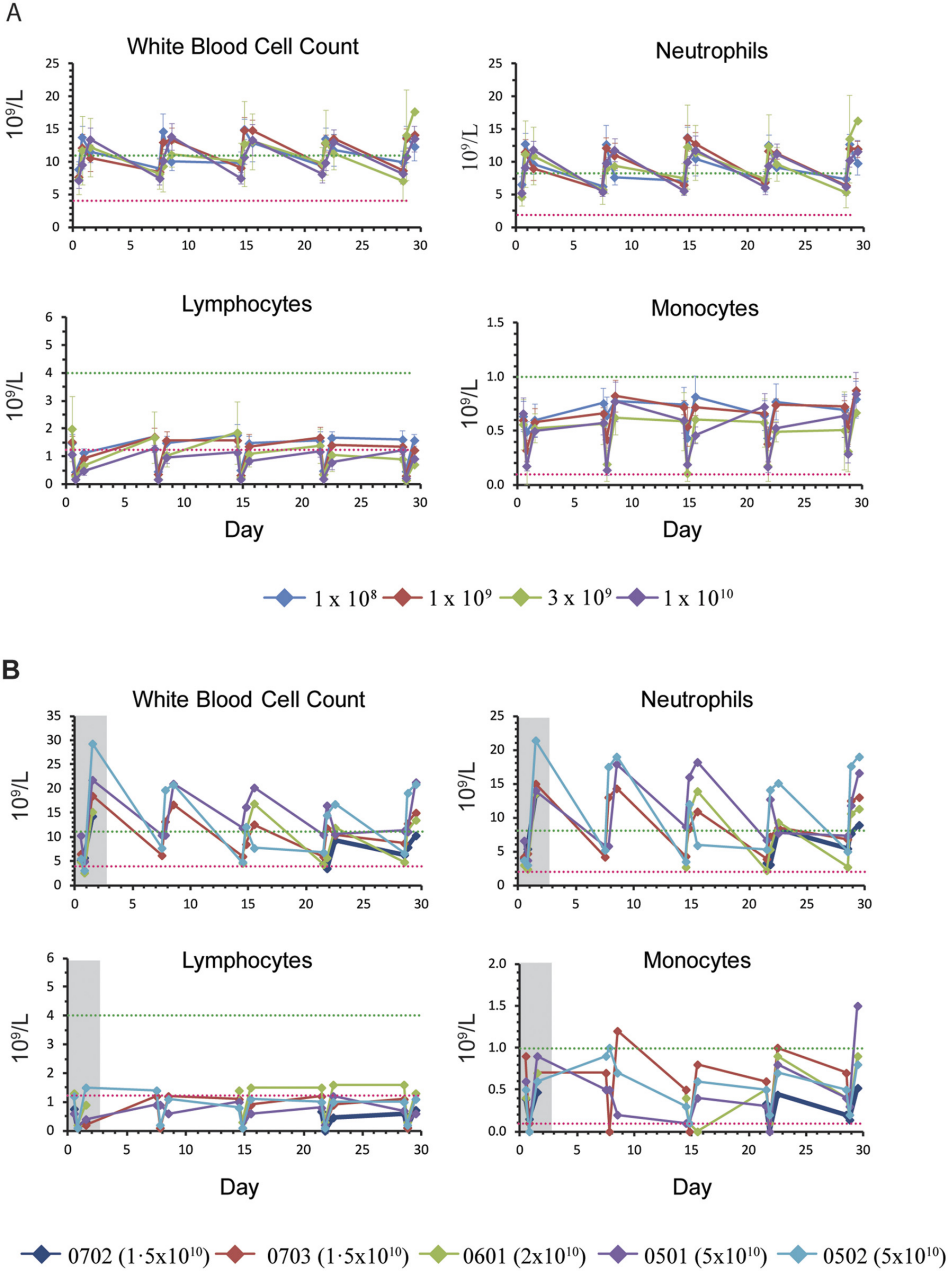
Hematology parameters. Mild elevations in white blood cells and neutrophils, and drops in lymphocytes and monocytes were observed post dose. (A) The mean values (1x10^8^, n = 5; 1x10^9^, n = 5; 3x10^9^, n = 4; 1x10^10^, n = 5) are shown for each dose level up to and including the MTD for Cycle 1 of treatment at pre-dose, 4h and 24h post-dose. Dotted lines indicate normal ranges, error bars indicate the standard error of the mean. (B) Values for Cycle 1 of treatment at pre-dose, 4h and 24h post-dose for 5 individuals who received a treatment dose above the MTD. Grey box indicates samples collected from dose levels above the MTD. Dotted lines indicate normal ranges.

Hematological parameters were transiently affected at doses up to the MTD (1x10^10^). Most patients experienced a mild elevation of their white blood cell count and neutrophils at 4h post-dose, and drops in lymphocytes and monocytes which returned to baseline by the next dose (Fig 2A). These abnormalities were amplified at doses above the MTD (Fig 2B). At doses above the MTD most patients incurred a significant elevation in liver enzymes (S2 and S3 Figs).

**Fig 3:**
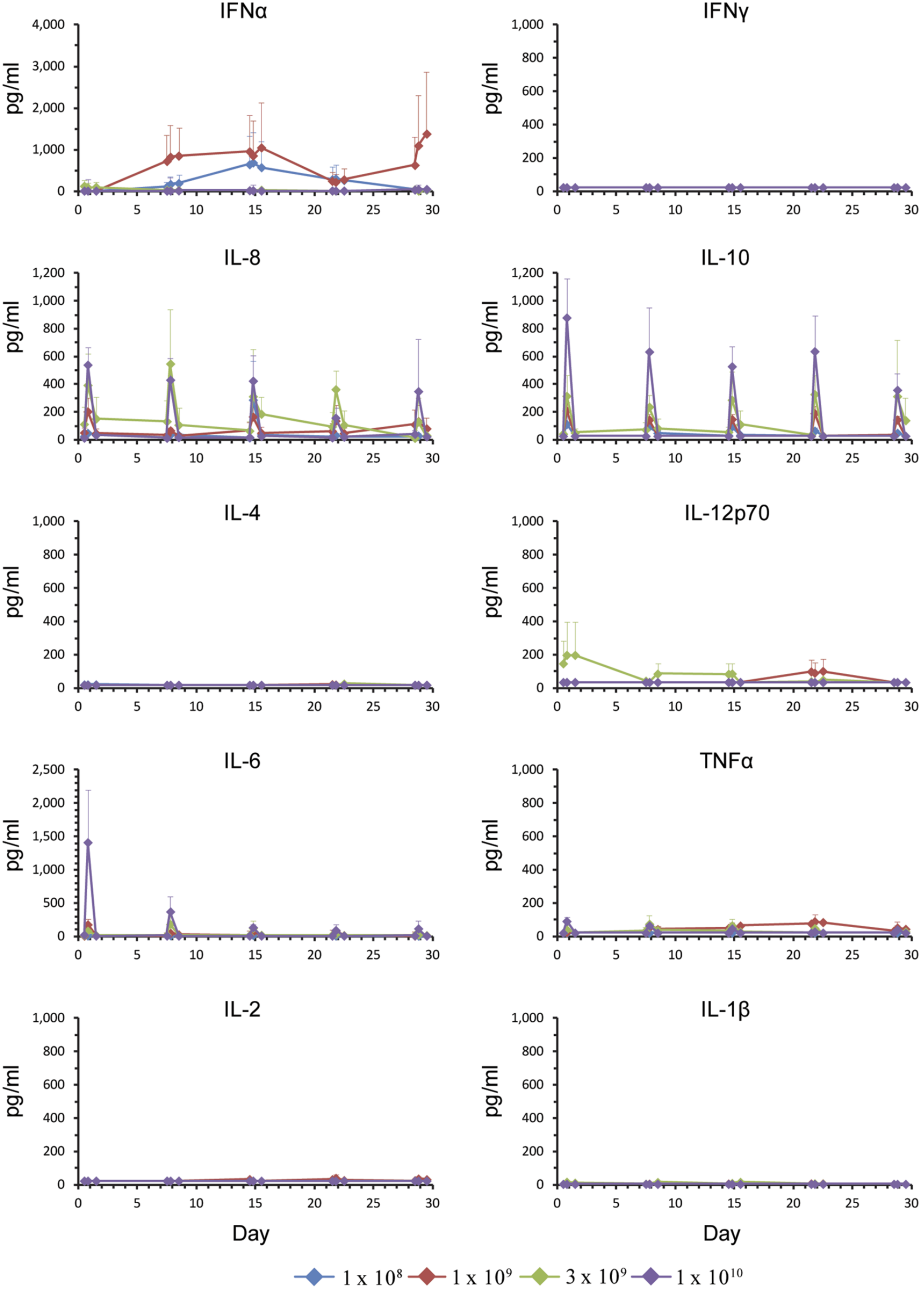
Cytokine response. At 4h post-dose, IL-6, IL-8, and IL-10 spiked and returned to normal by 24h post-dose. IFNα was elevated in certain patients for the duration of the study. The mean values (1x10^8^, n = 5; 1x10^9^, n = 5; 3x10^9^, n = 4; 1x10^10^, n = 5) are shown for each dose level up to and including the MTD for Cycle 1 of treatment at pre-dose, 4h and 24h post-dose.

Serum samples at pre-dose, 4h and 24h post-dose, were analyzed for inflammatory and anti-inflammatory cytokines including TNFα, IL-6, IL1β, IL-2, IL-4, IL-8, IL-12p70, IFNα, IFNγ, and IL-10. At 4h post-dose, IL-6, IL-8 and IL-10 spiked and returned to normal by 24h post-dose (Fig 3). The responses appeared to be dose-dependent, but were not augmented upon repeat dosing (Fig 3 and S4 Fig). Levels of IFNα were elevated in certain patients for the duration of the study, however this was not related to dose level (Fig 3).

Pharmacokinetic (PK) assessment of serum paclitaxel levels was not performed as it was determined that the maximum possible concentration of paclitaxel administered in a single dose of ^EGFR^minicells_Pac_ would be 4 orders of magnitude below (or 10,000-fold less than) technically feasible levels of detection [9].

### Immunogenicity

**Fig 4:**
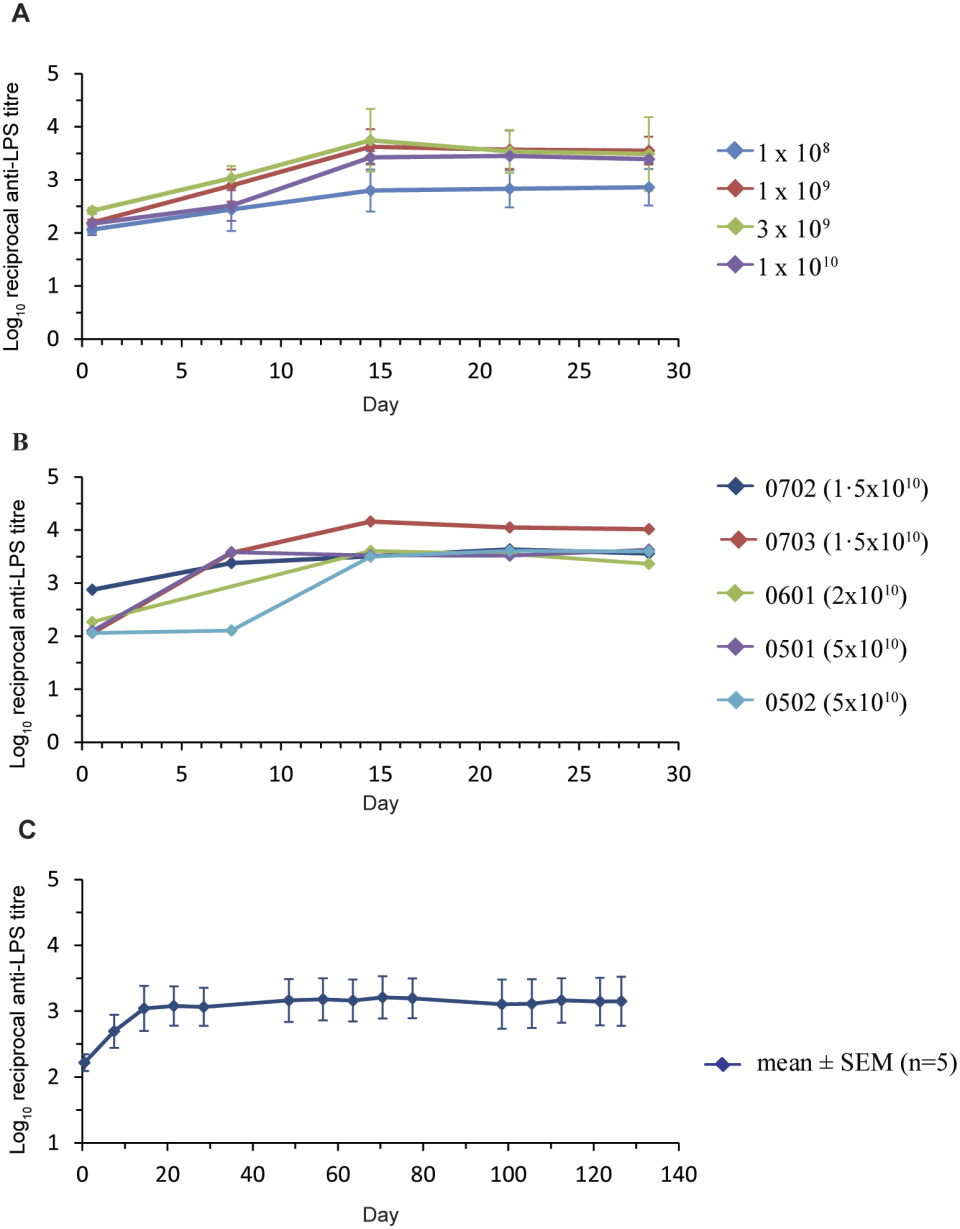
Anti-LPS antibody titers following administration of ^EGFR^minicells_Pac_. Blood samples were collected at dosing and serum was analyzed for anti-LPS IgG. Results are expressed as the reciprocal antibody titer, expressed as Log10. The x-axis shows days of treatment and error bars indicate the standard error of the mean. (A) The mean anti-LPS antibody titer for each dose level (1x10^8^, n = 5; 1x10^9^, n = 5; 3x10^9^, n = 4; 1x10^10^, n = 5) up to and including the MTD for Cycle 1 of treatment (5 doses). (B) Anti-LPS antibody responses in 5 individuals treated with ^EGFR^minicells_Pac_ at dose levels above the MTD. (C) Anti-LPS antibody responses in 5 individuals that received at least 15 doses of ^EGFR^minicells_Pac_.

Antibodies to *Salmonella typhimurium* (anti-LPS) and cetuximab at screening were negative in all patients. All patients, with the exception of one, developed positive *S*.*typhimurium* antibody titers following treatment with the ^EGFR^minicells_Pac_ (27/28 = 96%). Anti-LPS antibody titers reached a peak by dose 3 (Day 15) and were maintained at that level despite repeat dosing (Fig 4). The increase in antibody titer ranged from 2 to 134-fold over baseline (average 32-fold). Average antibody titers for Cycle 1 of treatment are shown in Fig 4A. No patients developed positive cetuximab antibody titers.

### Antitumor Activity

Tumor evaluation was conducted at baseline and after every cycle (6 week intervals), or at the end of treatment/discontinuation. Overall, 26 of the 28 patients were evaluated for response; 2 patients withdrew for reasons other than progressive disease and were therefore not evaluable for response. Of the 26 patients (92.8%) evaluated, 22 (78.6%) completed cycle 1, and of these, 10 patients (45%) demonstrated stable disease, and 12 (55%) had progressive disease.

## Discussion

This first-time-in-human Phase I dose escalation trial was the first study of BsAb-targeted, cytotoxic drug-packaged minicells in humans. The primary objective was to evaluate the safety, tolerability, and MTD of EGFR-targeted minicells loaded with the chemotherapeutic paclitaxel. Our findings show that ^EGFR^minicells_Pac_ can be safely administered at a MTD of 1x10^10 EGFR^minicells_Pac_ per dose. The MTD corresponds to a dose of cetuximab at 0.001% of the usual dose of 250 mg/m^2^, and paclitaxel at 0·0015% of the usual dose of 175 mg/m^2^. Five patients who received a dose higher than the MTD showed a transient elevation in liver enzymes suggesting that doses higher than 1x10^10 EGFR^minicells_Pac_ may cause liver toxicity. No further elevations in liver function tests were seen, in these same patients, when the dose was lowered to 1x10^10 EGFR^minicells_Pac_. Importantly, minicells were safe and generally well tolerated with no treatment-related deaths.

The most common AEs experienced during ^EGFR^minicells_Pac_ treatment were transient and self-limiting fevers and rigors. Where an individual experienced fever or rigors this coincided with a rise in blood pressure, but typically resolved within an hour of onset and was predominantly experienced in the first dose of minicell administration. Four patients (14%) experienced a DLT during the study. One was reactive arthritis, one was hypotension, one experienced symptomless hypophosphatemia, and another experienced symptomless elevated ALT and AST all of which resolved spontaneously. The protocol was amended to exclude these events from the definition of DLTs for future cohorts. Four of the six patients who received doses exceeding the MTD also displayed severe (Grade 3–4), though asymptomatic, elevations in liver enzymes (AST, ALT). These, although not defined as DLTs in the amended protocol, were considered dose-limiting as they were accompanied by other AEs including fever, rigors and nausea.

Prior to this study, the vast body of literature on bacterial endotoxins (LPS) suggested that with over 2 million LPS molecules per bacterial cell, the administration of 10^9^ or more minicells IV in humans would result in severe septic shock. Our data suggests that when LPS is membrane anchored in an intact bacterially derived minicell, with the lipid A (endotoxic part) buried in the membrane, it is safely tolerated despite repeat IV dosing (15 to 45 doses administered in several patients with no severe adverse events). This data, for the first time, sheds new light on our understanding of the long infamous LPS component that is responsible for endotoxic shock associated with Gram-negative bacterial septicemia and on what to expect when such minicells are administered IV in humans. Prior literature shows that pyrogenic reactions and shock are induced in mammals upon IV injection of LPS at low concentrations (1 ng/mL) [10]. The maximum level of endotoxin for IV applications of pharmaceutical and biologic product is set to 5 endotoxin units (EU) per kg of body weight per hour (equating to ~300 EU in an average patient of 60 kg) by all pharmacopoeias. In contrast, each minicell dose administered in the patients in this trial resulted in an IV injection of ~ 43,000 EU anchored to the EDV membrane. This study sheds new light on the biology of LPS and indicates that LPS may be safely administered in patients when the LPS, of the therapeutic, is membrane anchored.

Minicells can be readily packaged with a range of chemotherapeutics and molecularly targeted drugs (reviewed in [11]). Despite the putative advantages of drug loaded and targeted minicells, systemic administration of a bacterially derived product can elicit potent inflammatory responses by Toll-like receptors [12] resulting in the release of cytokines such as IL-1, IL-6 and TNFα. In this study, patients showed a transient elevation in TNFα and IL-6 within 4h post-dose which was associated with occasional rigors, although a simultaneous spike in IL-10 which is known to suppress the pyrogenicity of IL-6 [13, 14] was also observed. With subsequent doses of ^EGFR^minicells_Pac_ we observed a dramatic reduction in IL-6 and TNFα spikes and a mild reduction in the IL-10 response, suggesting that patients developed a tolerance to treatment.

Immunogenicity was evaluated by examining the serum antibody responses to the O-polysaccharide component of the minicell LPS and to the human monoclonal antibody cetuximab used in the BsAb. Patients became weakly positive for anti-LPS with titers rising to a peak at dose 3 with no further rises observed despite one patient receiving 45 doses. This LPS antibody response is similar to that seen in preclinical dog [1] and monkey studies. This weak immunogenicity, against the minicell, suggests that ^EGFR^minicells_Pac_ may have limited immune cell recognition thus allowing repeat dosing without affecting the effectiveness of subsequent doses.

The minicell membrane is a rigid and stable biological membrane, where unlike in liposomes, the drug payload does not leak into the extracellular environment or in serum to cause non-target cell damage. Paclitaxel is associated with a number of toxicities such as hypersensitivity reactions, neutropenia, alopecia and nausea. The dose of paclitaxel administered in 1x10^10 EGFR^minicells_Pac_ equates to ~ 500 ng (0·0015% of 175 mg/m^2^), and a significant reduction in paclitaxel toxicity was observed. The monoclonal antibody, cetuximab, is an EGFR inhibitor and, due to the ubiquitous expression of EGFR receptors, severe skin toxicities are observed with its use. No skin toxicities were observed with ^EGFR^minicells_Pac_ treatment. This lack of toxicity may stem from the fact that IV administered minicells are retained within the circulatory system due to their very large size and do not extravasate into non-target tissue where the fenestrations are less than 100 nm [15]. However, the fenestrations in the leaky vasculature surrounding a tumor are known to range between 200 nm to more than 1 μm [16–18] and the minicells are likely to passively target the tumor and be retained in the tumor microenvironment by the EPR effect.

Ten patients achieved stable disease on restaging at 6 weeks and went on to receive further cycles of therapy. No objective responses were seen and this most likely reflects the limited activity of paclitaxel in this patient population where patients have been pretreated with multiple lines of chemotherapy and drug resistance has developed. Secondly, since EGFR expression was not confirmed, there may have been insufficient EGFR expression on the treated tumors to allow minicell uptake. Assessment of EGFR expression was not performed in this first-time-in-human study where the primary objective was to determine the safety and maximally tolerated dose of the EGFR-targeted minicells, as it was considered unethical to subject end-stage patients to additional biopsy sampling for a first-time-in-human study. However, enrichment was performed for tumour types likely to have high EGFR expression. To fully assess the therapeutic activity of ^EGFR^minicells, subsequent studies will be conducted in patients with confirmed EGFR expressing tumors. Similarly, biodistribution of minicells, to confirm selective delivery to tumors in patients, is important and will be addressed in future studies.

In conclusion, we have shown that repeated doses of ^EGFR^minicells_Pac_ can be safely administered to patients with advanced solid tumors. A total of 236 doses were administered in this trial over a total of 42 cycles. A dose of 1x10^10 EGFR^minicells_Pac_ was determined as the MTD. This is the first trial to show the safety of the minicell in humans and underlines the safety, feasibility, and the potential of minicells as a suitable vehicle for targeted anticancer therapy.

## Methods

### Study Design

This was an open label, multi-center, first-time-in-human, dose-escalating Phase I trial at three cancer centers (Peter MacCallum Cancer Centre, Royal Melbourne Hospital, Ludwig Cancer Centre) in Australia. Primary objective was to evaluate the safety, tolerability and MTD of intravenously administered ^EGFR^minicells_Pac_ in patients with advanced epithelial malignancies. Secondary objectives were to (i) determine immune and inflammatory responses to ^EGFR^minicells_Pac_ and (ii) document evidence of anti-tumor activity. This study was carried out in accordance with the Clinical Trial Notification scheme of the Australian Therapeutic Goods Administration. The study was approved by the Peter MacCallum Cancer Centre Human Research Ethics Committee (HREC) who also sought independent expert review for immunology, toxicology and formulation/manufacturing since such a three component therapeutic of biological origin had not been tested in humans before. The trial protocol was independently approved by the HRECs from the other two participating cancer centers. This study was registered with the Australian New Zealand Clinical Trials Registry, number ACTRN12609000672257. The protocol for this study and supporting TREND checklist are available as supporting information; see S1 TREND Checklist and S1 Protocol.

### Patients

Principle investigators recruited suitable patients to the study. Eligibility criteria included patients with advanced solid tumors with histological subtypes likely to express EGFR, age 18 years or older, Eastern Cooperative Oncology Group (ECOG) performance status of 0 or 1 and a life expectancy greater than 3 months, with adequate organ and marrow function. Major exclusion criteria were no previous systemic treatments with taxanes or EGFR inhibitors, for example cetuximab or erlotinib, within 30 days prior to the first dose. Detailed inclusion and exclusion criteria are documented in S1 Protocol. All patients provided written informed consent.

### Study Treatment

Minicells were produced and purified from *Salmonella typhimurium*, as previously described [1]. Patients were treated with paclitaxel-packaged minicells targeted to EGFR using a cetuximab-based BsAb (^EGFR^minicells_Pac_). The concentration of paclitaxel in 1x10^9 EGFR^minicells_Pac_ was ~ 50 ng. Toxicology studies in 36 monkeys demonstrated that dose levels ranging from 1x10^8^ to 1x10^9^ of ^EGFR^minicells_Pac_ do not result in AEs. Therefore, a starting dose of 1x10^8^ was chosen for this first-time-in-human trial.

### Study Procedures

All patients were pre-medicated 30 min before administration of ^EGFR^minicells_pac_ with dexamethasone (8 mg), and Promethazine (25 mg) or Loratidine (20 mg), and Paracetamol (1 gm). Each dose was administered via a peripheral vein catheter in a 20 ml volume given as a 20 min infusion by a healthcare professional.

To establish the MTD of ^EGFR^minicells_Pac_, patients received a cycle of treatment consisting of 5 infusions at weekly intervals followed by a treatment free week (days 1, 8, 15, 22, and 29 every 6 weeks). At the end of each cycle, response assessment with CT scans was performed. Patients with stable or responding disease were administered further cycles of ^EGFR^minicells_Pac_. Seven dose levels were evaluated: 1x10^8^, 1x10^9^, 3x10^9^, 1x10^10^, 1·5x10^10^, 2x10^10^ and 5x10^10^, with 3 patients initially entered at each dose level (up to a maximum of 6 patients).

Three patients were initially entered at each dose level, and if no patient experienced a DLT the dose was escalated to the next level and another 3 patients were treated at the higher dose level. If 1 of the 3 initial patients experienced a DLT the cohort was expanded up to 6 patients. The dose escalation continued until at least 2 patients among a cohort of 3 to 6 experienced a DLT and the prior dose level was considered the MTD. In each instance, the safety committee assessed whether the dose should be escalated to the next level.

### Assessments

Patients were monitored for AEs at 1h, 2h, 4h, 6h and 24h after each dose throughout the first cycle and at 1h, 2h, and 4h in subsequent cycles. All toxicities or AEs were graded according to the Common Toxicity Criteria for AEs (CTCAE) Version 3. DLTs were defined as any of the following events that was possibly, probably, or definitely related to ^EGFR^minicells_Pac_ and which occurred during Cycle 1 and met the following criteria. (A) Clinically significant Grade 3 or 4, non-hematologic toxicity (including allergic reaction) except: (i) nausea and vomiting (Note, any treatment-related Grade 3 or 4 nausea or vomiting that persisted for greater than 7 days was considered a DLT), (ii) fever (in the absence of neutropenia), (iii) asymptomatic hyperglycemia or hyperuricemia, (iv) biochemical abnormalities that resolved to Grade 2 or better in 7 or less days,* (v) clinically significant Grade 3 or 4 biochemical abnormalities that persist for more than seven days. (B) Hematological toxicities, (i) febrile neutropenia (absolute neutrophil count (ANC) < 1x10^9^/L and fever > 38·5°C), (ii) Grade 4 neutropenia (ANC < 0·5x10^9^/L) for 7 or more days, (iii) Grade 3 thrombocytopenia with bleeding or Grade 4 thrombocytopenia for 7 or more days. (*Protocol was amended after enrollment of the first 2 patients to exclude asymptomatic biochemical abnormalities that resolved to Grade 2 or better in 7 days). Resumption of study treatment for patients experiencing DLTs was permitted, contingent on the return of that AE to ≤ Grade 1 and interruption or delay of treatment for ≤ 3 weeks. Resumption of treatment after resolution of a DLT was at the next lower dose level tested (or 50% lower if DLT occurred with the first dose level).

Tumor evaluation using CT scans was conducted at baseline and at the end of every cycle, or at the end of treatment/discontinuation. Response Evaluation Criteria in Solid Tumors (RECIST) were used to evaluate target lesions.

### Clinical Laboratory Studies

Blood samples for assessing immune and cytokine response, pharmacokinetic (PK) analysis, serum biochemistry and hematology, were obtained at pre-dose, 4h post-dose, and 24h post-dose. Serum was analyzed for the inflammatory cytokines TNFα, IL1β, IL-2, IL-4, IL-6, IL-8, IL-12p70, IFNα, IFNγ, and anti-inflammatory cytokine IL-10 using ELISA duoset kits (R&D Systems, USA). To assess immunogenicity, ELISA assays to LPS purified from *Salmonella typhimurium* (Sigma) or cetuximab (Merck), were performed in 96 well plates as previously described [1]. The *S*. *typhimurium* antibody titer was defined as the reciprocal serum dilution that gave a half-maximal optical density reading, and a positive antibody titer was defined as 378 or greater.

### Statistics

The number of patients to be treated in this study was dependent on the tolerability of ^EGFR^minicells_Pac_ and identification of the maximum administered dose. The number of patients per dose level was specified in the protocol according to the dose escalation rules, with 3 patients initially assigned per dose level (up to a maximum of 6 per dose level).

Due to the small patient cohort, findings were to be presented in a descriptive manner and no formal statistical comparisons were to be performed. Continuous data were summarized by the following descriptive statistics: n (number of observations), mean, standard deviation, median, minimum, maximum. Categorical data was summarized by frequencies and percentages. All statistical analyses was performed using Excel 2013. The safety population consisted of all enrolled patients who received at least one dose of study medication. Patients who were removed from the study prior to completing the first cycle, for reasons other than DLT, were to be replaced.

## Supporting Information

S1 Table Adverse events with a possible, probable or definite relationship to ^EGFR^minicells_Pac._
(PDF)

S1 Fig Schematic of a drug-loaded minicell with attached bispecific antibody. Schematic showing a minicell (large blue sphere) packaged with the chemotherapeutic drug, paclitaxel (chemical compound particles). The minicell is labelled with bispecific antibody (yellow and pink structures) where one arm (yellow end) of the bispecific antibody attaches to the O-polysaccharide of the minicell (green structure) and the other arm (pink end) is available for attachment to the epidermal growth factor receptor on the cancer cell.
(TIF)

S2 Fig Liver function tests. The mean values (1x10^8^, n = 5; 1x10^9^, n = 5; 3x10^9^, n = 4; 1x10^10^, n = 5) are shown for each dose level up to and including the maximum tolerated dose for Cycle 1 of treatment at pre-dose, 4h and 24h post-dose. Dotted lines indicate normal ranges, error bars indicate the SEM.
(TIF)

S3 Fig Liver function tests in patients treated with a dose above the maximum tolerated dose. Values for Cycle 1 of treatment at pre-dose, 4h and 24 h post-dose for 5 individuals who received a treatment dose above the maximum tolerated dose (MTD). Significant elevation in the liver enzymes were observed above the MTD. Grey box indicates samples collected from dose levels above the MTD, dotted lines indicate normal ranges.
(TIF)

S4 Fig Cytokine response. Values for Cycle 1 of treatment at pre-dose, 4h and 24h post-dose for 5 individuals who received a treatment dose above the maximum tolerated dose (MTD). At 4h post-dose, IL-6, IL-8, and IL-10 spiked and returned to normal by 24h post-dose. Grey box indicates samples collected from dose levels above the MTD, dotted lines indicate normal ranges.
(TIF)

S1 Protocol (PDF)

S1 TREND Checklist (PDF)
