# Supplementary material for: A First-Time-In-Human Phase I Clinical Trial of Bispecific Antibody-Targeted, Paclitaxel-Packaged Bacterial Minicells
Source: PLoS One. 2015 Dec 11;10(12):e0144559. doi: 10.1371/journal.pone.0144559 (PMC4699457; doi:10.1371/journal.pone.0144559)
Supplement: S1 Table — (PDF) [file pone.0144559.s001.pdf]

S1 Table. Adverse events with a possible, probable or definite relationship to $^{EGFR}minicells_{Pac}$

**Toxicity**	**Level 1 1 x 10^8 N=6 n (%)**	**Level 2 1 x 10^9 N=6 n (%)**	**Level 3 3 x 10^9 N=4 n (%)**	**Level 4 1 x 10^{10} N=6 n (%)**	**Level 5 5 x 10^{10} N=2 n (%)**	**Level 6 2 x 10^{10} N=1 n (%)**	**Level 7 1.5 x 10^{10} N=3 n (%)**	**Overall N=28 n (%)**
Any	3 (50.0)	6 (100.0)	3 (75.0)	6 (100.0)	2 (100.0)	1 (100.0)	3 (100.0)	24 (85.7)
**Blood and lymphatic system disorders**								
Lymphopenia	0 (0.0)	1 (16.7)	1 (25.0)	0 (0.0)	0 (0.0)	1 (100.0)	0 (0.0)	3 (10.7)
Thrombocytopenia	0 (0.0)	0 (0.0)	0 (0.0)	0 (0.0)	0 (0.0)	1 (100.0)	0 (0.0)	1 (3.6)
**Gastrointestinal disorders**								
Constipation	0 (0.0)	0 (0.0)	0 (0.0)	0 (0.0)	0 (0.0)	1 (100.0)	0 (0.0)	1 (3.6)
Diarrhea	0 (0.0)	0 (0.0)	0 (0.0)	1 (16.7)	0 (0.0)	0 (0.0)	0 (0.0)	1 (3.6)
Nausea	0 (0.0)	1 (16.7)	0 (0.0)	1 (16.7)	0 (0.0)	1 (100.0)	0 (0.0)	3 (10.7)
Paresthesia oral	0 (0.0)	1 (16.7)	0 (0.0)	0 (0.0)	0 (0.0)	0 (0.0)	0 (0.0)	1 (3.6)
Vomiting	0 (0.0)	0 (0.0)	0 (0.0)	1 (16.7)	0 (0.0)	1 (100.0)	0 (0.0)	2 (7.1)
**General disorders and administration site conditions**								
Chills	1 (16.7)	3 (50.0)	3 (75.0)	3 (50.0)	2 (100.0)	1 (100.0)	3 (100.0)	16 (57.1)
Fatigue	0 (0.0)	0 (0.0)	0 (0.0)	0 (0.0)	0 (0.0)	1 (100.0)	2 (66.7)	3 (10.7)
Pyrexia	1 (16.7)	1 (16.7)	2 (25.0)	4 (83.3)	1 (100.0)	1 (100.0)	3 (100.0)	13 (46.4)
**Hepatobiliary disorders**								
Hyperbilirubinemia	0 (0.0)	0 (0.0)	0 (0.0)	0 (0.0)	0 (0.0)	1 (100.0)	0 (0.0)	1 (3.6)
**Immune system disorders**								
Infusion reaction	0 (0.0)	0 (0.0)	0 (0.0)	1 (16.7)	0 (0.0)	0 (0.0)	0 (0.0)	1 (3.6)
Hypersensitivity	0 (0.0)	0 (0.0)	1 (25.0)	0 (0.0)	0 (0.0)	0 (0.0)	0 (0.0)	1 (3.6)
**Infections and infestations**								
Herpes zoster	0 (0.0)	1 (16.7)	0 (0.0)	0 (0.0)	0 (0.0)	0 (0.0)	0 (0.0)	1 (3.6)
**Investigations**								
ALT increased	1 (16.7)	0 (0.0)	0 (0.0)	0 (0.0)	2 (100.0)	1 (100.0)	0 (0.0)	4 (14.3)
AST increased	1 (16.7)	0 (0.0)	0 (0.0)	1 (16.7)	2 (100.0)	1 (100.0)	2 (66.7)	7 (25.0)
ALP increased	0 (0.0)	0 (0.0)	0 (0.0)	0 (0.0)	1 (50.0)	1 (100.0)	0 (0.0)	2 (7.1)
Bilirubin increased	0 (0.0)	0 (0.0)	0 (0.0)	1 (16.7)	0 (0.0)	0 (0.0)	1 (33.3)	2 (7.1)
LDH increased	0 (0.0)	0 (0.0)	0 (0.0)	0 (0.0)	0 (0.0)	1 (100.0)	0 (0.0)	1 (3.6)
GGT increased	0 (0.0)	0 (0.0)	0 (0.0)	0 (0.0)	0 (0.0)	1 (100.0)	0 (0.0)	1 (3.6)
**Metabolism and nutrition disorders**								
Hypokalemia	0 (0.0)	0 (0.0)	0 (0.0)	0 (0.0)	0 (0.0)	1 (100.0)	1 (33.3)	2 (7.1)
Hypomagnesaemia	0 (0.0)	0 (0.0)	0 (0.0)	0 (0.0)	0 (0.0)	1 (100.0)	1 (33.3)	2 (7.1)
Hypophosphatemia	1 (16.7)	0 (0.0)	0 (0.0)	1 (16.7)	0 (0.0)	0 (0.0)	1 (33.3)	3 (10.7)
**Musculoskeletal and connective tissue disorders**								
Arthritis reactive	0 (0.0)	1 (16.7)	0 (0.0)	0 (0.0)	0 (0.0)	0 (0.0)	0 (0.0)	1 (3.6)

Toxicity	Level 1<br>1 x 10^8<br>N=6<br>n (%)	Level 2<br>1 x 10^9<br>N=6<br>n (%)	Level 3<br>3 x 10^9<br>N=4<br>n (%)	Level 4<br>1 x 10^{10}<br>N=6<br>n (%)	Level 5<br>5 x 10^{10}<br>N=2<br>n (%)	Level 6<br>2 x 10^{10}<br>N=1<br>n (%)	Level 7<br>1.5 x 10^{10}<br>N=3<br>n (%)	Overall<br><br>N=28<br>n (%)
Muscular weakness	0 (0.0)	0 (0.0)	0 (0.0)	0 (0.0)	0 (0.0)	0 (0.0)	1 (33.3)	1 (3.6)
Myalgia	0 (0.0)	1 (16.7)	0 (0.0)	0 (0.0)	0 (0.0)	1 (100.0)	0 (0.0)	2 (7.1)
**Nervous system disorders**								
Dysgeusia	0 (0.0)	1 (16.7)	0 (0.0)	0 (0.0)	0 (0.0)	1 (100.0)	0 (0.0)	2 (7.1)
Headache	0 (0.0)	0 (0.0)	0 (0.0)	0 (0.0)	0 (0.0)	1 (100.0)	0 (0.0)	1 (3.6)
Paresthesia	0 (0.0)	1 (16.7)	0 (0.0)	0 (0.0)	0 (0.0)	0 (0.0)	0 (0.0)	1 (3.6)
Syncope	0 (0.0)	0 (0.0)	0 (0.0)	1 (16.7)	0 (0.0)	0 (0.0)	0 (0.0)	1 (3.6)
**Skin and subcutaneous tissue disorders**								
Alopecia	1 (16.7)	0 (0.0)	0 (0.0)	0 (0.0)	0 (0.0)	0 (0.0)	0 (0.0)	1 (3.6)
Hyperhidrosis	0 (0.0)	1 (16.7)	0 (0.0)	0 (0.0)	0 (0.0)	0 (0.0)	0 (0.0)	1 (3.6)
**Vascular disorders**								
Flushing	0 (0.0)	1 (16.7)	0 (0.0)	0 (0.0)	0 (0.0)	0 (0.0)	0 (0.0)	1 (3.6)
Hot flush	0 (0.0)	1 (16.7)	0 (0.0)	0 (0.0)	0 (0.0)	0 (0.0)	0 (0.0)	1 (3.6)
Hypertension	0 (0.0)	0 (0.0)	0 (0.0)	1 (16.7)	0 (0.0)	0 (0.0)	1 (33.3)	2 (7.1)
Hypotension	0 (0.0)	0 (0.0)	0 (0.0)	0 (0.0)	0 (0.0)	0 (0.0)	1 (33.3)	1 (3.6)
