# Supplementary figures and images for: A First-Time-In-Human Phase I Clinical Trial of Bispecific Antibody-Targeted, Paclitaxel-Packaged Bacterial Minicells
Source: PLoS One. 2015 Dec 11;10(12):e0144559. doi: 10.1371/journal.pone.0144559 (PMC4699457; doi:10.1371/journal.pone.0144559)

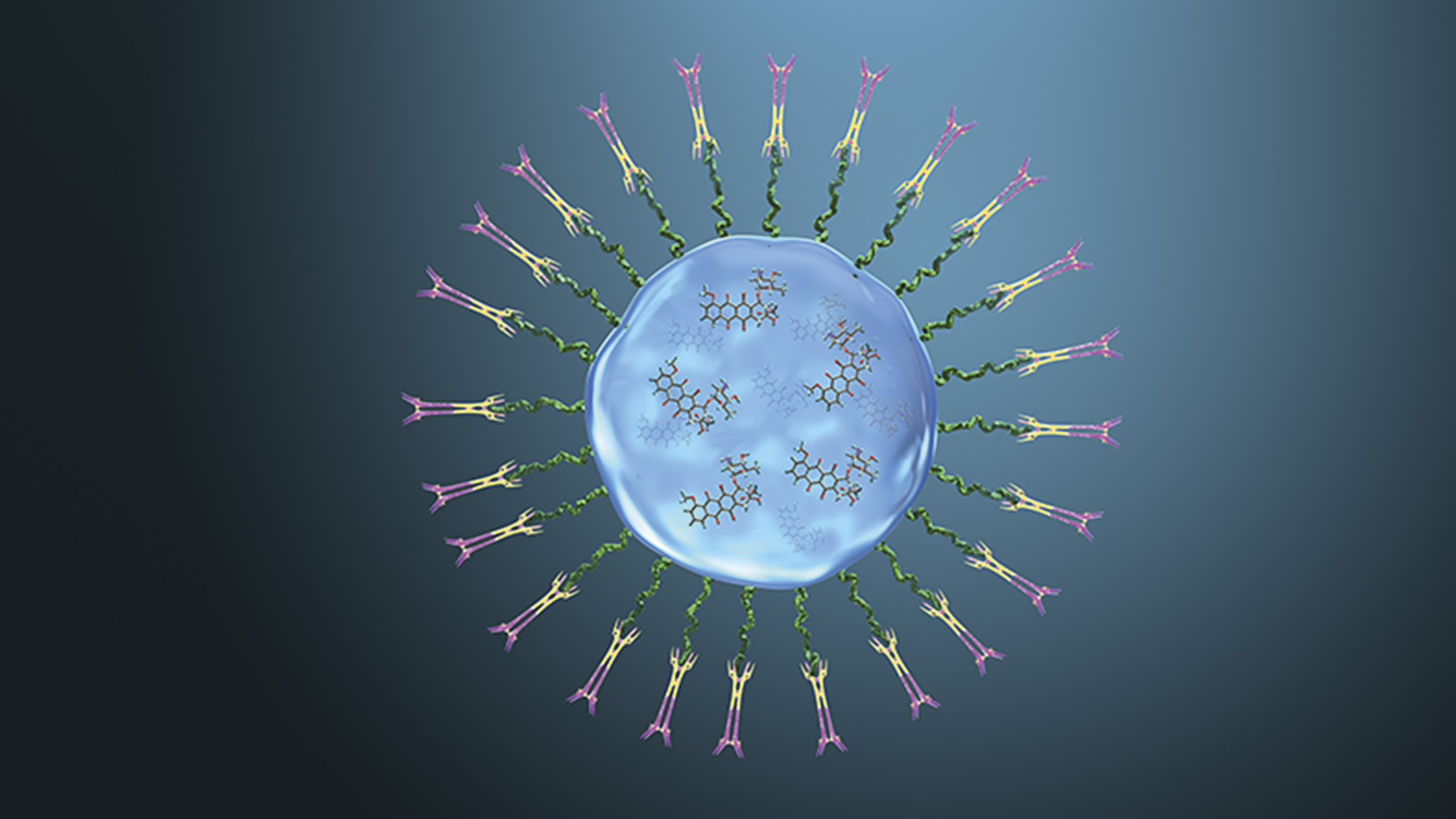

Supplement: S1 Fig — Schematic showing a minicell (large blue sphere) packaged with the chemotherapeutic drug, paclitaxel (chemical compound particles). The minicell is labelled with bispecific antibody (yellow and pink structures) where one arm (yellow end) of the bispecific antibody attaches to the O-polysaccharide of the minicell (green structure) and the other arm (pink end) is available for attachment to the epidermal growth factor receptor on the cancer cell. (TIF) [file pone.0144559.s002.tif]

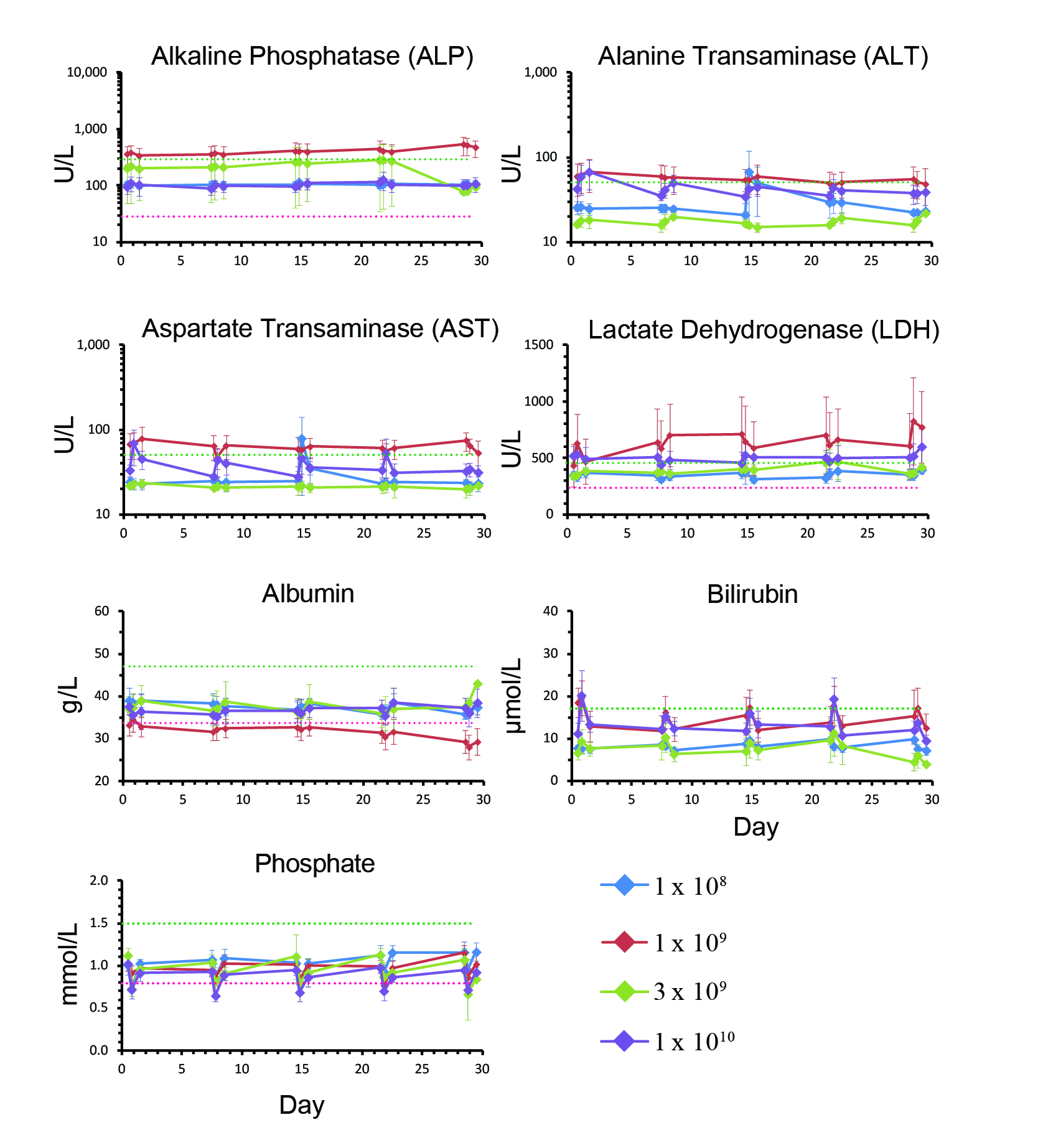

Supplement: S2 Fig — The mean values (1x10^8^, n = 5; 1x10^9^, n = 5; 3x10^9^, n = 4; 1x10^10^, n = 5) are shown for each dose level up to and including the maximum tolerated dose for Cycle 1 of treatment at pre-dose, 4h and 24h post-dose. Dotted lines indicate normal ranges, error bars indicate the SEM. (TIF) [file pone.0144559.s003.tif]

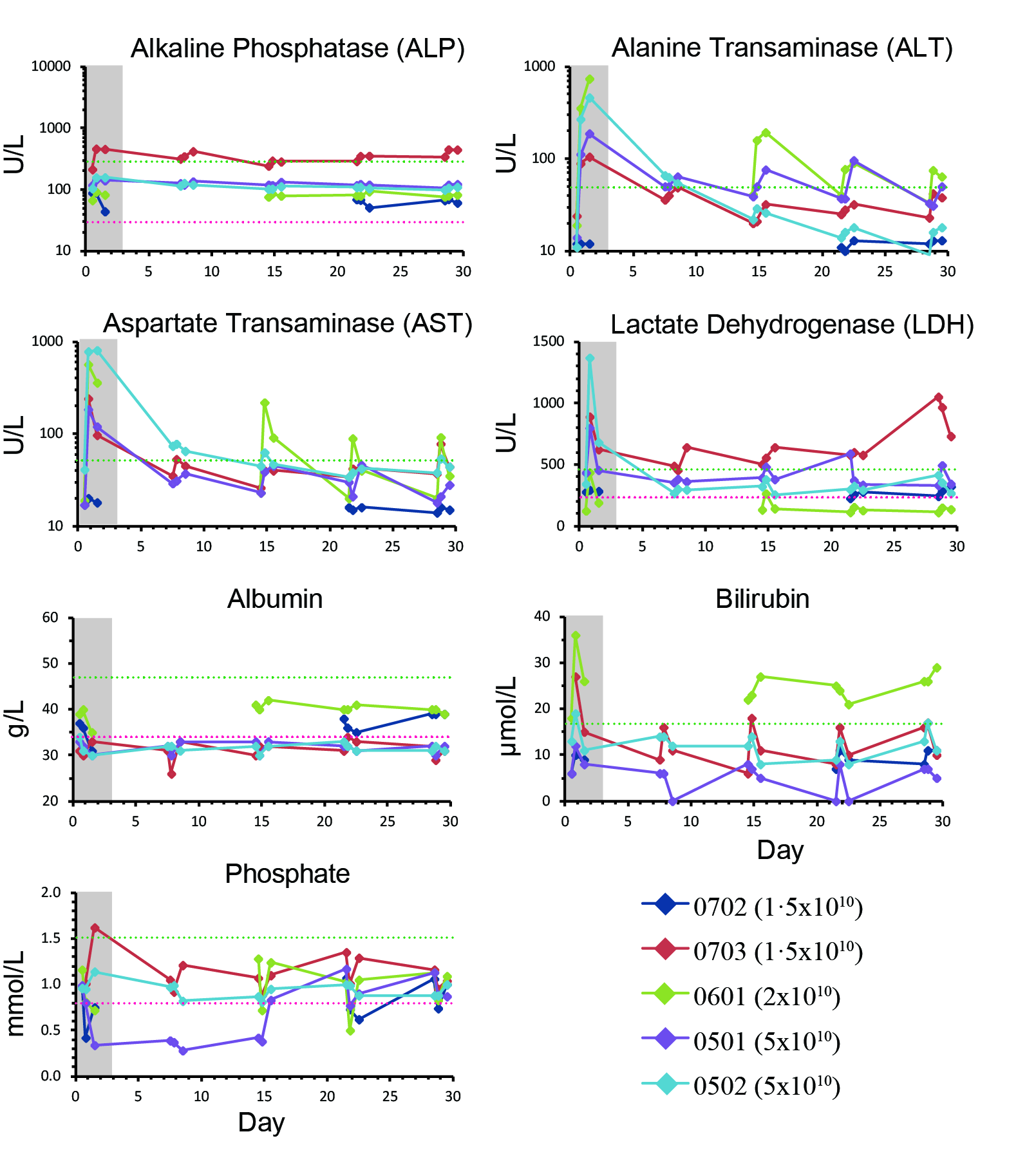

Supplement: S3 Fig — Values for Cycle 1 of treatment at pre-dose, 4h and 24 h post-dose for 5 individuals who received a treatment dose above the maximum tolerated dose (MTD). Significant elevation in the liver enzymes were observed above the MTD. Grey box indicates samples collected from dose levels above the MTD, dotted lines indicate normal ranges. (TIF) [file pone.0144559.s004.tif]

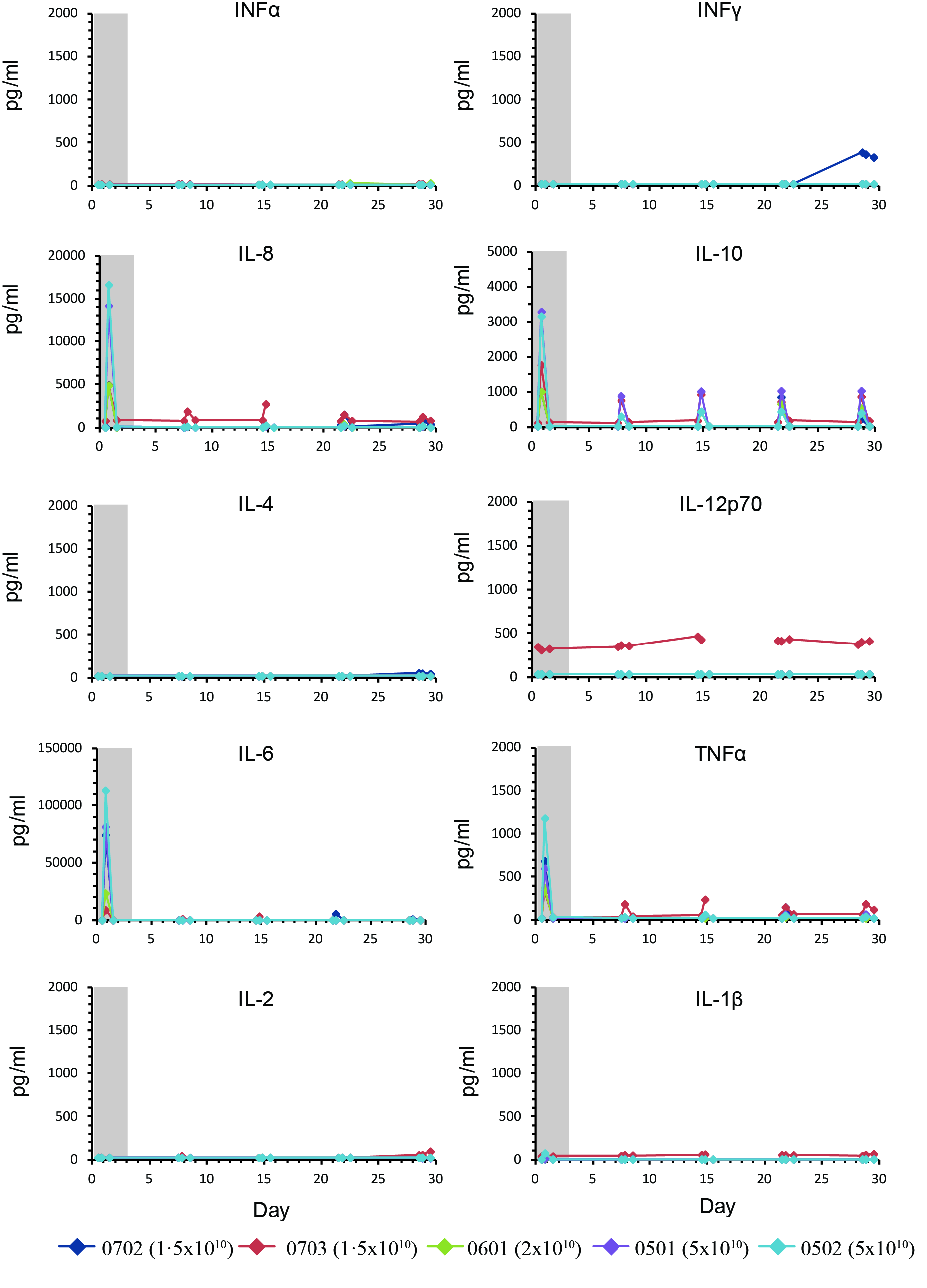

Supplement: S4 Fig — Values for Cycle 1 of treatment at pre-dose, 4h and 24h post-dose for 5 individuals who received a treatment dose above the maximum tolerated dose (MTD). At 4h post-dose, IL-6, IL-8, and IL-10 spiked and returned to normal by 24h post-dose. Grey box indicates samples collected from dose levels above the MTD, dotted lines indicate normal ranges. (TIF) [file pone.0144559.s005.tif]
