# Supplementary material for: A First-Time-In-Human Phase I Clinical Trial of Bispecific Antibody-Targeted, Paclitaxel-Packaged Bacterial Minicells
Source: PLoS One. 2015 Dec 11;10(12):e0144559. doi: 10.1371/journal.pone.0144559 (PMC4699457; doi:10.1371/journal.pone.0144559)
Supplement: S1 Protocol — (PDF) [file pone.0144559.s006.pdf]

CONFIDENTIAL

TITLE: A Phase 1 Study of Epidermal Growth Factor Receptor (EGFR) Targeted, Paclitaxel Loaded EnGeneIC Delivery Vehicles ($^{Erbitux®}$EDVs$_{Pac}$) in Patients with Advanced Solid Tumours

Protocol Number: ENG1

Lead Investigator: **Dr Benjamin Solomon** MBBS, PhD, FRACP
Peter MacCallum Cancer Centre
St Andrew's Place
East Melbourne, Victoria 3002, AUSTRALIA
Telephone: +613 9656 1697 Fax: + 613 9656 1408

Other Principal Investigators:

Professor Andrew Scott
The Ludwig Institute For Cancer Research,
Austin Hospital HSB6,
145-163 Studley Road,
Heidelberg, VIC 3084, AUSTRALIA
Telephone: +613 9496 5876
Fax: +613 9496 5892

Dr Jayesh Desai
The Royal Melbourne Hospital
Grattan St, Parkville, VIC 3050, AUSTRALIA
Telephone: +613 9342 4937
Fax: +613 9342 7508

Co-Investigators:

Associate Professor Grant McArthur
Peter MacCallum Cancer Centre
St Andrew's Place
East Melbourne, Victoria 3002, AUSTRALIA
Telephone: +613 9656 1616
Fax: +613 9656 1408

Professor Mark Rosenthal
Royal Melbourne Hospital
Grattan Street
Parkville, VIC 3050, AUSTRALIA
Telephone: +613 9342 7180
Fax: +613 9342 7508

Study Sponsor: **EnGeneIC Ltd**
Building 2, 25 Sirius Road
Lane Cove West NSW 2066, AUSTRALIA
Telephone: +612 9420 5844 Fax: +612 9420 5877

Sponsor Contacts: **Himanshu Brahmbhatt and Jennifer MacDiarmid**
Joint Managing Directors

Sponsor's Medical Monitor:

Dr Eva Segelov
St Vincent's Hospital
Victoria St
Darlinghurst, NSW 2010 AUSTRALIA
Telephone: +612 8382 2352 Fax: +612 8382 2749

Sponsor's Study Monitor:

Andrew Graham
EnGeneIC Ltd
Building 2, 25 Sirius Road
Lane Cove West NSW 2066, AUSTRALIA
Telephone: +612 9011 6235 ext 108
Mobile: +61 (0)448 832 494

Study Data Management:

Datapharm Australia Pty Ltd
Head Office: 56-56A Thompson Street
DRUMMOYNE NSW 2047
Telephone: +61 2 9719 2800 Fax: +61 2 9719 2811

I have read, and agree to abide by, the attached protocol entitled "A Phase 1 Study of Epidermal Growth Factor Receptor (EGFR) Targeted Paclitaxel Loaded EnGeneIC Delivery Vehicles ($^{Erbitux®}EDVs_{Pac}$) in Patients with Advanced Solid Tumours".

I agree to comply with the National Health and Medical Research Council's National Statement on Ethical Conduct in Human Research (2007) and the Note for Guidance on Good Clinical Practice (CPMP/ICH/135/95) annotated with TGA comments (2000) and other information supplied to me.

I agree to ensure that the confidential information contained in this document will not be used for any purpose other than the evaluation or conduct of the clinical investigation without the prior written consent of EnGeneIC Pty Ltd.

Name (in BLOCK LETTERS)	Signature	Date
Name (in BLOCK LETTERS)	Signature	Date
Name (in BLOCK LETTERS)	Signature	Date

PROTOCOL SYNOPSIS

Title	A Phase 1 Study of Epidermal Growth Factor Receptor (EGFR) Targeted Paclitaxel Loaded EnGeneIC Delivery Vehicles ($^{Erbitux®}$EDVs$_{Pac}$) in Patients with Advanced Solid Tumours.
Study Phase	Phase I
Objective(s)	
Primary:	Evaluate the safety, tolerability and maximal tolerated dose (MTD) of EGFR targeted EDVs carrying paclitaxel ($^{Erbitux®}$EDVs$_{Pac}$) in patients with advanced solid tumours.
Secondary:	1) Determine immune and inflammatory responses to intravenously administered $^{Erbitux®}$EDVs$_{Pac}$.<br>2) Document any evidence of anti-tumour activity using CT and FDG-PET
Study Design	The study is an open label, multi-centre, phase I, clinical trial. There are two parts to the study:<br>In part 1 there will be a dose escalation component in which potentially 6 dose levels will be evaluated: 1 x 10^8 $^{Erbitux®}$EDVs$_{Pac}$, 1 x 10^9 $^{Erbitux®}$EDVs$_{Pac}$, 1 x 10^{10} $^{Erbitux®}$EDVs$_{Pac}$, 5 x 10^{10} $^{Erbitux®}$EDVs$_{Pac}$, 1 x 10^{11} $^{Erbitux®}$EDVs$_{Pac}$ and 2 x 10^{11} $^{Erbitux®}$EDVs$_{Pac}$, with 3 patients entered at each dose level (up to a maximum of 6 patients per dose level). The aim of this part is to determine the maximal tolerated dose (or recommended phase II dose).<br>A cycle of treatment will consist of five infusions of $^{Erbitux®}$EDVs$_{Pac}$ at weekly intervals followed by a treatment free week. At the end of each cycle disease restaging with CT scans will be performed to assess response. Patients with stable or responding disease will be administered further cycles of $^{Erbitux®}$EDVs$_{Pac}$, each cycle involving five infusions of $^{Erbitux®}$EDVs$_{Pac}$ at weekly intervals separated by a treatment free week. Patients with progressive disease may also be eligible to continue to receive treatment if there are no other proven treatment options available to them. Blood samples will be taken for immune response and cytokine measurements after each dose.<br>In part 2, an additional 20 patients with EGFR expressing tumours will be treated at the recommended phase 2 dose determined in part 1. Disease restaging will be performed at the end of each cycle using CT scans. In addition, a FDG PET scan will be performed at baseline and after the first cycle. The aim of this portion of the study is to confirm the safety and tolerability of $^{Erbitux®}$EDVs$_{Pac}$ and to identify preliminary signals of efficacy.

Patients: Patients with advanced solid tumours with histologic subtypes likely to express EGFR and who have not received prior taxane therapy. In part 2, patients will have documented evidence of EGFR expression in tumour biopsies.

Investigational Agent: Anti-EGFR antibody conjugated EDVs loaded with paclitaxel ($^{Erbitux®}EDVs_{Pac}$; also known as EDV1-1)

Route of Administration: Intravenous.

Source of agent: EnGeneIC Pty Ltd

Study Endpoints: The primary endpoint will be toxicity.

The secondary endpoints will be the immune response, cytokine response and tumour response.

Statistical Considerations Descriptive, exploratory data analyses will identify any dose limiting toxicity (DLT) and maximally tolerated dose (MTD) of $^{Erbitux®}EDVs_{Pac}$. Analyses will also describe the observed tumour response, immune and inflammatory responses and presence of paclitaxel in the blood.

Sponsor EnGeneIC Pty Ltd

STUDY SCHEMA

Part 1: Dose escalation

Dose Escalation Schedule	
**Dose Level**	**Dose of $^{Erbitux®}EDVs_{Pac}$**
Level 1	1×10^{8} $^{Erbitux®}EDVs_{Pac}$
Level 2	1×10^{9} $^{Erbitux®}EDVs_{Pac}$
Level 3	1×10^{10} $^{Erbitux®}EDVs_{Pac}$
Level 4	5×10^{10} $^{Erbitux®}EDVs_{Pac}$
Level 5	1×10^{11} $^{Erbitux®}EDVs_{Pac}$
Level 6	2×10^{11} $^{Erbitux®}EDVs_{Pac}$

The animal data with EDVs supports the commencement of dosing at 1×10^{8} $^{Erbitux®}EDVs_{Pac}$. However, in the unlikely event that dose limiting toxicity is seen at this dose level tested, the dose to be evaluated will be reduced to 5×10^{7} $^{Erbitux®}EDVs_{Pac}$ and if necessary reduced again to $1 X 10^{7}$ $^{Erbitux®}EDVs_{Pac}$. The dose increases may be modified if recommended by the Safety Monitoring Committee on review of emerging safety data.

Part 2: Expanded cohort

After completion of the dose escalation (part 1), an additional 20 patients will be enrolled and treated at the recommended phase 2 dose in order to further characterise the safety, toxicity and provide a preliminary indication of efficacy of treatment with $^{Erbitux®}EDVs_{Pac.}$

STUDY DESIGN FLOW CHART

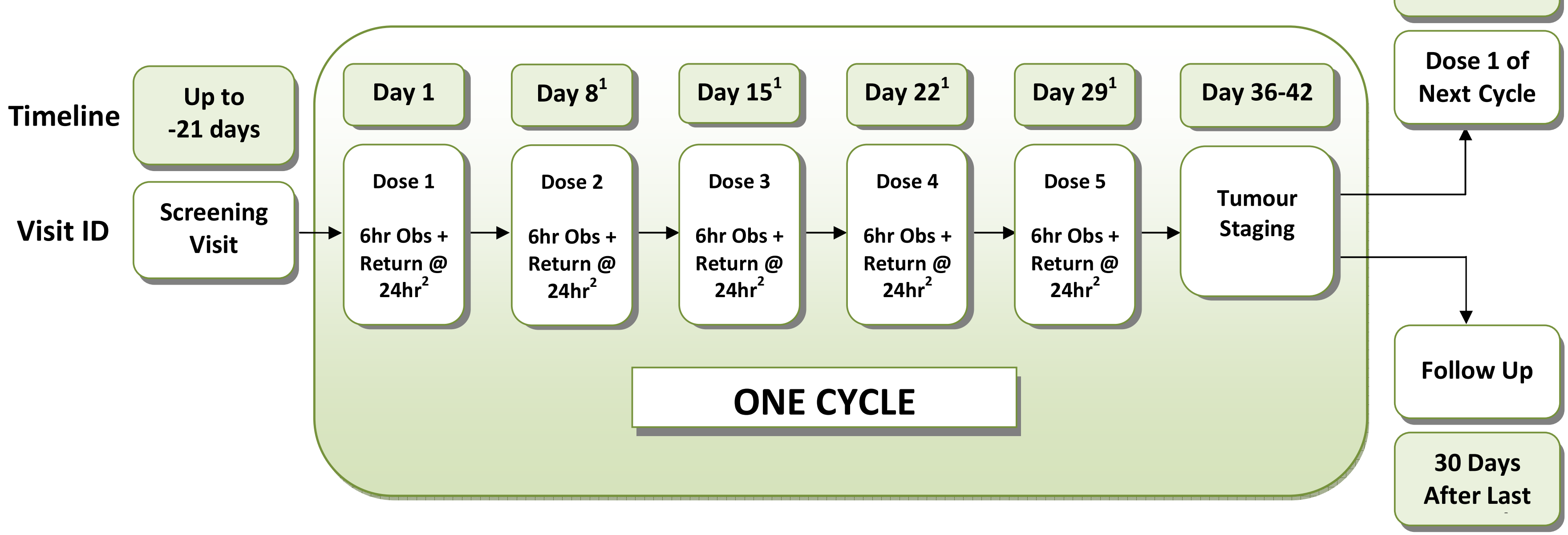

1. Minimum 5 days and maximum 9 days between doses within a cycle. However, as much as possible the gap between doses should be kept to **7** days.
2. **For 1st cycle**: Patients will be observed at clinic for a minimum of 6 hours after dose administration and then return 24 hours after dose administration for a blood sample.
   For 2nd and subsequent cycles: Patients will be observed at the clinic for a minimum of 4 hours after dose administration.
3. Day 1 of subsequent cycles may be delayed by up to 3 weeks if necessary to allow patients to recover from adverse events.
4. If patient discontinues within first cycle, an additional follow-up visit should be conducted within 14 days after the last dose.

GLOSSARY

Abbreviation/ Acronym	Definition
AE	Adverse Event
ALT	Alanine Aminotransferase
ANC	Absolute Neutrophil Count
AST	Aspartate Aminotransferase
BsAb	Bispecific Antibody
C_{min}	Minimum Observed Concentration
C_{max}	Maximum Observed Concentration
CMV	Cytomegalovirus
CR	Complete Response
CRC	Colorectal Carcinoma
CRF	Case Record Form
CRP	C-Reactive Protein
CT	Computed Tomography
CTCAE	Common Terminology Criteria for Adverse Events
Cycle	5 week cycle of treatment consisting of 5 infusions of study treatment at weekly intervals and includes the week following the final dose
DLT	Dose Limiting Toxicity
Dox	Doxorubicin
ECOG	Eastern Cooperative Oncology Group
EDV1-1	$^{Erbitux®}EDVs_{Pac}$
EDVs	EnGeneIC Delivery Vehicle – bacterial minicells which are anucleate nanoparticles
EGFR	Epidermal Growth Factor Receptor
$^{EGFR}EDVs_{Pac}$	EGFR targeted, Paclitaxel loaded EDVs (similar convention used for EDVs targeted with other antibodies and loaded with other cytotoxics)
$^{Erbitux®}EDVs_{Pac}$	EGFR targeted using cetuximab (Erbitux®), Paclitaxel loaded EDVs
FDA	Food and Drug Administration
FDG-PET	^{18}Fluorodeoxyglucose Positron Emission Tomography
GCP	Good Clinical Practice
HCG	Human Chorionic Gonadotrophin
HIV	Human Immunodeficiency Virus
HREC	Human Research Ethics Committee
ICH	International Conference on Harmonisation

IFNα2, IFNγ	Interferon α2 and γ respectively
IgG1, IgM	Immunoglobin G1 and M respectively
IL-1α, IL-1β, IL-6, IL-12,	Interleukin α, 1β, 6, 12 respectively
INR	International Normalised Ratio
i.v.	Intravenous
LDH	Lactate Dehydrogenase
LPS	Lipopolysaccharide
MTD	Maximally Tolerated Dose
NSCLC	Non Small Cell Lung Cancer
ORR	Objective Response Rate
PD	Progressive Disease
PR	Partial Response
PTT	Partial Thromboplastin Time
RECIST	Response Evaluation Criteria in Solid Tumour
SAE	Serious Adverse Event
SCCHN	Squamous Cell Carcinoma of the Head and Neck
SD	Stable Disease
SOP	Standard Operating Procedures
T_{max}	Time at which maximum concentration is observed
TNFα	Tumour Necrosis Factor α
ULN	Upper Limit of Normal

PROTOCOL SYNOPSIS 4

STUDY SCHEMA 5

STUDY DESIGN FLOW CHART 6

GLOSSARY 7

1 INTRODUCTION 12
- 1.1 Background 12
- 1.2 The EnGeneIC Delivery Vehicle (EDV) 13
- 1.3 Paclitaxel 20
- 1.4 Cetuximab 21
- 1.5 Study Disease 23
- 1.6 Investigational Agent: $^{Erbitux®}$EDVs$_{Pac}$ 23
- 1.7 Rationale for Dose Selection 24

2 STUDY OBJECTIVES 28
- 2.1 Primary Objectives 28
- 2.2 Secondary Objectives 28

3 EXPERIMENTAL PLAN 28
- 3.1 Study Design 28
- 3.2 Number of Centres 29
- 3.3 Number of Patients 29
- 3.4 Estimated Study Duration 29

4 PATIENT ELIGIBILITY 30
- 4.1 Inclusion Criteria 30
- 4.2 Exclusion Criteria 31

5 PATIENT ENROLLMENT 33
- 5.1 Treatment Assignment 33

6 TREATMENT PLAN 33
- 6.1 $^{Erbitux®}$EDVs$_{Pac}$ Administration 33
- 6.2 Allocation to Study Treatment/Dose Escalation Schedule 34
- 6.3 Dose Escalation Rules 35
- 6.4 Definition of Dose-Limiting Toxicity 36
- 6.5 Duration of Therapy 36

6.6 Pre-medication ... 37
6.7 Management of Toxicity ... 37
6.8 Concomitant Medication and Supportive Care Guidelines ... 41

7 STUDY PROCEDURES ... 42
7.1 Screening ... 42
7.2 Treatment Phase ... 43
7.3 Safety Follow Up ... 44
7.4 Specific Procedures ... 45

8 WITHDRAWAL AND REPLACEMENT OF PATIENTS ... 47

9 SAFETY REPORTING ... 48
9.1 Definitions ... 48
9.2 Adverse Event Reporting ... 49
9.3 Serious Adverse Event Reporting ... 50

10 INVESTIGATIONAL AGENT ... 52
10.1 Packaging and Formulation ... 52
10.2 Labelling ... 52
10.3 Storage ... 52
10.4 Preparation and Dispensing ... 52
10.5 Supply and Return of Investigational Agent ... 53
10.6 Accountability ... 54

11 DATA ANALYSIS AND STATISTICAL METHODS ... 54
11.1 Sample Size Determination ... 54
11.2 Methods of Analysis ... 54

12 ADMINISTRATIVE, REGULATORY AND LEGAL OBLIGATIONS ... 57
12.1 Compliance with Good Clinical Practice and Ethical Considerations ... 57
12.2 Informed Consent ... 57
12.3 Human Research Ethics Committee Review ... 58
12.4 Pre-study Documentation Requirements ... 58
12.5 Patient Confidentiality ... 58
12.6 Protocol Amendments ... 59
12.7 Study Termination ... 59
12.8 Monitoring ... 59

12.9 Data Collection 60
12.10 Study Documentation and Archiving 60
12.11 Study Reporting and Publication Policy 61
12.12 Compensation 61

13 REFERENCES 62

14 APPENDICES 64
Appendix A. Schedule of Assessments 64
Appendix B. ECOG Performance Status Scale 69
Appendix C. Cockcroft Gault Formula for Calculated Creatinine Clearance 70
Appendix D. Adverse Event Assessments 71
Appendix E. RECIST Criteria 72
Appendix F. Cytochrome P450 inducers/inhibitors 73

1 INTRODUCTION

1.1 Background

Patients with advanced solid tumours are most commonly treated with chemotherapeutic agents – small cytotoxic molecules. This treatment is often associated with limited efficacy and is widely distributed through the body leading to toxicity in normal tissue (low therapeutic index).

Research over the last decade has sought to overcome these twin defects of toxicity and low therapeutic index. Less toxic agents have been developed but they have limited effectiveness. Drugs that are directed at molecular targets have also been developed, as have antibodies that attach to specific molecules on the surface of cancer cells and potentially interfere with the cancer cell's growth. However, with only a few notable exceptions, these new therapies have generally provided only incremental advances compared to the conventional cytotoxic chemotherapeutic drugs and these agents are often associated with novel toxicities. To date, cytotoxics such as cisplatin, doxorubicin, paclitaxel and irinotecan are still the main weapons in the oncologists' armoury.

The EnGeneIC Delivery Vehicle (EDV) developed at EnGeneIC Pty Ltd, Australia has been shown to address both toxicity and low therapeutic index in pre-clinical animal studies (MacDiarmid *et al.*, 2007a and MacDiarmid *et al.*, 2007b). EnGeneIC's approach packages the toxic drug or compound into a 400nm particle, a minicell, derived from a bacteria which carries an antibody targeting specific cancer cells. The antibodies attach to the surface of cancer cells and the EDV is internalised by the cancer cell. The toxic agents are not widely distributed throughout the body, so reducing the chance of side effects and intolerability, and the toxic drug or compound is delivered inside the cancer cell. As a result, much less drug is needed to kill the cancerous cell, thus improving the therapeutic index.

When cancer cells establish, they secrete substances that promote the formation of new blood vessels - a process called angiogenesis**.** These blood vessels grow quickly and, unlike normal blood vessels, they are leaky with "holes" ranging from 200nm to 1.2µm. Drug delivery particles such as liposomes are currently believed to effect tumour-targeting by a passive process involving extravasation from the leaky vasculature (Hobbs *et al*., 1998; Yuan *et al*., 1994) that supports the tumour microenvironment. Although it has been shown that the abnormal tumour microenvironment is characterized by interstitial hypertension, and that this phenomenon may limit access of anti-cancer antibody therapeutics, this does not appear to be an absolute barrier as is exemplified by immunoliposomes (Nielsen *et al*., 2002) and antibody conjugated to Quantum Dots (Gao *et al*., 2004). This phenomenon also holds true for the EDV which has the added advantage of carrying a specifically directed tumour antibody. Following

intravenous injection the EDV extravasates into the tumour microenvironment and this is followed by active targeting via cancer cell-surface receptor engagement and endocytosis as shown below.

1.2 The EnGeneIC Delivery Vehicle (EDV)

EnGeneIC bacterial minicells (EDVs) are anucleate nanoparticles produced as a result of inactivating the genes that control normal bacterial cell division thereby de-repressing polar sites of cell fission (Ma *et al*., 2004). The de-repression means that the bacteria divide in the centre as well as at the poles; the polar division resulting in minicells which EnGeneIC has shown can function as leak-resistant, micro-reservoir carriers that allow efficient packaging of a range of different chemotherapeutic drugs. Moreover, in contrast to current stealth liposomal drug carriers like DOXIL (liposomal doxorubicin), for example, that can package only 10^4 molecules per particle (Park, 2002), or "armed antibodies" which can carry less than 5 drug molecules, EDVs can readily accommodate payloads ranging from 1 million to 10 million drug molecules. Further, EDVs can be targeted to over-expressed receptors on the surface of cancer cells using bispecific antibodies (BsAb), which allows highly significant tumour growth-inhibition and/or regression, both *in vitro* and *in vivo*.

Figure 1 below shows the formation of the EDV under transmission electron microscopy (TEM) and scanning electron microscopy (SEM). The bacterial cell is dividing at its pole resulting in a new bacterial cell and a minicell, the EDV, which has the rigid outer and inner membranes (OM and IM) of its parent but without the nucleus.

Figure 1

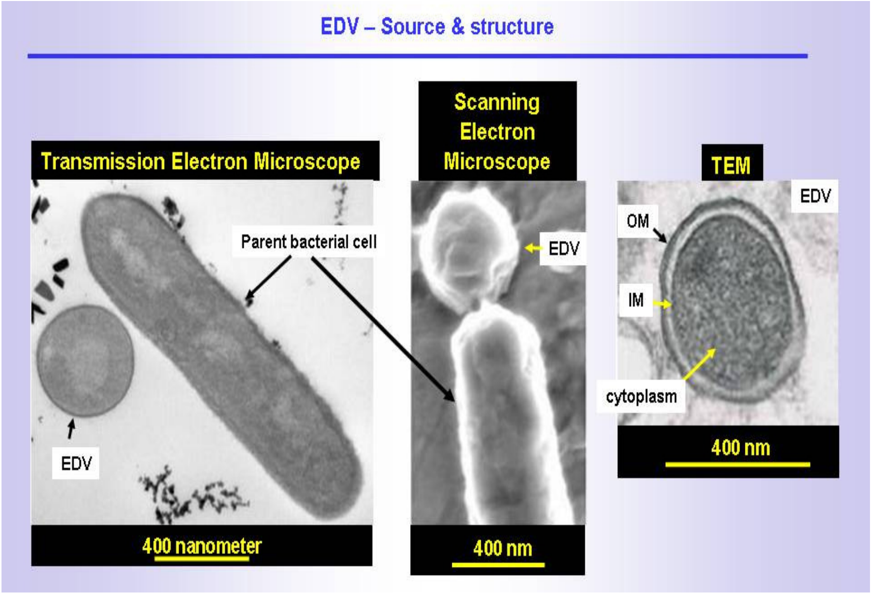

The schematic in Figure 2 shows that drug or nucleic acid-packaged, targeted EDVs specifically attach to the targeted receptor on the cancer cell. Once attached to the cell surface, the EDVs are internalized by the cancer cells via the natural process of receptor-mediated endocytosis and broken down within intracellular compartments called endosomes.

Drugs released from the broken down EDVs leak out of the endosomal membrane and enter the cytoplasm of the cancer cell. The chemotherapeutic drugs then bind to the target molecules and exert their cytotoxic effects. For a detailed discussion of the mechanism of action of EDVs please refer to MacDiarmid *et al*., 2007a.

Figure 2

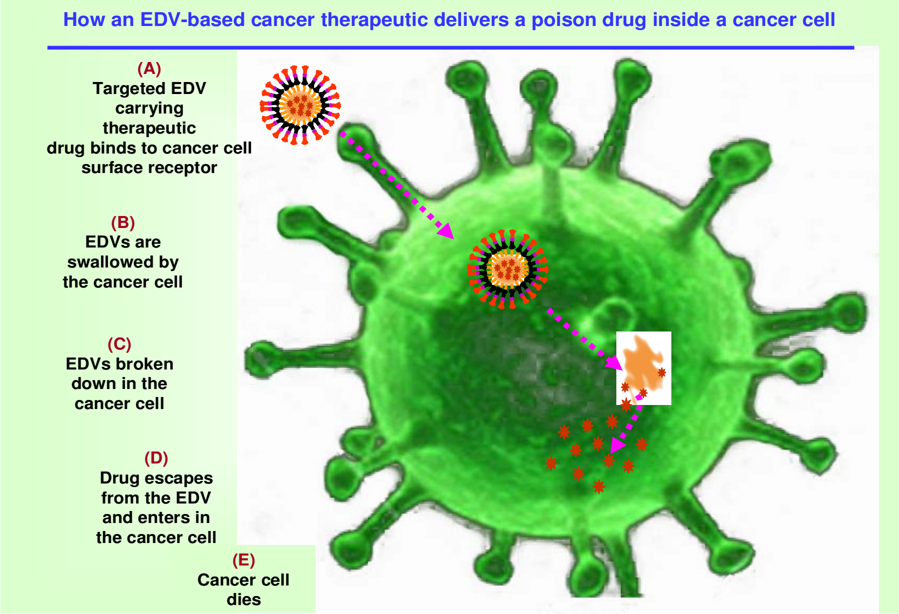

Drug loading of EDVs is likely by diffusion down a concentration gradient with entry via non-specific porin channels (Nikaido, 2003) in the outer membrane. Detailed studies of porins have revealed charged residues within the channels which orient polar solutes during permeation (Schultz, 1993). Hydrophobic solutes may diffuse non-specifically across the membrane via the FadL family of outer membrane proteins (Van den Berg, 2005). Retention of drug in EDVs, after loading, is likely due to the metabolic inactivity that results from their lack of bacterial genome.

<u>Preclinical studies in mice</u>

1. **<u>Xenograft efficacy studies</u>.**

An extensive series of *in-vivo* mouse xenograft studies have been carried out to determine the therapeutic efficacy against a variety of different human tumour xenografts and with different targeting antibodies. Intravenous administration of receptor-targeted, drug-packaged EDVs appear to achieve passive targeting by falling out of the leaky vasculature surrounding the tumour and this is followed by active targeting of tumour cells by receptor-mediated endocytosis. Therefore, unlike non-targeted liposomal vectors, the drug is delivered intracellularly in tumour cells and not in the extracellular tumour microenvironment.

Some examples include:

**Human tumour xenograft**	**Treatment.**<br>**Series of controls included in each expt; 11 mice per group.**	**Results**	**Drug conc. delivered via EDVs compared to free drug**
Breast<br>MDA-MB-468	EGFR-targeted, paclitaxel-packaged EDVs (^{EGFR}EDVs$_{Pac}$)	Highly significant tumour regression/ stabilization (studied over 84 days) via ^{EGFR}EDVs$_{Pac}$.	8,000-fold lower
Breast<br>MDA-MB-48	EGFR-targeted, doxorubicin-packaged EDVs (^{EGFR}EDVs$_{Dox}$)	Highly significant tumour stabilization & regression of large tumours (studied over 79 days) via ^{EGFR}EDVs$_{Dox}$.	1,875-fold lower
Lung<br>A549 NSCLC	EGFR-targeted, paclitaxel-packaged EDVs (^{EGFR}EDVs$_{Pac}$)	Highly significant tumour regression/ stabilization (studied over 56 days) via ^{EGFR}EDVs$_{Pac}$. Also showed that if receptor is not present on tumour cells i.e. CD33 on lung cancer cells, then ^{CD33}EDVs$_{Pac}$ had no effect on tumour growth.	800-fold lower
Ovarian<br>SKOV3	HER2/*neu*-targeted, Dox-packaged EDVs (^{HER2}EDVs$_{Dox}$).	Highly significant tumour stabilization (studied over 31 days) via ^{HER2}EDVs$_{Dox}$.	150-fold lower
Breast<br>MDA-MB-468	^{EGFR}EDVs$_{Dox}$ and compared to DOXIL (liposomal-Dox; ALZA-J & J).	Highly significant tumour stabilization (studied over 41 days) via ^{EGFR}EDVs$_{Dox}$. DOXIL carrying the same amount of Dox as EDVs had no anti-tumour effect. DOXIL carrying 100-fold higher Dox	150-fold lower

		compared to EDVs had similar anti-tumour effect.	
Promyelocytic leukemia<br><br>HL60	^{CD33}EDVs$_{Dox}$ compared to $^{non-targeted}$EDVs$_{Dox}$.	Highly significant tumour stabilization (studied over 33 days) with ^{CD33}EDVs$_{Dox}$ carrying just 1 µg Dox.	NA

Figure 3 shows an MDA-MB-468 breast cancer xenograft study in nude mice where the animals were given either non-targeted EDVs$_{Pac}$ (0.05 µg Pac/dose) or free Pac (20 µg/gm; ~ 400 µg/mouse) as controls. Inhibition of tumour growth is evident by day 25 in the groups receiving ^{EGFR}EDVs$_{Pac}$ ($p < 0.0003$ and < 0.0006 for the G7 and G8 groups *vs* all others, respectively), but not in the other groups. Groups were dosed either intravenously (i.v.) or directly into the tumour (i.t.).

Figure 3

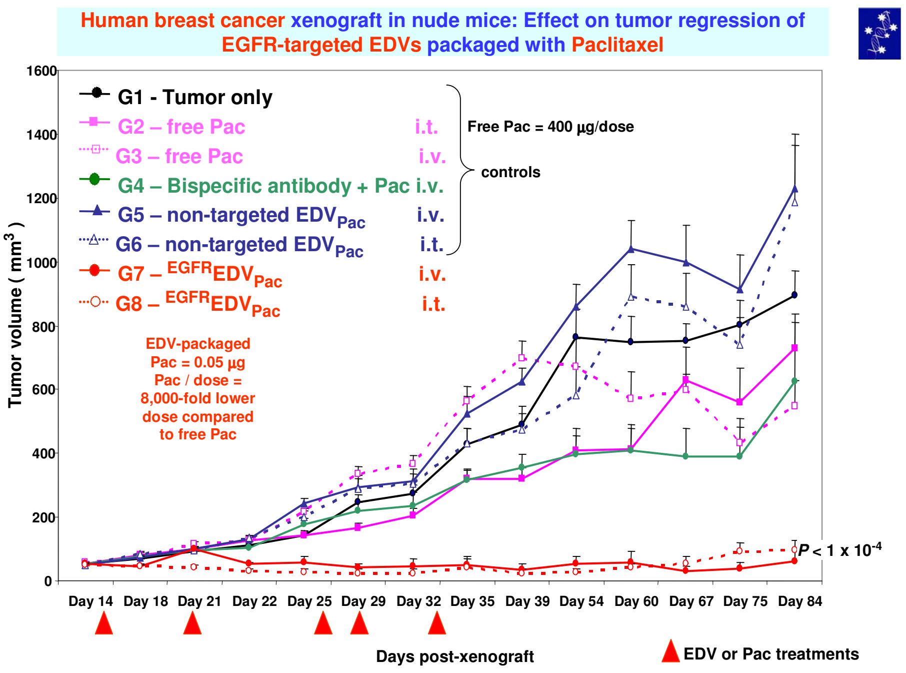

To demonstrate that BsAb-mediated targeting of tumour cells is necessary for the anti-tumour effects, A549 lung carcinoma xenograft study was carried out with a range of different controls (G1 to G10 mice) as shown in Figure 4. The experimental groups consisted of ^{EGFR}EDVs$_{Pac}$ (G11; A549 over-expresses EGFR) and ^{CD33}EDVs$_{Pac}$ (G12; A549 does not express CD33). Inhibition of tumour growth was evident in G11 mice and not in any other group including G12. On day 46, when the tumour volume was ~ 400 to 500 mm^3, the treatment of G12 mice was changed to ^{EGFR}EDVs$_{Pac}$ which resulted in rapid tumour regression ($p < 0.0003$ at

day 56 between G12 *vs* all groups). All treatments were administered via the i.v. route.

Figure 4

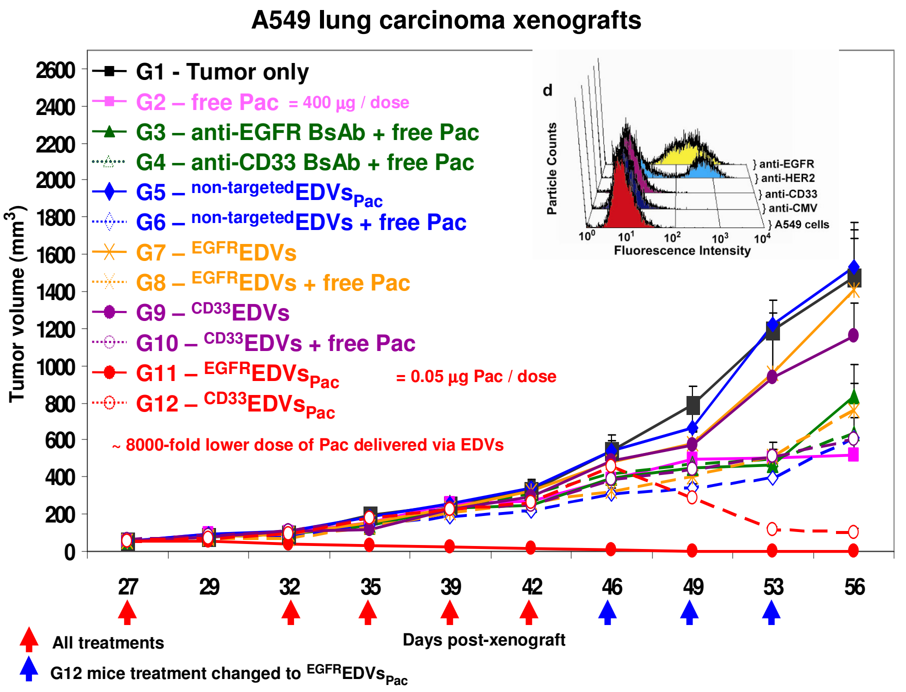

2. Biodistribution

The biodistribution of i.v. administered EDVs bearing ^{125}I-labeled BsAbs was studied in nude mice with MDA-MB-468 xenografts that over express EGFR. These studies revealed that at 2 hrs post-treatment ~ 30% of specifically targeted ^{EGFR}EDVs were localized in the tumour, as compared to only ~ 1.3% of non-specifically targeted ^{CMV}EDVs. In addition, in animals given radio-iodinated BsAb alone (^{EGFR}BsAb or ^{CMV}BsAb), only ~3 and 2.2%, respectively, of the radiolabel was localized in the tumour at 2 hrs. By 6 and 24 hrs, ~ 4.6% and ~ 0.5%, respectively, of specifically targeted ^{EGFR}EDVs remained in the tumours. These data suggest that in contrast to non-specifically targeted ^{CMV}EDVs, ^{EGFR}EDVs are not only localized but are concentrated in the tumour microenvironment. This is likely to result from rapid extravasation of ^{EGFR}EDVs from the circulation via the leaky vasculature of the tumour (passive targeting), followed by engagement of the tumour cell-surface EGFRs (active targeting), by their adherence and internalization. While non-specifically targeted ^{CMV}EDVs may also rapidly extravasate from the circulation, they are not retained in the tumours as they are unable to target EGFRs, and are washed out after tumour isolation and then extensive saline washing of the tissue.

The biodistribution of i.v. administered non-targeted EDVs$_{Dox}$ (non-targeted Dox-packaged EDVs), ^{EGFR}EDVs$_{Dox}$ (EGFR-targeted Dox-packaged EDVs) and free Dox was evaluated in nude mice with breast cancer xenografts (Figure 5).
When the tumour volume reached 140mm^3 to 170mm^3, mice were randomly divided into three groups of nine mice per group. G1, G2 and G3 mice received i.v. free Dox (150 µg/dose), non-targeted EDVs$_{Dox}$ or ^{EGFR}EDVs$_{Dox}$ (~74 µg Dox/dose contained in 5 x 10^9 EDVs), respectively. Post-treatment, three mice from each group were euthanized at 6 hrs and 24 hrs and plasma and tissue harvested and frozen for extraction and determination of Dox concentration using ESI and Maldi or LC-MS/MS.

At 6 hrs, ~ 30% of the Dox dose administered by the ^{EGFR}EDVs$_{Dox}$ was found in the tumours, as compared to only ~ 1% of the free Dox and ~ 0.34% of that in the non-targeted EDVs$_{Dox}$. Thus targeted EDV delivery provides at least a 30-fold enrichment in tumour drug-delivery.

Figure 5

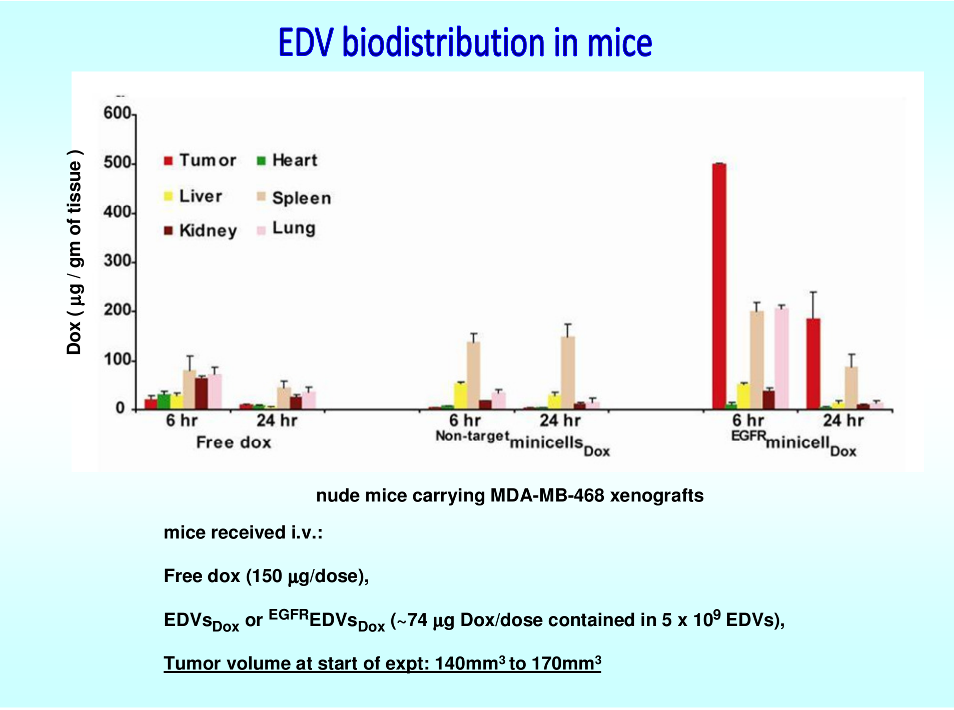

<u>Pre-clinical studies in Dogs</u>

Canine hemangiosarcoma is equivalent to angiosarcoma in humans and commonly involves the spleen, liver and heart. The disease has a grave prognosis and the median survival time is six months even with surgery and chemotherapy (Fosmire *et al*., 2004). Most dogs with heart lesions are unable to have surgery which limits their survival time further.

A total of 14 dogs suffering from hemangiosarcoma have been treated with EDVs loaded with doxorubicin and targeted to the c-kit receptor (CD117), ^{CD117}EDV$_{Dox}$. Dogs have been dosed weekly with between 2 x 10^9 ^{CD117}EDV$_{Dox}$ and 1x10^{10} ^{CD117}EDV$_{Dox}$.

All dogs have shown anti-tumour effects and 11 out of 14 dogs are still undergoing treatment. Three dogs have been euthanased at the behest of their owners since their tumours returned after initial response and were presumably resistant to doxorubicin.

The number of days survival post EDV treatment and the number of doses given to each dog so far is shown in Figure 6. The EDVs have been well-tolerated up to the highest dose given, 1 x 10^{10}.

Figure 6

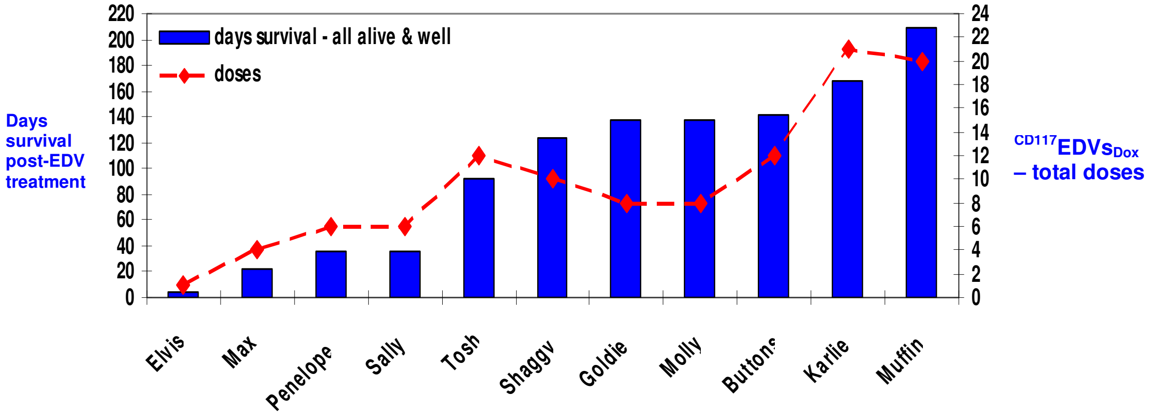

Pre-clinical safety studies in Animals

Monkeys

Studies carried out in rhesus monkeys are outlined in section 1.7. Briefly, a total of 96 monkeys have been dosed with unloaded, untargeted EDVs and ^{EGFR}EDVs$_{Dox}$ (where the anti-EGFR was targeted to monkey EGFR) and $^{Erbitux®}$EDVs$_{Pac}$ (where the anti-EGFR was targeted to human EGFR). Inflammatory cytokines, TNFα and IL6 were transiently elevated following each dose of 2 x 10^9 ^{EGFR}EDVs$_{Dox}$ and 1 x 10^{10} $^{Erbitux®}$EDVs$_{Pac}$ and were in the mild inflammation range. Monkeys dosed with 1 x 10^{11} $^{Erbitux®}$EDVs$_{Pac}$ showed moderate inflammation. No clinical EDV-related side effects including increase in temperature were seen for any dose in the range from 10^8 to 10^{11} EDVs. Haematological and biochemical parameters were normal throughout the study.

Dogs

None of the dogs in the above study exhibited clinical side effects associated with the EDV treatment apart from a temperature spike at 4 hours if the endotoxin was measured at greater than 20EU per EDV dose. If this temperature spike occurred, it was also associated with a rise in inflammatory cytokines and occasional episode of emesis. Cancer-related changes in haematological and biochemical parameters were observed for some animals but these changes were considered to be unrelated to targeted EDVs.

1.3 Paclitaxel

Paclitaxel is a taxane which is derived from the bark of the Pacific yew tree. Its mechanism of action is to bind to tubulin, thereby stabilising microtubules. The resulting microtubule/paclitaxel complex does not have the ability to disassemble. This adversely affects cell function because the shortening and lengthening of microtubules (termed dynamic instability) is necessary for their function as a mechanism to transport other cellular components such as chromosomes during their replication (Kumar *et al*., 1981).

Common side-effects include nausea and vomiting, loss of appetite, change in taste, thinned or brittle hair, pain in the joints of the arms or legs lasting 2-3 days, changes in the color of the nails, and peripheral neuropathy. The major haematological toxicity is profound but short-lived neutropenia. The clinical toxicity of paclitaxel, including hypersensitivity is also associated with the solvent Cremophor in which it is dissolved for delivery since it is very hydrophobic. Dexamethasone is given prior to beginning paclitaxel treatment to mitigate some of the side effects.

In recent years, extensive research has been done to find a way to mitigate the side effects of paclitaxel, by altering its administration. Cell Therapeutics has formulated PG-paclitaxel, which is paclitaxel bonded to a polyglutamate polymer. PG-paclitaxel has been introduced into clinical use, and has been shown to reduce side effects and to effectively treat many patients who were not responsive to the action of Taxol. The PG-paclitaxel may be a very promising anti-cancer drug, as it is much more selective than paclitaxel for which cells it targets (Whelan, 2002). The newest form of paclitaxel, Abraxane, in which paclitaxel is bonded to albumin, has also been developed by Abraxis Bioscience as an alternative to the often toxic solvent delivery method (Desai, 2006). In phase 3 trials for metastatic breast cancer, Abraxane showed less grade 3 neutropenia than conventional paclitaxel and gave an improved therapeutic index (Gradishar *et al*., 2005). Abraxane was approved by the Food and Drug Administration in January 2005 for the treatment of breast cancer after failure of combination

chemotherapy for metastatic disease or relapse within six months of adjuvant chemotherapy (FDA, 2005).

Paclitaxel is approved for lung cancer, ovarian cancer and breast cancer but has shown to be active in a wide range of cancers including head and neck, oesophageal and gastric cancers (Foa *et al*., 1994). Since paclitaxel has the potential to be effective in a wide range of cancers, and it has significant toxicity, it is therefore an ideal candidate for evaluation with a novel delivery system which has the potential to dramatically increase therapeutic index. The amount of drug delivered by a dose of 10^9 targeted EDVs is *less than 1µg* compared to *135mg to 175mg per* m^2 *equating to 225,000 µg to 292,000 µg* for paclitaxel infusion in an average 60kg patient.

1.4 Cetuximab

Over-expression of the epidermal growth factor receptor (EGFR) in a large percentage of solid tumour types is associated with aggressive disease and poor clinical prognosis. In normal and malignant cells, the activation of EGFR receptor cascades has multiple consequences, such as cell growth, differentiation, and proliferation (Holbro *et al*., 2003). The EGFR signalling pathway may also promote malignant transformation, angiogenesis, and metastatic dissemination (Holbro *et al*., 2003). To block the activation of this receptor, targeted therapies including monoclonal antibodies have been developed, representing a new and promising strategy for cancer management (Lockhart and Berlin, 2005). One such antibody directed against EGFR which has recently been integrated into the treatment of metastatic colorectal carcinomas (CRC) resistant to chemotherapy is cetuximab marketed as Erbitux (Cunningham *et al*., 2004). Cetuximab was also recently approved by the FDA in March 2006 for use in combination with radiation therapy for treating squamous cell carcinoma of the head and neck (SCCHN) or as a single agent in patients who have had prior platinum-based therapy (Zimmerman *et al*., 2006). Despite the over expression of EGFR in many tumour types, however, cetuximab has not proven to give dramatic tumour responses e.g. approximately 12% response rate and 1.4 months progression-free survival in CRC (Lenz *et al*., 2006).

Cetuximab is believed to operate by binding to the extracellular domain of the EGFR of all cells that express EGFR, preventing ligand binding and activation of the receptor. This blocks the downstream signalling of EGFR resulting in impaired cell growth and proliferation. Cetuximab has also been shown to mediate antibody dependent cellular cytotoxicity. Since EGFR is found on many normal cells including skin and lung, Cetuximab is also responsible for side effects such as an acne-like rash, breathlessness and flu-like symptoms. The table below indicates the occurrence of EGFR in different tumour types (Merck KGA, Background communication, 2006).

Tumour type	Percentage of tumours over-expressing EGFR
Head and neck	90 - 100
Colorectal	75 - 89
Prostate	Up to 100
Pancreatic	Up to 95
Breast	Up to 91
Renal	Up to 90
Cervix	Up to 90
NSCLC	Up to 80
Ovarian	Up to 77
Bladder	Up to 72

Recently, much research has been centred on "armed antibodies" where toxins, radionucleotides or chemotherapeutics are attached to the antibody. The antibody is internalised and cell killing occurs because of the drug payload. The number of drug molecules attached is limited to 2-5 per antibody and efficacy is likely to be severely compromised and it is probable that similar side effects would be seen as with antibody alone.

The EDV delivery system does not rely on any therapeutic effect of the antibody which is used to target the drug payload – the antibody merely locks onto the cancer cell, is internalised and allows the cell to "commit suicide" when the payload of paclitaxel is delivered intracellularly. Erbitux® appears to be a very good candidate for the EDV since it has already been approved by the US FDA and the Australian TGA and the amount of targeting bispecific antibody (comprised of Erbitux®--protein A/G--anti-O-polysaccharide) used in this strategy is less than *5 µg* to saturate 2 x 10^{11} EDVs compared with widely used conventional doses of Erbitux® at *250- 400 mg per* m^2 *equating to 668,000 µg for an average 60 kg patient dose*.

Summary

Chemotherapeutic drug therapy for cancer is hindered by severe toxicity due to indiscriminate drug distribution and collateral damage to normal cells. In order to address the therapeutic index issue, EDVs loaded with chemotherapy and targeted to cancer cells have been developed. They have been shown to be safe in toxicology studies in normal monkey and efficacious in mouse xenografts and dogs suffering from cancer. It has also been shown that in mouse biodistribution studies, 30% of EDVs extravasate into the tumour microenvironment and deliver their payload intracellularly within 6 hours.

CONFIDENTIAL

Due to the toxicities associated with free paclitaxel and considering the dramatic reduction in the amount of drug required when packaged via the EDV, ^{EGFR}EDV$_{Pac}$ is worth exploring in a Phase 1 trial for patients with advanced solid tumours.

1.5 Study Disease

Tumours expressing the EGFR will be studied. These include, but are not limited to, tumour types such as head and neck squamous cell carcinoma, oesophageal, non small cell lung, colorectal, pancreatic, gastric, cervical/uterine, endometrial, bladder and prostate cancers.

1.6 Investigational Agent: $^{Erbitux®}$EDVs$_{Pac}$

The $^{Erbitux®}$EDVs$_{Pac}$, which has been assigned the code EDV1-1, is a three-component delivery system (as shown in Figure 7 below) consisting of a complex formed by:

1. EnGeneIC Delivery Vehicle (EDV): A non-living, spherical 400 nm particle (EDV; also known as minicell) derived from a genetically modified strain of *Salmonella Typhimurium*.
2. Paclitaxel which is loaded inside the EDV by incubating EDVs in a solution of 100 µg / ml of paclitaxel for 1 hr followed by extensive washing of the paclitaxel-packaged EDVs (EDVs$_{Pac}$).
3. A bispecific antibody complex attached to the surface of the EDVs$_{Pac}$ to target it specifically to tumour cells expressing EGFR. The complex is composed of:
   a. Cetuximab (Erbitux®, Bristol-Myers Squibb), a monoclonal antibody directed against the epidermal growth factor receptor (EGFR).
   b. Recombinantly produced Protein A/G which is a genetically-engineered protein that combines the IgG binding domains of both Protein A and Protein G. It is a gene fusion product expressed in *E. coli*. In this therapeutic, Protein A/G binds both the targeting EGFR antibody and the murine monoclonal described below, to form a bispecific complex.
   c. A purified murine monoclonal antibody directed against lipo-polysachharide (LPS) on the surface of the EDV, which anchors the targeting complex to the EDV.

Figure 7

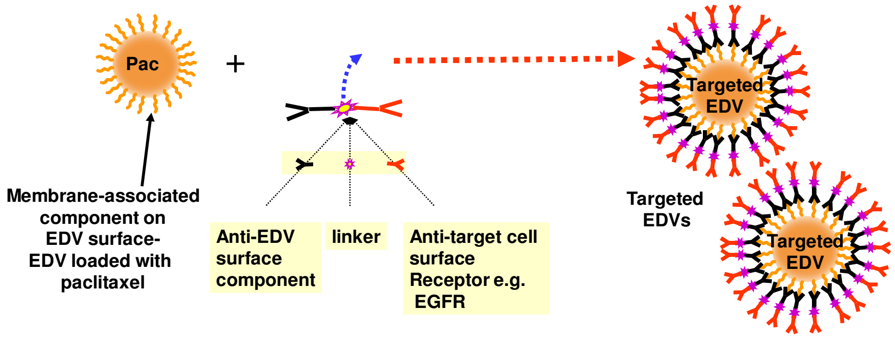

Erbitux® $EDVs_{Pac}$ will be supplied as a lyophilized powder which will be reconstituted in normal saline.

1.7 Rationale for Dose Selection

The dose escalation regime has been chosen based on the results of the following pre-clinical toxicology studies.

<u>**Non-Human Primate Studies**</u>
Three separate safety studies have been carried out in normal rhesus monkeys at the National Chengdu Centre for Safety Evaluation of Traditional Chinese Medicine:

1. <u>**Study 1**</u> - The toxicity of non-targeted and non-drug loaded EDVs was evaluated in male and female Rhesus monkeys (3/sex/dose) given five doses of 0 (saline control), 1×10^7, 1×10^8 or 1×10^9 EDV / per monkey, one dose every seven (7) days. Throughout the experimental period there was no mortality or morbidity observed. No significant changes in food or water consumption were observed during the treatment period. There were no obvious changes in cage observations, detailed clinical examinations or ophthalmoscopic examinations during the course of the trial. There were no remarkable EDV induced changes in clinical pathology parameters or circulating cytokine levels and empty EDVs exhibited low immunogenicity. There were also no changes in urine analysis parameters or any abnormal gross pathological phenomena. Although some spontaneous histopathological changes were observed, none of these changes were EDV-

related.

This trial showed that EDVs were safe in monkeys at doses up to 10^9 particles.

2. **Study 2** - The objective of this study was to evaluate the potential toxicity of doxorubicin-loaded EDVs which carry a bispecific antibody directed towards the *monkey* epidermal growth factor receptor in monkeys when administered by intravenous injection, every seven (7) days for 5 weeks. The following dosing scheme was applied to 36 monkeys (19 males, 17 females) that were divided into the following groups.
   a. Saline control
   b. 2 x 10^{10} EDVs
   c. 2 x 10^9 EDVs$_{Dox}$ (non-targeted)
   d. 2 x 10^{10} EDVs$_{Dox}$ (non-targeted)
   e. 2 x 10^9 ^{EGFR}EDVs$_{Dox}$
   f. 2 x 10^{10} ^{EGFR}EDVs$_{Dox}$

Throughout the experimental period of this trial, there was no mortality or morbidity observed. The conclusion from the Chengdu centre was "EDVs did not cause any toxicologically relevant effects in rhesus monkeys, some parameters showed statistically significant changes in treatment groups compared with control group, but most of them fluctuated within the normal range according to the background established in this centre. A slight vascular inflammation in livers occurred in both treatment and control groups of monkeys and could not be determined a toxic effect of test articles. Collectively, it could be concluded that no evident toxic effects related to EDVs were found in rhesus monkeys in this study".

No significant elevation in body temperature, which is an indication of an inflammatory response, was observed at any dose level nor following any of the five doses. The cytokines, TNFα and IL6 were measured at 0, 4 and 24 hours post dosing for each dose. TNFα was mildly elevated (<100 pg/ml) in the group receiving 2 x 10^{10} ^{EGFR}EDVs$_{Dox}$ but not in the groups receiving 2 x 10^{10} EDVs$_{Dox}$ (no targeting) or EDVs alone. This would suggest that the crude preparation of the anti-monkey EGFR bispecific antibody is capable of eliciting a mild TNFα response in normal monkeys at this dose.

At 4 hrs IL-6 was also mildly elevated at a dose of 2 x 10^{10} EDVs and 2 x 10^{10} EDVs$_{Dox}$ but not for 2 x 10^{10} ^{EGFR}EDVs$_{Dox}$, except in the first dose. IL-6 levels returned to baseline by 24 hours.

The C-Reactive Protein (CRP) levels in this study increased by 5 – 25 fold in the 2 x 10^9 EDV groups and from 25 – 100 fold in the 2 x 10^{10} EDV groups at 24 hours post-injection, indicative of mild inflammation. The duration of the response was very short and 72 hours post-injection CRP levels had returned to baseline.

CONFIDENTIAL

Considering that these monkeys are non-tumour bearing, the majority of the EDVs are expected to localize in the liver where they would be processed by macrophages in the hepatic sinusoids of the liver (Kupffer cells). As EDVs are processed by macrophages, cytokines such as TNFα and IL-6 are released which stimulate hepatocytes to release CRP. The fact that these monkeys did not exhibit any symptoms of fever or an increase in liver enzymes, combined with a transient low level IL-6 spike at 4 hours, suggests that the intermediate CRP spike produced by these monkeys is simply related to the rapid but short-lived processing of EDVs in the liver giving EDVs a good safety profile.

Antibodies to O-polysaccharide (major Immunogen) of *S. typhimurium* were measured 6 days after each dose. There was a very low titre increase in IgG1 and IgM antibodies after the second dose but no amnestic response.

This trial showed that at 2 x 10^{10} EDVs there was a mild inflammatory response as shown by IL-6 and TNFα**.**

3. **<u>Study 3</u>** – The objective of this trial was to carry out an escalating dose of EDVs in rhesus monkeys using the same construct as to be used in this Phase 1 study, i.e. $^{Erbitux®}$EDVs$_{Pac}$ where the anti-EGFR was cetuximab (Erbitux®, Bristol Myer Squibb).

   The doses were 1 x 10^8, 1 x 10^9, 1 x 10^{10}, 1 x 10^{11} and saline control with 6 monkeys in each group.

   Throughout the experimental period there was no mortality or morbidity observed.

   All haematology and biochemistry analyses were unremarkable.
   At 24hrs post dosing, C-reactive protein as in Trial 2 was mildly elevated (mild inflammation) in the group receiving 1 x 10^{10} $^{Erbitux®}$EDVs$_{Pac}$ and elevated (acute inflammation) in the group receiving 1 x 10^{11} $^{Erbitux®}$EDVs$_{Pac}$. The response had returned to baseline when measured prior to subsequent doses.

Studies in Pigs

Eleven-week old female white healthy pigs (3) were dosed with EDVs as per the following regime:
Baseline: day 1 (injection 1, 1 x 10^9)
Treatment 1: day 8 (injection 2, 5 x 10^9)
Treatment 2: day 15 (injection 3, 4 x 10^9)
Treatment 3: day 24 (injection 4, 6 x 10^9)
Treatment 4: day 57 (injection 5, 4 x 10^9)
Treatment 5: day 72 (injection 6, 6 x 10^9)

One pig was dosed with saline as a control. Animals were dosed with EDVs weekly for 3 weeks (short-term phase), then rested for 4 weeks prior to 2 doses administered fortnightly (long-term phase).

The animals showed no changes in blood chemistries, haematological indices, serum biochemistry, feed intake, growth rate and body temperature. There was a mild rise in inflammatory cytokine TNFα which returned to normal by 4hrs post- injection. Other inflammatory cytokines like IL-6 and haptoglobin levels remained normal.

Canine Studies

Case studies have been carried out in dogs suffering from hemangiosarcoma and Non-Hodgkin's lymphoma. Targeted and doxorubicin loaded EDVs administered to dogs weekly and a number of dogs have received up to 20 doses of EDVs (see Figure 6). No EDV-related toxicities in haematology or biochemistry have been observed with doses up to 1×10^{10}. It has been observed that if the free endotoxin levels in the EDV preparation exceed 20EU per dose, some dogs showed a transient spike in temperature of 1^0C at 4 hrs and one bout of emesis. This was associated with a rise in TNFα and IL-6 which was self-limiting and returned to normal by 24 hours. This was readily resolved by introducing a filtration step prior to dosing so that the free endotoxin units could be kept below 20EU per dose.

On the basis of the above studies a starting dose level of 1×10^{8} $^{Erbitux®}EDVs_{Pac}$ was selected. The Minimal Anticipated Biological Effect Level (MABEL) seen in the preclinical studies in the most relevant species studied was 2×10^{9} $^{EGFR}EDVs_{Pac}$ where the anti-EGFR was directed against monkey EGFR. Trial 3 showed that the cetuximab- targeted EDV gave mild inflammation (TNFα and CRP elevation) at a dose of 10^{10} $^{Erbitux®}EDVs_{Pac}$. Taken together, and because of the novelty of the approach a safety margin of 1/20 of the MABEL will be used for the starting dose. This is more conservative than the use of a No Observed Adverse Effect Level (NOAEL).

2 STUDY OBJECTIVES

2.1 Primary Objectives

- To evaluate the safety, tolerability and maximal tolerated dose (MTD) of $^{Erbitux®}EDVs_{Pac}$ in patients with advanced solid tumours.

2.2 Secondary Objectives

- To determine immune and inflammatory responses to intravenously administered $^{Erbitux®}EDVs_{Pac}$
- To document any evidence of anti-tumour activity using CT and FDG-PET.

3 EXPERIMENTAL PLAN

3.1 Study Design

This is a phase 1, multicentre, open label, dose escalation, safety study examining the safety of repeated doses of $^{Erbitux®}EDVs_{Pac}$.

There are 2 parts to this study. In part 1, patients will be administered a cycle of $^{Erbitux®}EDVs_{Pac}$. Each cycle consists of 5 infusions of $^{Erbitux®}EDVs_{Pac}$ administered at weekly intervals followed by a week free of treatment. After administration of the study drug, patients will be followed up in hospital for a period of 6 hours after which they will be permitted to go home providing there are no safety issues. Patients will return 24 hours after the dose for blood tests and an ECG. From cycle 2 onwards, patients will be observed in the clinic for a period of 4 hours after administration of study drug.

Each new cycle will commence immediately after the last cycle (i.e., 14 days after the last dose was administered in the previous cycle) unless prohibited by toxicities (see section 6.7 for details).

After each cycle, patients will undergo a reassessment of tumour status with CT scans. This should occur on days 36-42 of the cycle. If there is stable disease or if there is a response according to RECIST criteria, a further cycle of 5 doses of $^{Erbitux®}EDVs_{Pac}$ at weekly intervals will be administered to the patient. If there is progressive disease and the patient has no other proven treatment options available,the patient may receive a further cycle of treatment provided there are no unacceptable safety concerns and they continue to have adequate organ function as defined in section 4.1.6.
Once a decision has been made to discontinue treatment, patients will return for a follow up safety visit 30 days after the administration of their last dose. If a patient discontinues treatment within the first cycle he/she should return for an

additional follow up visit at 14 days after administration of the last dose of $^{Erbitux®}$EDVs$_{Pac}$.

Each patient will remain on the dose level at which they commenced the study, except in certain situations following dose limiting toxicities where the dose may be reduced for subsequent cycles (see section 6.4).

The dose of $^{Erbitux®}$EDVs$_{Pac}$ will escalate according to the dose escalation schedule and dose escalation rules outlined in Sections 6.2 and 6.3.

In part 2, all patients will be administered the recommended phase 2 dose as identified in part 1 of the study. The treatment and assessment schedule used in part 1 will apply also in part 2. However, at baseline and after cycle 1, tumour status will be assessed using both CT and FDG PET scans.

3.2 Number of Centres

Three centres in Melbourne will participate in the study.

3.3 Number of Patients

The number of patients that will be treated in Part 1 of this study is dependent on the tolerability of $^{Erbitux®}$EDVs$_{Pac}$ which will determine how many patients will need to be treated at each level according to the dose escalating rules and which dose level proves to be the maximum administered dose. There are 6 pre-specified dose levels being investigated in this study with 3 patients assigned per dose level (up to a maximum of 6 per dose level).

After completion of dose escalation (part 1) an additional 20 patients will be enrolled and treated at the recommended phase 2 dose in order to further characterise the safety, toxicity and provide a preliminary indication of efficacy of treatment with $^{Erbitux®}$EDVs$_{Pac.}$ These patients will be required to have immunohistochemical confirmation of EGFR expression in their tumours and to have measurable disease.

3.4 Estimated Study Duration

3.4.1 Study Duration for Patients

The duration of study participation for each patient will depend on the patient's response to treatment. A patient who completes only 1 cycle of treatment will be on the study for a period of up to 8 weeks from the start of treatment to the safety follow up visit. However, the treatment period is only 5 weeks.

CONFIDENTIAL

Each cycle will add an additional 6 weeks to the patients' participation. Patients may continue to receive cycles of study treatment until such time as they cease to meet the criteria outlined in Section 6.5.

3.4.2 End of Study

The end of study is dependent on tolerability of the study medication and the length of time patients remain in the study.

4 PATIENT ELIGIBILITY

4.1 Inclusion Criteria

4.1.1 Patients must have histologically or cytologically confirmed advanced solid tumours that are metastatic or unresectable and for which standard curative or palliative measures are not available or are no longer effective.

4.1.2 Patients must have tumour types known, from the clinical and scientific literature, to express the EGFR. The list of EGFR expressing tumours includes, but is not limited to, tumour types such as head and neck squamous cell carcinoma, non small cell lung, colorectal, cutaneous squamous cell, oesophageal, pancreatic, gastric, cervical, endometrial, bladder, and prostate cancers. Patients with other tumour types may be eligible if EGFR expression is confirmed by immunohistochemistry.

4.1.3 Men or women aged ≥ 18 years

4.1.4 ECOG performance status of 0 or 1

4.1.5 Life expectancy of greater than 3 months

4.1.6 Patients must have adequate organ and marrow function as defined below:

- Haemoglobin ≥ 90 g/L
- Leukocytes ≥ 3.0 x 10^9/L
- Absolute Neutrophil Count ≥ 1.5 x 10^9/L
- Platelets ≥100 x 10^9/L
- Total Bilirubin ≤1.5 X upper limit of normal
- AST (SGOT)/ALT (SGPT) ≤2.5 X institutional upper limit of normal (<5X if liver metastases present)
- Creatinine Clearance* ≥60 mL/min for patients with creatinine levels above institutional normal.
- Magnesium Within normal range

** Calculated from serum creatinine using Cockcroft-Gault formula (see Appendix C)*

4.1.7 The effects of Erbitux® $EDVs_{Pac}$ on the developing human foetus are unknown. For this reason women of child-bearing potential and men must agree to use adequate contraception (hormonal or barrier method of birth control; abstinence) for the duration of study participation. Should a woman become pregnant or suspect she is pregnant while participating in this study, she should inform her treating physician immediately.

4.1.8 Ability to understand and the willingness to sign a written informed consent document.

Additional Criteria for Part 2

4.1.9 Patients must have EGFR expression documented by immunohistochemistry from archived or recent primary or metastatic tumour biopsies.

4.1.9 Patients must have measurable disease per RECIST criteria.

4.2 Exclusion Criteria

4.2.1 Previous systemic dose taxanes (paclitaxel or docetaxel); previous low dose paclitaxel (45mg/m^2 weekly) during radiation is permissible

4.2.2 Therapy with an EGFR inhibitor e.g. cetuximab or erlotinib within 30 days prior to first dose.

4.2.3 Patients who have had chemotherapy or radiotherapy, with the exception of palliative radiotherapy, within 4 weeks (6 weeks for nitrosoureas or mitomycin C) prior to entering the study or those who have not recovered from adverse events due to agents administered more than 4 weeks earlier.

4.2.4 Patients may not be receiving any other investigational agents while on study or within 30 days prior to the commencement of study treatment.

4.2.5 Patients who have had vaccination against Salmonella serovars within the last 12 months. Patients who are positive for antibodies to salmonella species

4.2.6 Patients with uncontrolled brain metastases

Patients with a history of brain metastases are eligible if definitive therapy has been administered (surgery and/or radiation), there is no further planned

treatment for the metastases, the patient is stable and off corticosteroids for at least 2 weeks prior to treatment in the study.

4.2.7 Known hypersensitivity to cetuximab infusion or history of allergic reactions attributed to other monoclonal antibodies or taxanes.

4.2.8 Patients with significant pericardial effusions, pleural effusions or ascites.

4.2.9 Unstable diabetes mellitus or other contraindications for the use of dexamethasone.

4.2.10 Uncontrolled concurrent cardiac disease including, but not limited to symptomatic congestive heart failure, unstable angina pectoris, cardiac arrhythmia

4.2.11 Pregnant or breastfeeding women are excluded from this study because the effect of Erbitux® EDVs$_{Pac}$ on the unborn foetus or the nursing infant is unknown.

4.2.12 Known to be human immunodeficiency virus (HIV), hepatitis B surface antigen or hepatitis C positive; or with a history of chronic active hepatitis or cirrhosis.

4.2.13 History of arterial or venous thrombosis within 6 months prior to entry

4.2.14 Peripheral neuropathy > grade 1 per Common Terminology Criteria for Adverse Events (CTCAE v3.0)

4.2.15 Active or uncontrolled severe infection

4.2.16 Major surgical procedures within 28 days prior to entry or failure to recover from prior surgery

4.2.17 Not recovered from all previous therapies (i.e. radiation, surgery and medications). Adverse events related to previous therapies must be CTCAE grade ≤ 1 at entry or returned to the patient's baseline prior to their most recent previous therapy

4.2.18 History of any medical or psychiatric condition or laboratory abnormality that in the opinion of the investigator may increase the risks associated with the study participation or investigational product administration or may interfere with the interpretation of the results

4.2.19 Previous enrolment in this study

4.2.20 Not available for follow-up assessments or unable to comply with study requirements

5 PATIENT ENROLLMENT

Before patients are enrolled into the study EnGeneIC requires a copy of the HREC approval letter clearly identifying the version of the protocol and patient informed consent forms that were approved for each site. All patients must personally sign and date the consent form before any study specific screening procedures are performed. Standard medical practice procedures performed prior to the signing of the consent form, which are performed irrespective of the patients' potential involvement in the study, may be used for screening purposes if they comply with the study requirements.

A target of 3 patients (maximum of 6) will be enrolled at each dose level tested. The second patient at each dose level cannot be treated until a minimum of 2 days have elapsed after the first patient has received their first dose. Similarly, there will be at least a further 2 day delay before third patient can receive their first dose.

Patients who do not meet the eligibility criteria may be rescreened at the discretion of the investigator if their status changes. However, once enrolled, the patient may not be enrolled more than once in the study.

All patients who sign the informed consent form will be deemed as having entered the screening period for the study. At this point each patient will be assigned a unique screening identification number by which they will be identified throughout the screening period. If a patient undergoes a repeat screen they will continue to be identified by the original screening number assigned to them, plus an additional letter or number to denote rescreening.

Day 1 of the first cycle will be considered the beginning of the treatment period and patients who are eligible to receive treatment will be assigned a second unique patient identification number and will be identified by that number throughout the rest of the study.

5.1 Treatment Assignment

Patients will be entered sequentially into a dose level after confirmation of eligibility criteria and dependant on dose level patient numbers (see Section 6.2). All eligible patients will receive open label treatment with $^{\text{Erbitux®}}$EDVs$_{\text{Pac}}$.

6 TREATMENT PLAN

6.1 $^{\text{Erbitux®}}$EDVs$_{\text{Pac}}$ Administration

All patients will be premedicated with a corticosteroid and an antihistamine, as outlined in Section 6.6, prior to receiving study medication.

Each dose of $^{Erbitux®}EDVs_{Pac}$ will be formulated in 20 ml sterile normal saline and is to be administered by infusion over a period of 20 minutes.

For each dose of $^{Erbitux®}EDVs_{Pac}$, it is essential that a medical practitioner be available during the administration of the dose and throughout the observation period. Patients should be observed in an area with access to resuscitation equipment. Close monitoring of vital signs of the patient will take place during this period as outlined in the Schedule of Assessments in Appendix A.

Exact documentation of actual dose, date and time of infusion is required.

6.2 Allocation to Study Treatment/Dose Escalation Schedule

Once eligibility has been confirmed, patients will be assigned to receive $^{Erbitux®}EDVs_{Pac}$ at the dose level which is being investigated at the time of their first dose. They will remain on that dose level throughout their participation in the study. Dose levels may not be increased for a particular patient and may only be decreased in certain situations following dose limiting toxicities (see Section 6.4)

The dose escalation schedule is as follows in Table 1:

Table 1: Dose Escalation Schedule

Dose Level	Dose of $^{Erbitux®}EDVs_{Pac}$
Level 1	1×10^{8} $^{Erbitux®}EDVs_{Pac}$
Level 2	1×10^{9} $^{Erbitux®}EDVs_{Pac}$
Level 3	1×10^{10} $^{Erbitux®}EDVs_{Pac}$
Level 4	5×10^{10} $^{Erbitux®}EDVs_{Pac}$
Level 5	1×10^{11} $^{Erbitux®}EDVs_{Pac}$
Level 6	2×10^{11} $^{Erbitux®}EDVs_{Pac}$

The first patient recruited into the study will be treated with a dose of 1×10^{8} $^{Erbitux®}EDVs_{Pac}$. When 3 patients have completed one cycle of treatment at this dose level, and providing the safety data supports this, the dose will be escalated to 1×10^{9} $^{Erbitux®}EDVs_{Pac}$. Likewise, when 3 patients have safely completed one cycle of treatment at this dose level, the dose will again be escalated to 1×10^{10} $^{Erbitux®}EDVs_{Pac}$ and so forth through the dose levels listed in Table 1 above. Should patients experience dose limiting toxicities, additional patients may be recruited to that dose level and/or dose escalation may cease according to the rules described in Section 6.3.

The animal data with EDVs supports the commencement of dosing at 1×10^{8} $^{Erbitux®}EDVs_{Pac}$. However, in the unlikely event that dose limiting toxicity is seen at this dose level, the dose to be evaluated will be reduced to 5×10^{7} $^{Erbitux®}EDVs_{Pac}$ and if necessary reduced again to $1 X 10^{7}$ $^{Erbitux®}EDVs_{Pac}$.

A central tracking system for patient allocation will be maintained by EnGeneIC. Investigators will inform EnGeneIC of potential patients, prior to a patient giving consent. If a spot is available it will be held for that potential patient. If the patient fails screening then that spot is made available to the next patient identified. Weekly status reports will be provided to all investigators and EnGeneIC.

A Safety Committee (SC) will be convened for the purpose of overseeing the dose escalation process and reviewing toxicity. The committee will consist of at least the principal investigators from each site, representative from EnGeneIC and an independent clinician with experience in conducting phase 1 studies. When the last patient in a cohort completes the first cycle, the SC will assess whether the dose should be escalated to the next level. The prescribed dose increases in Table 1 may be modified if recommended by the SC on review of emerging safety data. The SC may be convened on an ad hoc basis if required by the incidence of dose limiting toxicities.

6.3 Dose Escalation Rules

Table 2: Dose Escalation Rules

Number of Patients with DLT at Given Dose Level	Escalation Decision Rule
0 out of 3	Enter 3 patients at the next dose level
≥ 2	Dose escalation will be stopped. This dose level will be declared the maximally administered dose (highest dose administered). Three (3) additional patients will be entered at the next lowest dose level if only 3 patients were treated previously at that dose.
1 out of 3	Enter 3 more patients at this dose level to include a total of 6 patients. If 0 of these 3 patients experience DLT, proceed to the next dose level.
2 out of 6	Dose escalation is stopped, and this dose is declared the maximally administered dose. Three (3) additional patients will be entered at the next lowest dose level if only 3 patients were treated previously at that dose.
≤1 out of 6 at highest dose level below the maximally administered dose	This is generally the recommended phase 2 dose. At least 6 patients must be entered at the recommended phase 2 dose.

CONFIDENTIAL

6.4 Definition of Dose-Limiting Toxicity

All toxicities or adverse events will be graded according to the Common Toxicity Criteria for Adverse Events (CTCAE) Version 3. A dose limiting toxicity (DLT) is a toxicity or adverse event occurring in the first cycle, which is attributable to the study treatment and meets one of the following criteria:

- Clinically significant Grade 3 or 4, non-hematologic toxicity (including allergic reaction) except:
    - nausea and vomiting (NB treatment related grade 3 or 4 nausea or vomiting that persists for greater than 7 days despite medical management will be considered a DLT)
    - fever (in the absence of neutropenia)
    - asymptomatic hyperglycaemia
    - asymptomatic hyperuricaemia of any duration
    - biochemical abnormalities that resolve to grade 2 or better in 7 or less days.

  Clinically significant grade 3 or 4 biochemical abnormalities that persist (at grade 3 or above) for more than 7 days will be considered DLTs.

- Haematological toxicities
    - Febrile neutropenia (ANC < 1 X 10^9/L and fever > 38.5˚C)
    - Grade 4 neutropenia (ANC < 0.5 X 10^9/L) for 7 or more days
    - Grade 3 thrombocytopenia with bleeding or Grade 4 thrombocytopenia for 7 or more days

Resumption of study treatment for patients experiencing DLTs is permitted, contingent on the return of the DLT to ≤ Grade 1 severity and interruption or delay of treatment for no more than 3 weeks. Resumption of treatment after resolution of a DLT will be at the next lower dose level tested (or 50% lower if the DLT occurs with the first dose level).

6.5 Duration of Therapy

If the patient's tumour has remained stable or demonstrated at least partial response after completing a cycle of treatment (5 doses in 6 weeks), and providing there are no safety concerns, the patient may continue to receive further treatment cycles.

If the patient's tumour has progressed and they have no further proven treatment options they may continue to receive further treatment cycles provided:

- there are no unacceptable safety concerns

- the patient continues to have adequate organ function as defined in section 4.1.6
- the patient is willing to continue

6.6 Pre-medication

All patients should be pre-medicated before administration of $^{Erbitux®}$EDVs$_{Pac}$ using the following:

- Dexamethasone 8 mg i.v. at 30 minutes before $^{Erbitux®}$EDVs$_{Pac}$ infusion.
- Promethazine 12.5 - 25 mg i.v. at 30 minutes before $^{Erbitux®}$EDVs$_{Pac}$ infusion **OR** Promethazine 25 mg orally at 60 minutes before $^{Erbitux®}$EDVs$_{Pac}$ infusion **OR** Loratidine 20mg orally at 60 minutes before $^{Erbitux®}$EDVs$_{Pac}$ infusion Paracetamol 1g orally at 60 minutes before $^{Erbitux®}$EDVs$_{Pac}$ infusion

6.7 Management of Toxicity

Modifications for Events Occurring During or Immediately After $^{Erbitux®}$EDVs$_{Pac}$ Infusion

There is a theoretical potential for the occurrence of allergic-type reactions during and immediately following the administration of $^{Erbitux®}$EDVs$_{Pac}$. Patients will therefore be pre-treated with an appropriate antihistamine and corticosteroid before each infusion. As a routine precaution, patients enrolled into this study should be observed closely for any potential adverse effects. A medical practitioner will be available during the administration of the dose and throughout the observation period. Patients should be observed in an area with access to resuscitation equipment and agents (adrenaline, corticosteroids etc). Close monitoring of vital signs of the patient will take place during this period as outlined in the Schedule of Assessments in Appendix A.

Grade 3 or 4 allergic / hypersensitivity reactions require immediate interruption of the $^{Erbitux®}$EDVs$_{Pac}$ infusion, appropriate medical measures and permanent discontinuation of treatment. Patients should be carefully monitored until the resolution of signs and symptoms.

Management of allergic/hypersensitivity reactions during or post $^{Erbitux®}$EDVs$_{Pac}$ infusion:

CTC Grade Allergic/hypersensitivity reaction	Treatment
**Grade 1**	
Transient rash,	Decrease infusion rate by 50% and monitor closely for any worsening.

**Grade 2**<br>Urticaria, drug fever ≥ 38˚C and/or asymptomatic bronchospasm	Stop infusion.<br><br>Administer bronchodilators, oxygen etc as medically indicated<br><br>Resume infusion at 50% or previous rate once allergic/hypersensitivity reaction has resolved or decreased to Grade 1 in severity, and monitor closely for any worsening

CTC Grade Allergic/hypersensitivity reaction	Treatment
**Grade 3 or 4**<br><br>Grade 3: symptomatic bronchospasms requiring parenteral medication with or without urticaria; hypersensitivity related oedema, angioedema<br><br>Grade 4: anaphylaxis	Stop infusion immediately and disconnect tubing from patient.<br><br>Administer adrenaline, bronchodilators, antihistamines, glucocorticoids, intravenous fluids, vasopressor agents, oxygen, etc as medically indicated.<br><br>Patients must be withdrawn from treatment and not receive any further Erbitux® $EDVs_{Pac}$ doses. Patients should be followed up until resolution of the event and return for follow-up safety visits according to the study schedule (Appendix A)

- Appropriate therapeutic interventions may be initiated at any time.
- Patients experiencing Grade 1 or 2 reactions will not be prohibited from receiving subsequent Erbitux® $EDVs_{Pac}$ infusions.
- Patients will be **removed** from study for allergic reactions with a severity of ≥ Grade 3.

Management of febrile reaction post infusion
Febrile reactions are a possible consequence of cytokine release after the Erbitux® $EDVs_{Pac}$ infusion**.** These may be managed by observation alone. Paracetamol may be used if required.

Modifications for Events Occurring Between Erbitux® $EDVs_{Pac}$ Infusions

Patients, who experience Grade 3 toxicities between infusions, will not receive their next infusion of Erbitux® $EDVs_{Pac}$ until the toxicity improves to Grade 1. Patients will be removed from study if their toxicity does not improve to Grade 1 within three weeks. Patients will miss intra-cycle study doses (i.e. within the same cycle) that are scheduled during this recovery period. However, initiation of subsequent cycles may be delayed up to a maximum of 3 weeks to allow patients to recover.

6.7.1 EnGeneIC Delivery Vehicle Toxicity

Toxicity of the EDV is unknown in humans but any toxicity would be expected to be related to free endotoxin. In dogs which received an EDV preparation where the free endotoxin was measured at > 20 EU/10^9 EDVs, some animals showed a 0.5^0C to 1.0^0C temperature spike at 4 hours and occasionally vomited. This was self- limiting and temperatures returned to normal within 16 hours. Monkeys did not show this temperature spike, nor vomiting at doses of EDVs where the free endotoxin was kept below 20 EU per dose.

6.7.2 Cetuximab Toxicity

In this study, patients will be receiving <2µg of cetuximab per dose (compared with 400,000µg per kg conventional dosing with cetuximab).

The most common adverse events with conventionally dosed cetuximab (incidence ≥ 25%) are cutaneous adverse reactions (including rash, pruritis and nail changes), headache, diarrhoea and infection.

The most serious adverse events with cetuximab are infusion reactions, cardiopulmonary arrest, dermatologic toxicity and radiation dermatitis, sepsis, renal failure, interstitial lung disease and pulmonary embolism.

Serious infusion reactions included rapid onset airway obstruction, hypotension, shock, loss of consciousness, myocardial infarction and/or cardiac arrest. Severe infusion reactions occurred in 2-5% of 1373 patients in clinical trials with fatal outcome in 1 patient.

It is recommended that patients be monitored for an hour following cetuximab infusion in a setting with resuscitation equipment and other agents necessary to treat anaphylaxis.

Cardiopulmonary arrest and/or sudden death occurred in 2% of 208 patients treated with cetuximab in combination with radiation in for squamous cell carcinoma of the head and neck. Monitoring of serum electrolytes during and after cetuximab treatment is required.

Interstitial lung disease, including 1 fatality, occurred in 4 of 1570 (0.5%) patients receiving cetuximab in clinical trials. It is recommended to interrupt cetuximab for acute onset or worsening pulmonary symptoms and to permanently discontinue treatment for confirmed interstitial ling disease.

Acneiform rash occurred in 76-88% of 1373 patients receiving cetuximab in clinical trials (severe in 1-17% or patients). The rash resolved in the majority

of patients after cessation of treatment. It is recommended for patients receiving cetuximab to be monitored for dermatological toxicities and infectious sequelae and to instruct patients to limit sun exposure during treatment. For more severe skin toxicity treatment with a topical steroid (e.g., 1% hydrocortisone) may be considered.

Hypomagnesaemia occurred in 55% of patients (199/365) receiving cetuximab and was severe in 6-17%. Onset occurred days to months after initiation of treatment. It is recommended that patients receiving cetuximab be monitored for hypomagnesaemia, hypocalcaemia and hypokalaemia during and for at least 8 weeks following the completion of cetuximab.

6.7.3 Paclitaxel Toxicity

The quantity of paclitaxel administered to patients in this study is several orders of magnitude less than normal practice. The recommended dose of paclitaxel is in the range of 135-175 mg/m^2. For a patient with a body surface area of 1.7 m^2 this amounts to approximately 230 –300 mg of paclitaxel being administered. In this study, patients will receive between 0.00045 – 0.09 mg of paclitaxel which, at the highest dose level, is 2000-fold less than that delivered with conventional dosing. Although likely to be far better tolerated, there is no data available on the administration of paclitaxel at the low doses used in this trial.

After dosing with conventional dose paclitaxel, the most common adverse events (occurring in >50% of patients receiving single agent paclitaxel) are myelosuppression, reversible alopecia, peripheral neuropathy, myalgia/arthralgia and nausea/vomiting. Other less commonly reported adverse events include hypersensitivity reactions, infections, bleeding, diarrhoea, mucositis, elevated liver function tests, reactions at injection site and cardiovascular effects including hypotension, bradycardia, syncope, rhythm abnormalities, venous thrombosis and hypertension. Anaphylaxis and severe hypersensitivity reactions have occurred in 2-4% of patients receiving paclitaxel in clinical trials. Fatal reactions have occurred despite premedication.

Any patient who suffers an anaphylactic or severe hypersensitivity reaction (≥ grade 2) to the study medication will discontinue study treatment. They will be required to attend the safety follow-up visits according to the study schedule (Appendix A).

6.8 Concomitant Medication and Supportive Care Guidelines

As there is a potential for interaction of Paclitaxel with other concomitantly administered drugs through the cytochrome P450 system, the case report form

must capture the concurrent use of all other drugs, over-the-counter medications, or alternative therapies. The investigator should be alerted if the patient is taking any agent known to affect or with the potential to affect selected CYP450 isoenzymes. (Appendix F)

Potent inhibitors of CYP3A4 and 2C8 should preferably be discontinued prior to treatment in the study, unless considered medically inadvisable by the investigator. In these patients caution should also be exercised, and patients monitored closely.

7 STUDY PROCEDURES

This is an open label and uncontrolled multicentre phase I study. Refer to the Schedule of Assessments (Appendix A) for an outline of the procedures required at each visit.

7.1 Screening

Once a patient has personally signed and dated the HREC approved patient information sheet and consent form, study specific screening procedures may commence. Procedures that are part of routine care may be used to determine eligibility if they comply with the study's requirements.

All patients must have the following procedures completed within 21 days before their first dose:

- Review and documentation of inclusion and exclusion criteria
- Medical and medication history
- Recording of all concurrent medication and treatments
- Height and weight,
- Resting pulse, respiration, blood pressure, temperature,
- ECOG performance status
- Physical examination including neurological assessment
- 12-lead ECG taken after the patient has been supine for at least 5 minutes
- Radiological imaging by CT scans of all sites of disease to assess disease extent. Disease assessment will be made according to RECIST (Appendix E).
    - In **part 2 of the study** a FDG PET scan will also be performed.
- Test for presence and level of antibodies to salmonella species and Erbitux®
- EGFR Status – in **part 1 of the study** this is only required for those patients presenting with tumours not commonly known to express EGFR.
    - In **part 2 of the study**, all patients, irrespective of the presenting tumour type, must have documented evidence confirming positive EGFR status. Those patients for whom this is not already documented will have a recent or archived biopsy sample tested to confirm their EGFR status prior to treatment.

All patients must have the following procedures completed within 10 days before their first dose:

- Laboratory tests: blood chemistry, full blood count, coagulation, calculated creatinine clearance, urinalysis; serum pregnancy test

7.2 Treatment Phase

A cycle consists of the 5 infusions administered at weekly intervals, followed by a treatment free week. Therefore, the first dose of a cycle is administered 6 weeks after the first dose of the previous cycle. If necessary, the first dose of a cycle may be delayed by up to 3 weeks to allow a patient to recover from adverse events.

Patients will be seen and will have study medication administered on days 1, 8, 15, 22 and 29 of each cycle. There should be a minimum of 5 days and a maximum of 9 days between treatments. However, as much as possible the gap between doses should be kept to 7 days.

On each treatment day in cycle 1, patients will remain at the clinic for a minimum of 6 hours after dose administration. Provided there are no safety concerns they will be permitted to go home but will return to the clinic at 24 hours after dose administration for blood tests and an ECG.

On each treatment day from cycle 2 onwards, patients will remain at the clinic for a minimum of 4 hours after dose administration. Provided there are no safety concerns they will be permitted to go home and no further assessments will be required.

Before the dose is administered at each treatment visit, the following procedures will be performed:

- Recording of adverse events and changes to adverse events
- Recording of changes to concomitant medication

- Weight
- Resting pulse, respiration, blood pressure and temperature
- ECOG performance status
- Physical examination including neurological assessment
- 12-lead ECG
- Laboratory tests: blood chemistry, full blood count, coagulation, calculated creatinine clearance, urinalysis
- Blood sample for pharmacokinetic, immune and inflammatory response analyses and antibodies to salmonella species and Erbitux®

At **1, 2, 4, 6 and 24 hours** after the dose the following procedures will be performed:

- Recording of adverse events and changes to adverse events
- Recording of changes to concomitant medication
- Resting pulse, respiration, blood pressure and temperature. If an elevated temperature is recorded it should be measured at least 4 hourly (if supplementary to scheduled assessments) until it returns to normal

At **4 and 24 hours** after the dose the following procedures will also be performed:

- 12-lead ECG
- Laboratory tests: blood chemistry, full blood count, coagulation, calculated creatinine clearance, urinalysis
- Blood sample for pharmacokinetic analysis, immune and inflammatory response analyses

Note 1: the 6 and 24 hour observations will only be performed for the patients' first cycle in the study.

Note 2: pharmacokinetic analysis will only be conducted on samples from the patients' first cycle in the study.

7.3 Safety Follow Up

All patients must have the following procedures completed 30-35 days after the administration of their last dose:

- Recording of adverse events and changes to adverse events
- Recording of changes to concomitant medication

- Weight
- Resting pulse, respiration, blood pressure and temperature
- ECOG performance status
- Physical examination including neurological assessment
- 12-lead ECG
- Laboratory tests: blood chemistry, full blood count, coagulation, calculated creatinine clearance, urinalysis
- Blood sample for antibodies to salmonella species and Erbitux®

For patients who discontinue the study before completion of the first cycle, an additional follow-up visit (including all the same procedures) should be conducted within 14 days after the administration of the last dose.

7.4 Specific Procedures

7.4.1 Height and weight

Height and weight will be measured in light-weight clothing, without shoes.

7.4.2 Vital signs: Resting Pulse, Respiration, Temperature, Blood Pressure

Blood pressure will be measured using the same arm throughout the study in sitting position after 5 minutes rest.

Pulse rate will be measured under the same conditions as the blood pressure measurements either manually by palpation of the radial pulse, or electronically.

Aural temperature will be measured electronically.

7.4.3 Physical Examination

Physical examinations will be performed by medically qualified personnel who are listed as study personnel.

The physical examination will include the neurological and dermatological assessments.

7.4.4 ECG

Twelve-lead ECGs will be performed and evaluated at each site by medically qualified personnel.

7.4.5 Standard Laboratory Assessments

Laboratory tests will be performed by the local laboratory.

Blood Chemistry will include sodium, potassium, chloride, bicarbonate, calcium, glucose, total bilirubin, alkaline phosphatase, ALT (SGPT), AST (SGOT), LDH, blood urea nitrogen (BUN), creatinine, magnesium, phosphate, cholesterol, triglycerides, albumin, total protein, uric acid and c-reactive protein

Full Blood Count will include haemoglobin, haematocrit, red blood cells, mean corpuscular volume, white blood cells, neutrophils, basophils, eosinophils, lymphocytes, monocytes, platelets and ANC

Coagulation tests will include PTT and INR

Calculated creatinine clearance will be calculated using the Cockcroft-Gault formula for patients with serum creatinine outside the normal range(see Appendix C)

Urinalysis will include a dipstick analysis of urine ph, blood, glucose, protein

Serum pregnancy test for women of child bearing potential

7.4.6 Salmonella and Erbitux® Antibodies

Blood samples will be sent to EnGeneIC Pty Ltd for a quantitative analysis of salmonella and Erbitux® antibody titres.

7.4.7 Pharmacokinetic Sample

Blood samples for pharmacokinetic analysis of paclitaxel will be taken at pre-dose, 4 and 24 hours post dose during the patients' first cycle in the study. Samples will be sent to EnGeneIC Pty Ltd for HPLC analysis.

7.4.8 Immune and Inflammatory Response

Blood samples for measurement of cytokines and inflammatory mediators, TNFα, IL-6, IFNα, IFNβ, IFNγ, IL-1β, IL-2, IL-4, IL-8, IL-10, IL-12p70 will be taken at pre-dose, 4 and 24 hours post dose. Samples will be sent to EnGeneIC Pty Ltd for analysis.

7.4.9 Radiological Assessment of Tumour

CT Scans

Screening/Baseline imaging will include CT scans of the known tumour sites of disease as well as scans of the chest, abdomen and pelvis if not already performed. MRI scans may be used for tumour assessment if felt to delineate disease better. Scans will be repeated after each 6 week cycle. The same method of assessment and the same technique should be used to characterise each identified lesion at baseline and during follow up. IV contrast should be used for all studies unless contraindicated. Disease responses will be categorised using RECIST criteria.

PET scans

FDG-PET scans will be performed at baseline (Day -21 to Day -1) and at Day 36-42 only in patients enrolled in part 2 of the study. Details of the FDG-PET will be provided in the ENG1 procedure manual at the time of study initiation.

7.4.10 Epidermal Growth Factor Receptor Immunohistochemistry

EGFR protein expression will be assessed by immunohistochemistry using the Dako EGFR PharmDx kits (DakoCytomation). Staining will be reviewed by a pathologist and tumours with more than 20 percent of tumour cells demonstrating membranous (partial or complete) staining of any intensity will be scored as positive.

8 WITHDRAWAL AND REPLACEMENT OF PATIENTS

Patients should discontinue the study treatment in the case of unacceptable toxicity.

Discontinuation from the study treatment does not constitute withdrawal from the study. Patients have the right to withdraw from the study at any time and for any reason without prejudice to their future care.

If a patient is withdrawn prematurely, whether due to an adverse event or not, the investigator should arrange for the patient to return for the follow-up safety visits at 30 days after the patient's last dose (within 14 days also if withdrawn before completion of the first cycle). Follow-up of ongoing adverse events beyond these visits is required until resolution or stabilisation of the events to a level acceptable to the investigator and EnGeneIC.

The Investigator can also discontinue the patient's study treatment if it is in the best interest of the patient.

Patient Replacement Policy

Patients in part 1 and part 2 of the study who do not provide adequate safety information up to and including the post-cycle CT and PET scans of the first cycle of dosing will be replaced. Unless, that is, toxicity experienced by other patients in the same cohort indicates that dosing should revert to a lower dosage level.

9 SAFETY REPORTING

9.1 Definitions

Adverse events

An adverse event is defined in the International Conference on Harmonisation (ICH) Guideline for Good Clinical Practice as "any untoward medical occurrence in a patient or clinical investigation subject administered a pharmaceutical product and which does not necessarily have to have a causal relationship with this treatment".

Any worsening of a pre-existing medical condition (e.g. diabetes, migraine headaches, arthritis, asthma etc) should be considered an adverse event if there is either an increase in severity, frequency or duration of the condition or an association with significantly worse outcomes.

Interventions for pre-treatment conditions (e.g. elective cosmetic surgery) or medical procedures that were planned prior to study enrolment are not considered adverse events.

The possibility that a pre-existing medication condition has been exacerbated or worsened should be considered whenever a patient requires new or additional concomitant drug or non-drug therapy for the treatment of that illness during the study.

Clinically significant changes from baseline values in physical examination findings and objective test findings (e.g. laboratory, ECG, x-ray and other imaging) should be recorded as adverse events. The criteria for determining whether an abnormal objective test finding should be reported as an adverse event are as follows:

1. The test result is associated with accompanying symptoms; and/or
2. The test requires additional diagnostic testing or medical/surgical intervention (this does not include merely repeating an abnormal test); and/or
3. The test leads to a change in study dosing or discontinuation from the study; and/or
4. The test result leads to any of the outcomes included in the definition of a serious adverse event; and/or
5. The test is considered to be an adverse event by the investigator or EnGeneIC.

   Any abnormal test that is determined to be an error does not require reporting as an adverse event.

Serious Adverse Events

A serious adverse event (SAE) is defined as an adverse event that:

- Is fatal
- Is life threatening (places the subject at immediate risk of death)
- Requires in-patient hospitalisation or prolongation of existing hospitalisation
- Results in persistent or significant disability/incapacity
- Is a congenital anomaly/birth defect

Important medical events that do not result in one of the above outcomes may still be considered serious adverse events if they require medical or surgical intervention to prevent these outcomes from occurring e.g. allergic bronchospasm requiring treatment in an emergency department or at home; convulsions and blood dyscrasias.

A hospitalisation meeting the regulatory definition for "serious" is any in-patient hospital admission that includes a minimum of an overnight stay in a health care facility. A visit to the emergency department, out-patient clinics or an urgent investigation do not meet the definition of hospitalisation but may still be considered serious at the discretion of the investigator if they result from a significant medical hazard. Exceptions to these definitions for serious adverse events include anticipated protocol-specified procedures such as overnight hospital stay for observation.

9.2 Adverse Event Reporting

The investigator is responsible for ensuring that all adverse events, as defined above, observed by the investigator or reported by the patient are properly captured in the patients' medical records and the CRF.

All adverse events that occur during the study period and through to the last follow-up visit or 30 days post the last dose of the investigational product will be recorded regardless of whether they are considered related to the investigational product.

For all adverse events, the investigator must obtain adequate information to determine the adverse event diagnosis or syndrome (or signs or symptoms if not known), event description, dates of onset and resolution (or other outcome), severity, causality and action taken. Follow-up of ongoing adverse events beyond completion or discontinuation of the study is required until resolution or stabilisation of the event to a level acceptable to the investigator and EnGeneIC.

9.3 Serious Adverse Event Reporting

Serious adverse events (SAE) will be collected, recorded and reported throughout the study period through to the last follow-up visit or 30 days post the last dose of investigational product whichever comes later. All serious adverse events (regardless of relationship to study drug) will be followed up until resolution.

The trial site must report any SAEs to Datapharm Australia within 24 hours of receiving initial notification of the event. The initial contact number is listed below.

**Pharmacovigilance at Datapharm Australia Pty Ltd**<br>**Telephone: 02 9719 2800**<br>**Fax: 02 9719 2811**<br>**Urgent After Hours Contact: 0408 206 115**

The report will normally be in the form of a telephone call or a faxed Serious Adverse Event Form. The minimum information that should be provided includes:

- Protocol number
- PI name and site number
- Subject number and initials
- Diagnosis (primary) of event, or symptoms, if diagnosis not available
- Start date of event and date event became serious, if different to start date
- Status of event (ongoing, resolved, etc.)
- Seriousness criteria
- Study treatment, if known
- Relationship to study drug
- Status of study drug (withdrawn, discontinued, unchanged, etc.)
- Initial or follow-up report
- Contact details of person reporting the event

Follow-up reports should be completed to report additional information as it becomes available. It is not necessary to complete a new document for each follow-up report. The new information can be added to the initial report provided the addition or amendment is clearly identifiable to the reviewer, i.e., all changes initialled and dated. Alternatively, if a large amount of information needs to be added, another form can be completed. In this case it will be necessary to complete the demographic information and patient identifiers on the new report, but not all the information that has been previously reported. The report should be reviewed, signed and dated, or in the case of a follow-up report, re-signed and dated by the Principal Investigator. The PI can delegate this responsibility to another investigator as appropriate.

If, during a monitoring visit, a Site Monitor identifies a SAE that the site has not reported to Datapharm Australia, the Site Monitor will instruct the site to complete the SAE report and submit to Datapharm Australia.

Any event classified as "serious" should be reported to the Human Research Ethics Committee (HREC) overseeing the study at the site where the event occurred according to the HREC's guidelines for SAE reporting. Investigators at Australian sites should also inform each HREC of:

- All serious or unexpected adverse events that occur during the trial and may affect the conduct of the trial or the safety of the participants or their willingness to continue participation in the trial;
- Any new information which may have an impact on the continued ethical acceptability of the trial or which may indicate the need for amendments to the trial protocol.

Any serious adverse event occurring beyond 30 days after the last administration of the investigational product must still be promptly reported if a causal relationship to the study drug is suspected.

For all deaths during the study up to 30 days post the last dose of investigational product, available autopsy reports and relevant medical reports should be made available to EnGeneIC.

Disease progression is not to be reported as an adverse event. Deaths on the study (or within 30 days after the last study dose of investigational product) where no acute events can be determined as the cause of death are to be reported but may be reported as a consequence of the underlying malignancy.

If a patient is permanently withdrawn from the study because of a serious adverse event, this information must be included in the patients' medical records, initial or follow-up Serious Adverse Event Report Form as well as the CRF.

10 INVESTIGATIONAL AGENT

10.1 Packaging and Formulation

$^{Erbitux®}$EDVs$_{Pac}$ has been assigned the code EDV1-1. It is presented as a sterile, lyophilised powder in single use vials. Prior to patient dosing, the appropriate vial will need to be reconstituted with sterile normal saline.

$^{Erbitux®}$EDVs$_{Pac}$ contains approximately 140 ± 10 µg of paclitaxel and 5 ± 0.5 µg of Erbitux® per 2 X 10^{11} EDVs.

The following table lists the quantity of $^{Erbitux®}$EDVs$_{Pac}$ in the starting vials for each dose level.

Dose level	Dose of $^{Erbitux®}$EDVs$_{Pac}$	Single Use Vial contents
1	1 x 10^{8} $^{Erbitux®}$EDVs$_{Pac}$	7.5 x 10^{8} $^{Erbitux®}$EDVs$_{Pac}$
2	1 x 10^{9} $^{Erbitux®}$EDVs$_{Pac}$	7.5 x 10^{9} $^{Erbitux®}$EDVs$_{Pac}$
3	1 x 10^{10} $^{Erbitux®}$EDVs$_{Pac}$	7.5 x 10^{10} $^{Erbitux®}$EDVs$_{Pac}$
4	5 x 10^{10} $^{Erbitux®}$EDVs$_{Pac}$	1.5 x 10^{11} $^{Erbitux®}$EDVs$_{Pac}$
5	1 x 10^{11} $^{Erbitux®}$EDVs$_{Pac}$	3 x 10^{11} $^{Erbitux®}$EDVs$_{Pac}$
6	2 x 10^{11} $^{Erbitux®}$EDVs$_{Pac}$	5 x 10^{11} $^{Erbitux®}$EDVs$_{Pac}$

10.2 Labelling

The study medication will be labelled in accordance with Australian requirements.

10.3 Storage

$^{Erbitux®}$EDVs$_{Pac}$ should be stored between 2-8^{0}C and protected from light.

The $^{Erbitux®}$EDVs$_{Pac}$ should NOT FREEZE.

Unnecessary agitation must be avoided.

10.4 Preparation and Dispensing

Individual patient doses may be reconstituted and prepared up to 24 hours prior to administration and stored between 2-8^{0}C until required.

Personnel responsible for the preparation and dispensing of the investigational product will receive appropriate training. Detailed procedures and necessary equipment will be provided by EnGeneIC.

Individual doses will be prepared in aseptic conditions and will involve the following steps:

1. Lyophilised powder will be reconstituted using sterile normal saline.

2. Since the EDVs are of bacterial origin, some EDVs will break up post-lyophilisation and reconstitution. Therefore the debris must be removed prior to patient dosing. Vivaspin 500 (for dose levels 1 to 3) and Vivaspin 20 (for dose levels 4 to 6) are used to clean up the EDVs. These are sterile disposable filtration units which carry a cross-flow filtration membrane with a pore size of 0.2 µm. The reconstituted EDV suspension from step 1 is placed in the appropriate Vivaspin filtration device (which is sealed to allow removal from aseptic preparation conditions), centrifuged to eliminate the debris and the clean suspension of EDVs is recovered.

3. A sample of the purified EDVs is used to measure the optical density in a spectrophotometer. The optical density value is then interpolated in a graph (will be provided) to determine the concentration of the EDVs in the suspension.

4. Another small sample is placed in a portable endotoxin measurement device (will be provided) to determine the level of purity of the EDV suspension. Each EDV dose should carry less than 20 endotoxin units (EU). In the unlikely event that the dose contains more than 20 EU it should be discarded and a new dose prepared using a fresh vial.

5. The appropriate number of EDVs required for the specific dose level would then be removed and diluted in 20 mL normal saline ready for infusion. The remaining sample will be placed in a fridge and stored. Once every two weeks, these stored samples would be sent to EnGeneIC for further quality control analyses

A fresh Erbitux® EDVs$_{Pac}$ vial will be used for each patient dose.

10.5 Supply and Return of Investigational Agent

At study initiation and as needed thereafter, study medication kits will be sent to the responsible pharmacist at each site, who will check the amount and condition of the medication and confirm receipt to EnGeneIC. At the end of the study, or as directed, all study medication including all used as well as unused study medication kits will be returned to EnGeneIC.

10.6 Accountability

Accountability records for the study medication must be kept current and must contain:

- Dates and quantities of study medication received from EnGeneIC
- Patient identification numbers used
- Dose preparation records
- Date study doses prepared and dispensed
- Initials of dispenser
- Quantity of dose returned to pharmacy – if any
- Dates and quantities of study medication returned to EnGeneIC

11 DATA ANALYSIS AND STATISTICAL METHODS

11.1 Sample Size Determination

The following table outlines the probability of at least two patients experiencing DLT at a given dose for a range of underlying rates of DLT. With the escalation scheme in this trial, there would be more than an 80% chance that at least two patients would experience DLT for a given dose, if the underlying probability of DLT were at least 50% for each patient.

	Underlying Probability of a Patient Developing of Dose Limiting Toxicity			
	20%	40%	50%	60%
Probability of Observing at Least Two Patients with DLT for a Given Dose	0.29	0.69	0.83	0.92

It is estimated that 20 patients with immunohistochemically confirmed EGFR expression and measurable disease will be enrolled onto the recommended phase 2 dose expansion part of the study. Based on a single stage design (Fleming, 1982) for response (H_0: $p \leq 0.10$; H_A: $p \geq 0.40$, alpha =5% and power ≥ 90%), the observation of at least 5 responders in 20 patients in this cohort provided at least 95% confidence that the true rate is greater than 10%. A response rate of greater than 10% in an advanced population of cancer patients would indicate the presence of clinically meaningful anti-tumour activity.

11.2 Methods of Analysis

Due to the small number of patients to be enrolled in each dose group, findings will be presented in a descriptive manner and no formal statistical comparisons will be performed. Continuous data will be summarised by the following

descriptive statistics: n (number of observations), mean, standard deviation, median, minimum, maximum. Categorical data will be summarised by frequencies and percentages. The 95% confidence intervals will be provided.

A detailed statistical analysis plan (SAP) will be compiled prior to data base lock.

11.2.1 Safety Data

The following safety outcomes will be presented:

- Adverse events
- Serious adverse events
- Dose-limiting toxicities
- Clinically significant laboratory findings (including immune and inflammatory responses)

The safety population will consist of all enrolled patients who receive at least one dose of study medication. Patients who are removed from the study prior to completing the first cycle, for reasons other than dose-limiting toxicities (DLT), will be replaced (see Section 8).

A treatment-emergent adverse event (TEAE) is defined as an AE that starts after administration of study medication or that was seen prior to the start of study drug administration but increased in severity during treatment. TEAEs will be listed individually and tabulated by System Organ Class (SOC), Preferred term (PT). In addition, TEAEs will be tabulated by severity and relationship to study drug, per cycle and dosage group. Serious adverse events (SAEs) will be listed using the following information: start date and time, stop date and time, maximum severity, causality, treatment action, outcome and reason for SAE. All reported adverse events will be coded using MedDRA®.

The MTD will be identified as per definition in Section 6.3.

DLT (see Section 6.4) will be presented per patient, dosage group and cycle number. The action taken for each DLT will also be presented. Trends between toxicities and increasing doses will be evaluated. Toxicities leading to Serious Adverse Events (SAEs) will be presented for each patient by dosage group and cycle number.

Clinically significant changes in vital sign variables (including significant changes in temperature), 12-lead ECG variables and physical examination variables will be listed at each applicable protocol visit by dosage group.

Laboratory variables (blood chemistry, full blood count, coagulation, calculated creatinine clearance, urinalysis, immune and inflammatory response, salmonella and Erbitux® antibodies) will be summarized at each protocol visit by dosage group.

In addition, laboratory values will be compared to the normal ranges of the applicable laboratory, for each of the dosage groups. Laboratory values that fall out of the normal ranges will be listed as: H (high), L (low) and furthermore, it will be indicated whether each out of range value is CS (Clinically significant) or NCS (Not Clinically Significant). Laboratory values will be investigated further by calculating the changes from baseline. The mean change from baseline values, for each variable, will be presented descriptively.

11.2.2 Pharmacokinetic (PK) Data

If paclitaxel is detectable in the blood, the following pharmacokinetic variables will be calculated using validated pharmacokinetic software:

- the maximum serum concentration (C_{max}),
- time of maximum serum concentration (T_{max}),
- area under the serum concentration-time curve up to the last quantifiable concentration (AUC (0-t_{last})),
- area under the serum concentration-time curve extrapolated to infinity (AUC(0-∞),
- terminal rate constant (λ_z)
- terminal elimination half-life ($t_{1/2}$)
- total serum clearance (CL)
- mean residence time (MRT)
- apparent volume of distribution during the terminal phase (V_z)
- volume of distribution at steady-state (V_{ss})

The PK data will be summarised by dosage group.

11.2.3 Efficacy Data

The following efficacy outcomes will be presented:

- Evidence of anti-tumour activity using CT scan, as per RECIST criteria

  The best overall tumour response to treatment

  The number of patients categorised to have a best overall response as: complete response, partial response, stable disease, progressive disease, will be presented by dosage group and overall, using frequency counts and percentages.

  The overall response of each patient will be recorded at each protocol specific visit where disease assessment is evaluated, although it is noted that according to the RECIST criteria, to be assigned a status of complete or partial response, changes in tumour measurements must be confirmed

by repeat assessments that should be performed no less than four weeks after the criteria for response are first met.

- Evidence of anti-tumour activity using FDG-PET scan (Part 2, first cycle)

  The proportion of patients that have a >20% reduction in Standardised Uptake Volume (SUV) from baseline

  FDG PET scans are performed in part 2 only at baseline and at the end of the first cycle. These scans will be compared qualitatively and quantitatively (i.e. the change and percentage change in SUV from baseline will be calculated.) Patients are considered to have a partial metabolic response if there is a > 20% reduction in SUV from baseline. (Young *et al.*, 1999) The proportion of patients with a partial metabolic response will be defined and the 95% confidence interval stated.

12 ADMINISTRATIVE, REGULATORY AND LEGAL OBLIGATIONS

12.1 Compliance with Good Clinical Practice and Ethical Considerations

This study will be conducted according to Australian and international standards of Good Clinical Practice [*The National Health and Medical Research Council's National Statement on Ethical Conduct in Human Research (2007) and the Note for Guidance on Good Clinical Practice (CPMP/ICH/135/95) annotated with TGA comments (2000)].* Applicable government regulations and Institutional research policies and procedures will also be followed.

12.2 Informed Consent

All subjects for this study will be provided a consent form describing this study and providing sufficient information for subjects to make an informed decision about their participation in this study. This consent form will be submitted with the protocol for review and approval by the HREC.

The formal consent of a patient, using the HREC-approved consent form, will be obtained before that patient is submitted to any study procedure. The investigator, or a research professional designated by the investigator, will explain the aims, methods, potential benefits and risks of participation in the study to each patient. Once satisfied the patient has made an informed decision, without any form of coercion, the consent form must be signed and personally dated by the patient, and the investigator or designated research professional obtaining the consent. The original signed informed consent form will be retained by the investigator and a copy (or a second original) will be provided to the patient.

With the patient's consent to do so, the investigator is responsible for informing the patient's local doctor of the patient's participation in the study.

Acquisition of informed consent must be documented in the patient's medical records including whether the patient consented to their local doctor being informed of their participation.

12.3 Human Research Ethics Committee Review

A copy of the protocol, proposed informed consent and any other written information for the patients must be submitted to a HREC (that is constituted and operates in accordance with ICH GCP and all applicable laws and regulation) for written approval. A copy of the written approval must be received by EnGeneIC, or their agents, prior to recruitment of the first patient and shipment of the investigational product.

The investigator must submit and obtain approval from the HREC for all subsequent protocol amendments and changes to the informed consent document. The investigator must also notify the HREC of deviations from the protocol and SAEs that occur during the study.

The investigator is responsible for fulfilling all the reporting obligations as required by the HREC.

12.4 Pre-study Documentation Requirements

The investigator is responsible for forwarding the following documents to EnGeneIC prior to the commencement of the:

- A protocol signed and dated by the investigator
- Copy of the approved informed consent form
- Copy of the HREC approval letter of the protocol and informed consent form in accordance with the National Statement on Ethics Conduct in Human Research and ICH GCP (TGA annotated) requirements
- HREC composition or a written statement that the HREC is in compliance with the National Statement on Ethics Conduct in Human Research
- Current CV of principal investigator and all co/sub-investigators
- Laboratory normal ranges and documentation of laboratory certification
- A signed clinical trial agreement

12.5 Patient Confidentiality

The investigator will ensure that the patient's confidentiality is maintained. On the CRF or any documentation that is submitted to EnGeneIC, patients must be identified by the study identification numbers and initials only.

Any information reported or published as a result of the study will not permit identification of any patient.

12.6 Protocol Amendments

Protocol amendments, except where necessary to alleviate an immediate hazard to the patient, must be made only with prior approval of EnGeneIC and agreement of the investigator

The investigator is responsible for submitting any amendments to the approved protocol in writing to the HREC for review and approval and forwarding a copy of the approval to EnGeneIC prior to implementing the amendments.

12.7 Study Termination

Both EnGeneIC and the investigator reserve the right to terminate the study according to the study contract. The investigator must notify the HREC in writing of the study's completion or early termination and send a copy of the notification to EnGeneIC.

Recruitment may be stopped at a centre for particularly low recruitment, protocol violation or inadequate data recording.

Patients may be eligible for continued treatment with the investigational product by extension protocol. However, EnGeneIC reserves the right, at its discretion, to determine whether to supply the investigational product, and by what mechanism, after termination of the trial and before it is available commercially.

12.8 Monitoring

An EnGeneIC monitor, or appointed agent, is responsible for contacting and visiting the investigator periodically to discuss the progress of the study and to verify the adherence to the protocol, ICH GCP and regulatory requirements. This will involve review of the CRFs and source documents for completeness and accuracy of data recording; review of investigational product accountability records, review of correspondence and inspection of facilities. The monitor must have access to the patients' medical records and other study related records needed to verify the entries in the CRF. The monitor will ensure that the patient's confidentiality is respected.

The investigator agrees to cooperate with the monitor to ensure that any problems detected in the course of these monitoring visits are resolved.

CONFIDENTIAL

Periodically some or all of the facilities used in the study (e.g. pharmacy, drug storage areas, laboratories etc) may be reviewed or inspected by the HREC, regulatory authorities and/or auditors appointed by EnGeneIC for clinical quality assurance. In compliance with ICG GCP (TGA annotated) it is required that the investigator and institution permit authorised representatives of EnGeneIC, regulatory agencies and the HREC direct access to review the patient's original medical records for verification of study related procedures and data.

The investigator is to ensure that the patients' are aware of and consent that personal information may be reviewed during these monitoring and inspection/audit processes.

12.9 Data Collection

In compliance with ICH GCP, the medical records should be clearly marked and permit easy identification of participation by the patient in the study. In some cases, with prior agreement of EnGeneIC, data may be recorded directly onto a CRF instead of the medical records and will be considered the source data.

The investigator is to record all data with respect to protocol procedures, drug administration, laboratory data, safety data and efficacy data using CRFs or other collection methods designed by EnGeneIC. The investigator will sign to attest the accuracy and completeness of the data.

All corrections on a CRF and on source documents must be made in a way that does not obscure the original entry, such as a single line stroke through the error and the insertion of the correction above or beside the error. The correction must be initialled and dated by the investigator or designee identified on the Delegation of authority form. There should be no erasures or covering of data with correction fluid or tape.

12.10 Study Documentation and Archiving

Source documents are original documents, data, and records from which the patients CRF data are obtained. These include but are not limited to hospital records, clinical and office notes/charts, laboratory and pharmacy records, diaries, microfiches, imaging films and correspondence.

The investigator and study staff are responsible for maintaining a comprehensive and centralised filing system of all study related (essential) documentation, suitable for inspection at any time by representatives from EnGeneIC, HRECs and/or regulatory authorities. All source document supporting data in the CRFs must be maintained and be readily available.

All study related documentation must be retained for a minimum of 15 years following completion of the study. Even beyond this time, no study related documentation should be destroyed or discarded without prior written agreement between EnGeneIC and the investigator. Should the investigator need to assign the study records to another party or move then to another location, he/she must notify EnGeneIC in writing of the new responsible person and/or new location and provide for the continuing access to the records by EnGeneIC personnel and regulatory authorities.

12.11 Study Reporting and Publication Policy

Data analysis, statistical reporting and report preparation will be the responsibility of EnGeneIC or their appointed agent.

EnGeneIC will write up the study for publication. Authorship on the paper will include relevant EnGeneIC staff and the Investigators from each site.

12.12 Compensation

Subjects will be treated and/or compensated for any study-related illness/injury as outlined in the information provided in the Informed Consent documents.

13 REFERENCES

Abraxane Drug Information. **Food and Drug Administration.** January 7, 2005.

Cunningham D, Humblet Y, Siena S, et al.(2004) Cetuximab monotherapy and cetuximab plus irinotecan in irinotecan-refractory metastatic colorectal cancer. **N Engl J Med** *351*:337–345

Desai N, Trieu V, Yao Z, et al. (2006) Increased antitumour activity, intratumour paclitaxel concentrations, and endothelial cell transport of Cremophor®-free, albumin-bound paclitaxel, ABI-007, compared with Cremophor-based paclitaxel. **Clin Cancer Res***, 12*:1317-1324.

Fleming TR (1982). One-sample multiple testing procedure for phase II clinical trials. **Biometrics**. Vol 38, No 1, pp 143-151

Foa, R., Norton, L., and Seidman, A.D. (1994). Paclitaxel: A novel anti-microtubule agent with remarkable anti-neoplastic activity. **J.Biol.Chem**., *24 (1)*, 6-10.

Fosmire SP, Dickerson EB, Scott AM, Bianco SR, Pettengill MJ, Meylemans H, Padilla M, Frazer-Abel AA, Akhtar N, Getzy DM, Wojcieszyn J, Breen M, Helfand SC, Modiano JF. (2004). Canine malignant hemangiosarcoma as a model of primitive angiogenic endothelium ***Laboratory Investigation*** **84:** 562–572 .

Gao, X., Cui, Y., Levenson, R.M., Chung, L.W., Nie, S. (2004). *In vivo* cancer targeting and imaging with semiconductor quantum dots. **Nat. Biotechnol**. *22,* 969–76.

Gradishar WJ, Tjulandin S, Davidson N, et al. (2005). Phase III trial of nanoparticle albumin-bound paclitaxel compared with polyethylated castor oil-based paclitaxel in women with breast cancer. **J Clin Oncol**. *23*:7794-7803.

Hobbs, S.K., Monsky, W.L., Yuan, F., Roberts, W.G., Griffith, L., Torchilin, V.P., Jain, R.K. (1998). Regulation of transport pathways in tumour vessels: Role of tumour type and microenvironment. **Proc. Natl Acad. Sci. USA** *95,* 4607–4612.

Holbro T, Civenni G, Hynes NE (2003) The ErbB receptors and their role in cancer progression. **Exp Cell Res** *284*:99–110

Kumar, N. (1981) Taxol-induced Polymerization of Purified Tubulin. **J. Biol. Chem**.*256*(20), 10435-10441.

Lockhart C, Berlin JD (2005) The epidermal growth factor receptor as a target for colorectal cancer therapy. **Semin Onco**l *32*:52–60

Lenz, H.J., Van Custem,E., Kabarta-Ford, S., et al. 2006. Mutlicentre PhaseII and translational study of cetuximab in metastatic colorectal carcinoma refractory to irinotecan, oxaliplatin and fluropyrimidines.**J.Clin.Oncol**. *24*:4914-21

Ma, L., King, G.F., Rothfield, L. (2004). Positioning of the MinE binding site on the MinD surface suggests a plausible mechanism for activation of the *Escherichia coli* MinD ATPase during division site selection. **Mol. Microbiol**. *54,* 99–108.

MacDiarmid, J.A., Mugridge, N.B., Weiss, J,C., et al 2007 . Bacterially-derived 400nm particles for encapsulation and cancer cell targeting of chemotherapeutics. **Cancer Cell**. *11:* 431-445.

MacDiarmid, J.A., Madrid-Weiss, J.C., Amaro-Mugridge, N,B. Phillips, L., and Brahmbhatt, H. (2007). Bacterially-derived nanocells for tumour-targeted delivery of novel chemotherapeutics and cell cycle inhibitors. **Cell Cycle** *6:* 1-7.

Nikaido H. (2003). Molecular basis of bacterial outer membrane permeability revisited. **Microbiol. Mol. Biol. Rev***. 67,* 593–656.

Nielsen, U.B., Kirpotin, D.B., Pickering, E.M., Hong, K., Park, J.W., Refaat Shalaby, M., Shao, Y., Benz, C.C., Marks, J.D. (2002). Therapeutic efficacy of anti-ErbB2 immunoliposomes targeted by a phage antibody selected for cellular endocytosis. **Biochim. Biophys. Acta**. *1591,* 109–118.

Park, J.W. (2002). Liposome-based drug delivery in breast cancer treatment. **Breast Cancer Res.** *4,* 95–99.

Schultz, G.E. (1993). Bacterial porins: Structure and function. **Curr.Opin.Cell Biol.** *5*:701-707.

Taxol® (paclitaxel) Injection **Product Information**, Revised July 2007

Van den Berg, B. (2005). TheFadL family: Unusual transporters for unusual substrates. **Curr.Opin.Struct.Biol.** *15*: 401-407

Whelan, J. (2002). **Drug Discovery Today**. *7(2),* 90-92.

Young H., Baum R., Cremerius U., Herholz K., Hoekstra O., Lammertsma A.A., Pruim J., Price P. (1999) Measurement of clinical and subclinical tumour response using [18F]-fluorodeoxyglucose and positron emission tomography: review and 1999 EORTC recommendations. European Organization for Research and Treatment of Cancer (EORTC) PET Study Group. **Eur J Cancer**. Dec;35(13):1773-82.

Yuan, F., Salehi, H.A., Boucher, Y., Vasthare, U.S., Tuma, R.F., Jain, R.K. (1994). Microvascular permeability and interstitial penetration of sterically stabilized (stealth) liposomes in a human tumour xenograft. **Cancer Res**. *54:* 3352–3356.

Zimmerman, M., Zouhair, A., Azria, D., Ozsahin, A. (2006). The epidermal growth factor receptor (EGFR) in head and neck cancer: its role and treatment implications. **Radiat Oncol**. *1*: 11.

14 APPENDICES

Appendix A. Schedule of Assessments

Whole of Study Assessments for Part 1

Study Procedures	Screen Visit/s	Pot. 2nd Screen Visit	Cycle 1											Subsequent Cycles						F/Up[6]
Day	≤ -21	≤-10[1]	1		8		15		22		29		36-42[2]	1[3]	8	15	22	29	36-42[2]	30 post dose
Permissible Windows (days)					± 2[4]		± 2[4]		± 2[4]		± 2[4]			± 2[5]	± 2[4]	± 2[4]	± 2[4]	± 2[4]		+5
Time Points (hrs)			0-6	24	0-6	24	0-6	24	0-6	24	0-6	24		0-4	0-4	0-4	0-4	0-4		
Informed Consent	X																			
Eligibility Criteria	X																			
Medical History	X																			
Medication History	X																			
Weight	X		X		X		X		X		X			X	X	X	X	X		X
Height	X																			
Resting Pulse, Resp. and Temp	X		X	X	X	X	X	X	X	X	X	X		X	X	X	X	X		X
Blood Pressure	X		X	X	X	X	X	X	X	X	X	X		X	X	X	X	X		X
ECOG Performance Status	X		X		X		X		X		X			X	X	X	X	X		X
Physical Examination	X		X		X		X		X		X			X	X	X	X	X		X
ECG	X		X	X	X	X	X	X	X	X	X	X		X	X	X	X	X		X
Changes to Concomitant Meds			X	X	X	X	X	X	X	X	X	X		X	X	X	X	X		X
Adverse Events			X	X	X	X	X	X	X	X	X	X		X	X	X	X	X		X
**Laboratory Assessments**																				
Blood Chemistry		X	X	X	X	X	X	X	X	X	X	X		X	X	X	X	X		X
Full Blood Count		X	X	X	X	X	X	X	X	X	X	X		X	X	X	X	X		X
Coagulation		X	X	X	X	X	X	X	X	X	X	X		X	X	X	X	X		X
Creatinine Clearance[7]		X	X	X	X	X	X	X	X	X	X	X		X	X	X	X	X		X
Salmonella & Erbitux ® antibodies	X		X		X		X		X		X			X	X	X	X	X		X
Urinalysis		X	X	X	X	X	X	X	X	X	X	X		X	X	X	X	X		X
Serum Pregnancy Test		X																		
Immune Response			X	X	X	X	X	X	X	X	X	X		X	X	X	X	X		
Inflammatory Response			X	X	X	X	X	X	X	X	X	X		X	X	X	X	X		
PK Samples			X	X	X	X	X	X	X	X	X	X								
**Radiological Assessments**																				
CT scan	X												X						X	
**Erbitux® EDVs$_{pac}$ Administration[8]**			X		X		X		X		X			X	X	X	X	X		

See next page for footnotes ...

1. Screening laboratory tests should be performed within 10days of the first dose. A second screening visit is only needed if all screening procedures cannot be performed within 10 days of first dose.
2. Restaging should occur 1 to 7 days before the first dose of each cycle and 7-14 days after the last dose of the final cycle. If a cycle is delayed the restaging should occur 7-14 days after the last dose of the previous cycle.
3. Day 1 of subsequent cycles should occur 14 days after the last dose of the previous cycle.
4. Minimum 5 days and maximum 9 days between doses within a cycle. However, as much as possible the gap between doses should be kept to **7** days.
5. If the patient experiences grade 3 toxicities, the start of the subsequent cycle may be delayed by up to 21 days to allow for recovery.
6. Follow up visit to occur between 30 and 35 days after final dose administration. If a patient discontinues treatment before the end of the first cycle, an additional follow up visit should be conducted within 14 days after their last dose
7. Only to be calculated if patients' creatinine levels are elevated.
8. Including administration of pre-medication with dexamethasone 8mg i.v. and promethazine 12.5 - 25mg i.v or promethazine 25mg orally or loratidine 20mg orally and Paracetamol 1g orally.

Whole of Study Assessments for Part 2

**Study Procedures**	**Screen Visit/s**	**Pot. 2nd Screen Visit**	**Cycle 1**											**Subsequent Cycles**						**F/Up[6]**
Day	≤ -21	≤-10[1]	1		8		15		22		29		36-42[2]	1[3]	8	15	22	29	36-42[2]	30 post dose
Permissible Windows (days)					± 2[4]		± 2[4]		± 2[4]		± 2[4]			± 2[5]	± 2[4]	± 2[4]	± 2[4]	± 2[4]		+5
Time Points (hrs)			0-6	24	0-6	24	0-6	24	0-6	24	0-6	24		0-4	0-4	0-4	0-4	0-4		
Informed Consent	X																			
Eligibility Criteria	X																			
Medical History	X																			
Medication History	X																			
Weight	X		X		X		X		X		X			X	X	X	X	X		X
Height	X																			
Resting Pulse, Resp. and Temp	X		X	X	X	X	X	X	X	X	X	X		X	X	X	X	X		X
Blood Pressure	X		X	X	X	X	X	X	X	X	X	X		X	X	X	X	X		X
ECOG Performance Status	X		X		X		X		X		X			X	X	X	X	X		X
Physical Examination	X		X		X		X		X		X			X	X	X	X	X		X
ECG	X		X	X	X	X	X	X	X	X	X	X		X	X	X	X	X		X
Changes to Concomitant Meds			X	X	X	X	X	X	X	X	X	X		X	X	X	X	X		X
Adverse Events			X	X	X	X	X	X	X	X	X	X		X	X	X	X	X		X
**Laboratory Assessments**																				
Blood Chemistry		X	X	X	X	X	X	X	X	X	X	X		X	X	X	X	X		X
Full Blood Count		X	X	X	X	X	X	X	X	X	X	X		X	X	X	X	X		X
Coagulation		X	X	X	X	X	X	X	X	X	X	X		X	X	X	X	X		X
Creatinine Clearance[7]		X																		
Salmonella & Erbitux ® antibodies	X		X		X		X		X		X			X	X	X	X	X		X
Urinalysis		X	X	X	X	X	X	X	X	X	X	X		X	X	X	X	X		X
Serum Pregnancy Test		X																		
Immune Response			X	X	X	X	X	X	X	X	X	X		X	X	X	X	X		
Inflammatory Response			X	X	X	X	X	X	X	X	X	X		X	X	X	X	X		
PK Samples			X	X	X	X	X	X	X	X	X	X								
EGFR Expression[8]	X																			
**Radiological Assessments**																				
CT scan	X												X						X	
FDG PET scan	X												X							
**Erbitux® EDVs$_{Pac}$ Administration[9]**			X		X		X		X		X			X	X	X	X	X		

See next page for footnotes ...

1. Screening laboratory tests should be performed within 10 days of the first dose. A second screening visit is only needed if all screening procedures cannot be performed within 10 days of first dose.
2. Restaging should occur 1 to 7 day before the first dose of each cycle and 7-14 days after the last dose of the final cycle. If a cycle is delayed the restaging should occur 7-14 days after the last dose of the previous cycle.
3. Day 1 of subsequent cycles should occur 14 days after the last dose of the previous cycle.
4. Minimum 5 days and maximum 9 days between doses within a cycle. However, as much as possible the gap between doses should be kept to **7** days.
5. If the patient experiences grade 3 toxicities, the start of the subsequent cycle may be delayed by up to 21 days to allow for recovery.
6. Follow up visit to occur between 30 and 35 days after final dose administration. If a patient discontinues treatment before the end of the first cycle, an additional follow up visit should be conducted within 14 days after their last dose
7. Only to be calculated if patients' creatinine levels are elevated.
8. Only if patients do not already have documented evidence of EGFR expression.
9. Including administration of pre-medication with dexamethasone 8mg i.v. and promethazine 12.5 - 25mg i.v. or promethazine 25mg orally or loratidine 20mg orally and Paracetamol 1g orally.

Dosing Visit Assessments for Parts 1 and 2

Permissible Window	Pre-Dose	0 hr	1hr ± 5min	2hr ± 10min	4hr ± 15min	6hr[1] ± 15min	24hr[1] ± 1hr
Weight	X						
Resting Pulse, Resp and Temp[2]	X		X	X	X	X	X
Blood Pressure	X		X	X	X	X	X
ECOG Performance Status	X						
Physical Examination	X[3]						
ECG	X				X		X
Recording Changes to Concomitant	X[3]	→	→	→	→	→	→
Recording Adverse Events	X[3]	→	→	→	→	→	→
**Laboratory Assessments**							
Blood Chemistry	X				X		X
Full Blood Count	X				X		X
Coagulation	X				X		X
Urinalysis	X				X		X
PK Samples[4]	X				X		X
Immune response	X				X		X
Inflammatory response	X				X		X
Salmonella & Erbitux® antibodies	X						
Administration of Erbitux® EDVs$_{Pac}$		X					
Administration of premedication	X						

1. The 6 and 24 hour time points apply only in the patient's first cycle.
2. If temperature elevated repeat 4 hourly (if supplementary to scheduled assessments) until temperature returns to normal
3. At the pre-dose assessment changes since the last visit will be recorded
4. PK samples only apply to the patients' first cycle

Appendix B. ECOG Performance Status Scale

Grade	Description
0	Fully active, able to carry on all pre-disease performance without restriction
1	Restricted in physically strenuous activity but ambulatory and able to carry out work of a light or sedentary nature, e.g., light house work, office work
2	Ambulatory and capable of all self-care but unable to carry out any work activities. Up and about more than 50% of waking hours
3	Capable of only limited self-care, confined to bed or chair more than 50% of waking hours
4	Completely disabled. Cannot carry on any self-care. Totally confined to bed or chair
5	Dead

Appendix C. Cockcroft Gault Formula for Calculated Creatinine Clearance

Estimated creatinine clearance in mL/min:

In men = $\dfrac{(140 - \text{age}) \times \text{weight (kg)}}{0.814 \times [\text{serum creatinine}(\mu\text{mol/L}]}$

In women = 0.85 x creatinine clearance in men

Appendix D. Adverse Event Assessments

Adverse Event Severity Scoring System

The Common Terminology Criteria for Adverse Events (CTCAE) is available at the following link:

http://ctep.cancer.gov/protocolDevelopment/electronic_applications/docs/ctcaev3.pdf

If an adverse event cannot be graded by the CTCAE version 3, the following severity grades may be used:

Grade	Adverse Event Severity Scoring System
1	**Mild:** Aware of the sign or symptom, but easily tolerated
2	**Moderate:** Discomfort enough to cause interference with usual activity
3	**Severe:** Incapacitating with inability to do work or do usual activity
4	**Life-threatening:** Refers to an event in which the patient was in the view of the investigator, at risk of death at the time, and in the severity, of the event as it occurred.
5	**Fatal**

Appendix E. RECIST Criteria

European Journal of Cancer 45 (2009) 228-247

New response evaluation criteria in solid tumours: Revised RECIST guideline (version 1.1)

Appendix F. Cytochrome P450 inducers/inhibitors

The following list of inhibitors of CYP3A4 and 2C8 is extracted from the following website:

http://medicine.iupui.edu/flockhart/table.htm

	CYP3A4,5,7	2C8
Strong Inhibitors<br>*cause a > 5-fold increase in the plasma AUC values or more than 80% decrease in clearance*	indinavir<br>nelfinavir<br>ritonavir<br>clarithromycin<br>itraconazole<br>ketoconazole<br>nefazodone<br>saquinavir<br>telithromycin	gemfibrozil
Moderate Inhibitors<br>*cause a > 2-fold increase in the plasma AUC values or 50-80% decrease in clearance*	aprepitant<br>erythromycin<br>fluconazole<br>grapefruit juice<br>verapamil<br>diltiazem	trimethoprim
Weak or Other Inhibitors<br>*all other inhibitors*	cimetidine<br>amiodarone<br>azithromycin<br>chloramphenicol<br>delaviridine<br>diethyl-dithiocarbamate<br>fluvoxamine<br>gestodene<br>imatinib<br>mibefradil<br>mifepristone<br>norfloxacin<br>norfluoxetine<br>star fruit<br>voriconazole	glitazones<br>montelukast<br>quercetin
