# Supplementary material for: A First-Time-In-Human Phase I Clinical Trial of Bispecific Antibody-Targeted, Paclitaxel-Packaged Bacterial Minicells
Source: PLoS One. 2015 Dec 11;10(12):e0144559. doi: 10.1371/journal.pone.0144559 (PMC4699457; doi:10.1371/journal.pone.0144559)
Supplement: S1 TREND Checklist — (PDF) [file pone.0144559.s007.pdf]

TREND Statement Checklist

Paper Section/ Topic	Item No	Descriptor	Reported? ✓	Pg #
**Title and Abstract**				
Title and Abstract	1	Information on how unit were allocated to interventions	✓	Abstract
		Structured abstract recommended	✓	Abstract
		Information on target population or study sample	✓	Abstract
**Introduction**				
Background	2	Scientific background and explanation of rationale	✓	Introduction
		Theories used in designing behavioral interventions	NA	
**Methods**				
Participants	3	Eligibility criteria for participants, including criteria at different levels in recruitment/sampling plan (e.g., cities, clinics, subjects)	✓	Methods: Patients
		Method of recruitment (e.g., referral, self-selection), including the sampling method if a systematic sampling plan	✓	Methods: Patients
		Recruitment setting	✓	Methods: Study Design
		Settings and locations where the data were collected	✓	Methods: Study Design
Interventions	4	Details of the interventions intended for each study condition and how and when they were actually administered, specifically	✓	Methods
		○ Content: what was given?	✓	Methods: Study Procedures
		○ Delivery method: how was the content given?	✓	Methods: Study Procedures
		○ Unit of delivery: how were the subjects grouped during delivery?	✓	Methods: Study Procedures
		○ Deliverer: who delivered the intervention?	✓	Methods: Study Procedures
		○ Setting: where was the intervention delivered?	✓	Methods: Study Design
		○ Exposure quantity and duration: how many sessions or episodes or events were intended to be delivered? How long were they intended to last?	✓	Methods: Study Procedures
		○ Time span: how long was it intended to take to deliver the intervention to each unit?	✓	Methods: Study Procedures
		○ Activities to increase compliance or adherence (e.g.,	NA	
Objectives	5	Specific objectives and hypotheses	✓	Methods: Study Design
Outcomes	6	Clearly defined primary and secondary outcome measures	✓	Methods: Assessments
		Methods used to collect data and any methods used to enhance the quality of measurements	✓	Methods: Assessments and Lab Studies
		Information on validated instruments such as psychometric and biometric properties	NA	
Sample Size	7	How sample size was determined and, when applicable, explanation of any interim analyses and stopping rules	✓	Methods: Study Procedures

TREND Statement Checklist

Assignment Method	8	Unit of assignment (the unit being assigned to study condition, e.g., individual, group, community)	✓	Methods: Study Procedures
		Method used to assign units to study conditions, including details of any restriction (e.g., blocking, stratification, minimization)	✓	Methods: Study Procedures
		Inclusion of aspects employed to help minimize potential bias induced due to non-randomization (e.g., matching)	NA	
Blinding (masking)	9	Whether or not participants, those administering the interventions, and those assessing the outcomes were blinded to study condition assignment; if so, statement regarding how the blinding was accomplished and how it was assessed.	NA	
Unit of Analysis	10	Description of the smallest unit that is being analyzed to assess intervention effects (e.g., individual, group, or	✓	Methods: Study Procedures
		If the unit of analysis differs from the unit of assignment, the analytical method used to account for this (e.g., adjusting the standard error estimates by the design effect or using multilevel	NA	
Statistical Methods	11	Statistical methods used to compare study groups for primary methods outcome(s), including complex methods of correlated	✓	Methods: Statistics
		Statistical methods used for additional analyses, such as a subgroup analyses and adjusted analysis		Methods: Statistics
		Methods for imputing missing data, if used	NA	
		Statistical software or programs used	✓	Methods: Statistics
**Results**				
Participant flow	12	Flow of participants through each stage of the study: enrollment, assignment, allocation, and intervention exposure, follow-up, analysis (a diagram is strongly recommended)	✓	Fig.2
		○ Enrollment: the numbers of participants screened for eligibility, found to be eligible or not eligible, declined to be enrolled, and	✓	Fig.2
		○ Assignment: the numbers of participants assigned to a study condition	✓	Table 2
		○ Allocation and intervention exposure: the number of participants assigned to each study condition and the number of participants	✓	Table 2
		○ Follow-up: the number of participants who completed the follow- up or did not complete the follow-up (i.e., lost to follow-up), by study condition	✓	Fig.2
		○ Analysis: the number of participants included in or excluded from the main analysis, by study condition	✓	Fig.2
		Description of protocol deviations from study as planned, along with reasons	✓	Results: Dose Limiting toxicities
Recruitment	13	Dates defining the periods of recruitment and follow-up	✓	Results: Patient Characteristics
Baseline Data	14	Baseline demographic and clinical characteristics of participants in each study condition	✓	Table 1
		Baseline characteristics for each study condition relevant to specific disease prevention research	NA	
		Baseline comparisons of those lost to follow-up and those retained, overall and by study condition	NA	

TREND Statement Checklist

		Comparison between study population at baseline and target population of interest	NA	
Baseline equivalence	15	Data on study group equivalence at baseline and statistical methods used to control for baseline differences	NA	
Numbers analyzed	16	Number of participants (denominator) included in each analysis for each study condition, particularly when the denominators change for different outcomes; statement of the results in absolute	✓	Fig 2
		Indication of whether the analysis strategy was "intention to treat" or, if not, description of how non-compliers were treated in the analyses	NA	
Outcomes and estimation	17	For each primary and secondary outcome, a summary of results for each estimation study condition, and the estimated effect size and a confidence interval to indicate the precision	NA	
		Inclusion of null and negative findings	NA	
		Inclusion of results from testing pre-specified causal pathways through which the intervention was intended to operate, if any	NA	
Ancillary analyses	18	Summary of other analyses performed, including subgroup or restricted analyses, indicating which are pre-specified or	NA	
Adverse events	19	Summary of all important adverse events or unintended effects in each study condition (including summary measures, effect size estimates, and confidence intervals)	✓	Results: Adverse events
**DISCUSSION**				
Interpretation	20	Interpretation of the results, taking into account study hypotheses, sources of potential bias, imprecision of measures, multiplicative analyses.	✓	Discussion
		Discussion of results taking into account the mechanism by which the intervention was intended to work (causal pathways) or alternative mechanisms or explanations	✓	Discussion
		Discussion of the success of and barriers to implementing the intervention, fidelity of implementation	✓	Discussion
		Discussion of research, programmatic, or policy implications	✓	Discussion
Generalizability	21	Generalizability (external validity) of the trial findings, taking into account the study population, the characteristics of the intervention, length of follow-up, incentives, compliance rates, specific sites/settings involved in the study, and other contextual issues	✓	Discussion
Overall Evidence	22	General interpretation of the results in the context of current evidence and current theory	✓	Discussion

From: Des Jarlais, D. C., Lyles, C., Crepaz, N., & the Trend Group (2004). Improving the reporting quality of nonrandomized evaluations of behavioral and public health interventions: The TREND statement. *American Journal of Public Health*, 94, 361-366. For more information, visit: http://www.cdc.gov/trendstatement/
